# DYRK1A inhibition and cognitive rescue in a Down syndrome mouse model are induced by new fluoro-DANDY derivatives

**DOI:** 10.1038/s41598-018-20984-z

**Published:** 2018-02-12

**Authors:** Fernanda Neumann, Stéphanie Gourdain, Christelle Albac, Alain D. Dekker, Linh Chi Bui, Julien Dairou, Isabelle Schmitz-Afonso, Nathalie Hue, Fernando Rodrigues-Lima, Jean M. Delabar, Marie-Claude Potier, Jean-Pierre Le Caër, David Touboul, Benoît Delatour, Kevin Cariou, Robert H. Dodd

**Affiliations:** 1 Institut de Chimie des Substances Naturelles, CNRS UPR 2301, Université Paris-Sud, Université Paris-Saclay, Avenue de la Terrasse, 91198 Gif-sur-Yvette, France; 2 Sorbonne Universités, Université Pierre et Marie Curie (UPMC), Université Paris 06, Institut National de la Santé et de la Recherche Médicale (INSERM) and Centre National de la Recherche Scientifique (CNRS) Unités de Recherche U75, U1127, U7225, and Institut du Cerveau et de la Moelle Epinière (ICM), 75013 Paris, France; 3 Department of Neurology, University of Groningen, University Medical Center Groningen (UMCG), Hanzeplein 1, 9713 GZ Groningen, The Netherlands; 4 Université Paris Diderot, Sorbonne Paris Cité, Unité BFA, CNRS UMR 8251, 75013 Paris, France; 5 UMR 8601 CNRS, Laboratoire de Chimie et Biochimie Pharmacologiques et Toxicologiques, Université Paris Descartes-Sorbonne Paris Cité, 75270 Paris, France

## Abstract

Inhibition of DYRK1A kinase, produced by chromosome 21 and consequently overproduced in trisomy 21 subjects, has been suggested as a therapeutic approach to treating the cognitive deficiencies observed in Down syndrome (DS). We now report the synthesis and potent DYRK1A inhibitory activities of fluoro derivatives of 3,5-di(polyhydroxyaryl)-7-azaindoles (F-DANDYs). One of these compounds (3-(4-fluorophenyl)-5-(3,4-dihydroxyphenyl)-1*H*-pyrrolo[2,3-*b*]pyridine, **5a**) was selected for *in vivo* studies of cognitive rescuing effects in a standard mouse model of DS (Ts65Dn line). Using the Morris water maze task, Ts65Dn mice treated i.p. with 20 mg/kg of **5a** performed significantly better than Ts65Dn mice treated with placebo, confirming the promnesiant effect of **5a** in the trisomic mice. Overall, these results demonstrate for the first time that selective and competitive inhibition of DYRK1A kinase by the F-DANDY derivative **5a** may provide a viable treatment strategy for combating the memory and learning deficiencies encountered in DS.

## Introduction

Down syndrome (DS), or trisomy 21, is the most common genetically acquired form of intellectual disability^1–3^, occurring in approximately 1 out of 650–1000 newborns in Europe^4,5^ and North America^6^. In addition to their characteristic physical appearance, people with DS present developmental neurological delay, including a lower IQ^7^ and reduced learning and memory capacities^3^. DS, for which there is currently no cure, is caused by the presence of an extra copy of chromosome 21 leading to increased production and resulting imbalance of the proteins and enzymes encoded by this chromosome^8,9^. One of these enzymes is the dual-specificity tyrosine phosphorylation kinase 1a, or DYRK1A, belonging to the CGMC kinome group and which is expressed in all mammalian tissues but especially so in the developing brain^10–12^. DYRK1A is implicated in cell proliferation^13^ and neuronal development^14^ as well as a wide range of signaling pathways. In DS, the triplication of chromosome 21 leads to approximately 1.5-fold higher DYRK1A levels compared to the general euploid population^15^ and this overproduction has been linked to the cognitive deficits associated with DS^16,17^, and notably to imbalance of excitation/inhibition^18^. Through hyperphosphorylation of Tau protein^19^ and the resulting formation of insoluble tau aggregates and neurofibrillary tangles, DYRK1A is also involved in neurodegeneration and neuronal loss appearing in Alzheimer’s disease (AD)^20,21^. DYRK1A has been found to be abnormally expressed in both DS and AD^22^ and indeed, people with DS develop AD precociously^23^, the amyloid precursor protein (APP) at the origin of senile plaques also being overexpressed by chromosome 21 in DS individuals^24^.

**Figure 1:**
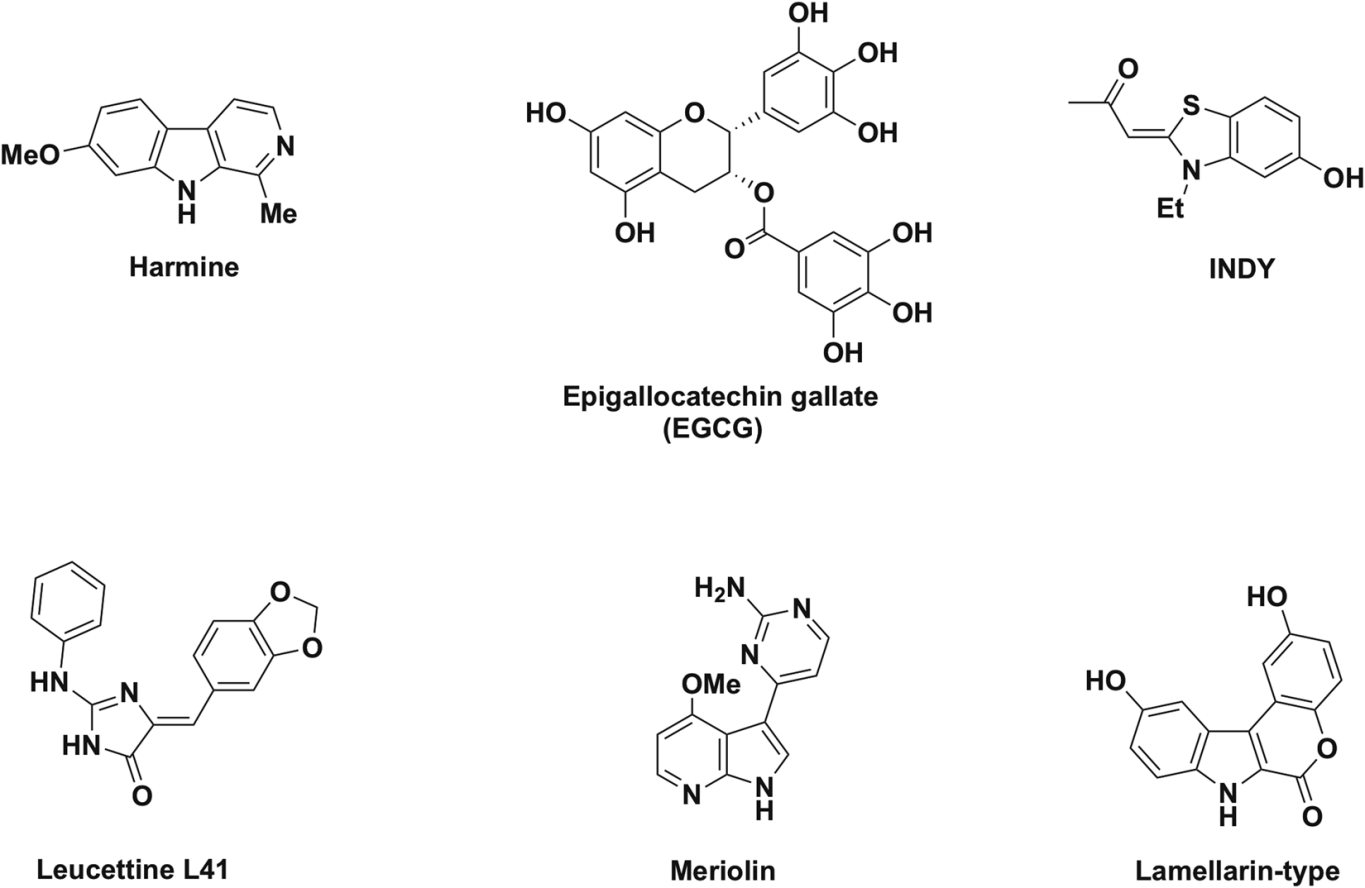
Naturally-occurring and synthetic inhibitors of DYRK1A kinase.

A plausible therapeutic strategy for cognitive deficits associated with DS and eventually AD would thus entail controlled inhibition of the activity of cerebral DYRK1A kinase^25^. To this end, a variety of DYRK1A inhibitors has been developed over the past few years most of which bind to the active ATP site of the enzyme. Examples of such competitive inhibitors of DYRK1A are shown in Fig. 1. These include harmine, an alkaloid isolated from *Peganum harmala*^26^, the INDY derivatives^27^, the leucettine derivative L41^28^, as well as analogues of the naturally-occurring (aza) indolic compounds meriolin^29^ and lamellarin^30^. While the leucettine derivative L41 was shown to prevent memory impairment produced by administration of the β-amyloid peptide Aβ_25–35_ in rodents^31^, to the best of our knowledge, none of the current competitive DYRK1A inhibitors has passed the *in vitro* stage of investigation with respect to improvement of cognitive impairments in DS. In contrast, epigallocatechin gallate (EGCG), the major active principle of green tea, has been demonstrated to be a relatively potent allosteric inhibitor of DYRK1A^12,32^ and to produce cognitive enhancement in Ts65Dn mice, the most widely used mouse model for DS^33^.

**Figure 2:**
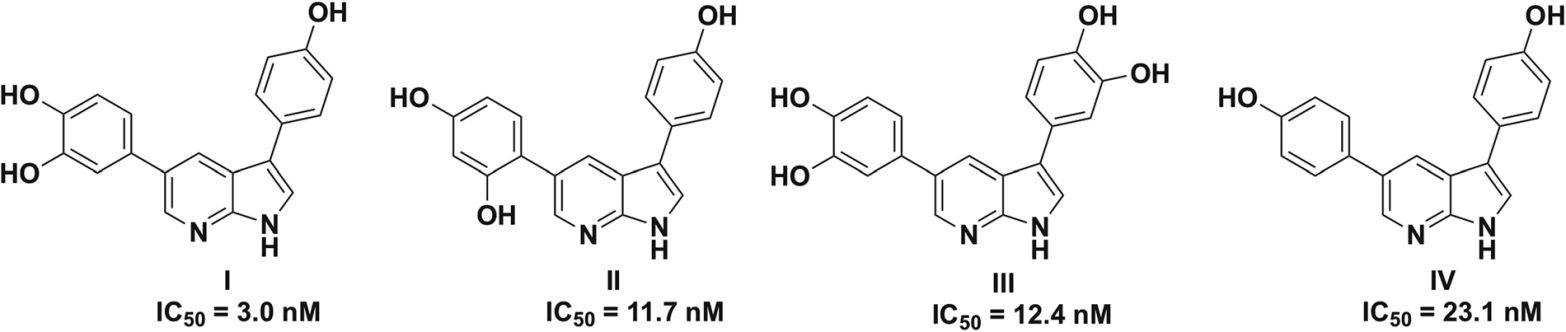
Structures of the most active DYRK1A competitive inhibitors of the 3,5-diaryl-7-indole family (DANDYs)^34^.

We recently reported that hydroxy derivatives of 3,5-diaryl-7-azaindoles (DANDYs) were potent, competitive inhibitors of DYRK1A^34^. The di-, tri- and tetrahydroxy diaryl azaindoles **I**-**IV** displayed *in vitro* inhibition of this kinase with IC_50_s in the 3 to 23 nM range (Fig. 2) and selectivity with respect to a panel of structurally related kinases including DYRK2 and DYRK3. Starting from the known resolved crystal structure of DYRK1A^27^, molecular modeling and docking studies of compounds **I**-**IV** revealed an extended network of hydrogen bonds between these heterocycles and the amino acid residues of the active site, accounting for their high inhibitory potency *in vitro*.

Because the large number of polar hydroxy groups in compounds **I**-**IV** might interfere with brain penetration or be a source of rapid metabolization, we initiated a study whereby one or two of the hydroxy groups were systematically replaced by fluorine atoms. In this report, we describe the synthesis of fluoro analogues of compounds **I**-**IV** (F-DANDYs) and their *in vitro* DYRK1A inhibitory activities. Moreover, for a selected, active F-DANDY (compound **5a**) administered to mice, we demonstrate, using mass spectral analysis of plasma and brain tissue, that this compound is stable *in vivo* and enters the brain in therapeutically relevant quantities. Finally, preliminary studies showed that this F-DANDY compound **5a** significantly improved the performance of Ts65Dn mice in the Morris water maze, a standard learning and memory paradigm for rodents, but had no observable effect on wild type mice.

## Results and Discussion

### Chemistry

**Figure 3:**
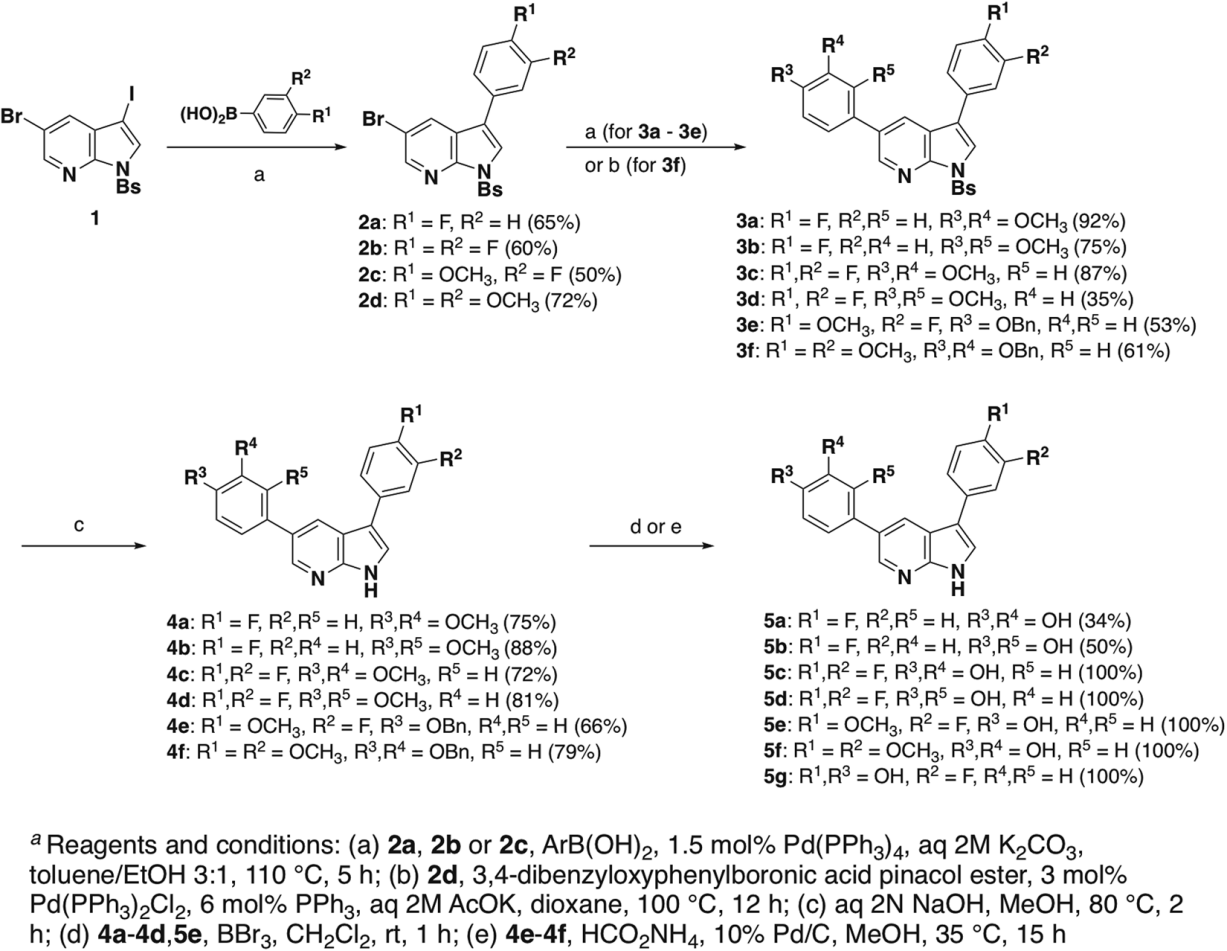
Chemical synthesis of the target substituted 3,5-diaryl-7-azaindole derivatives **5a**-**5g**^a^.

The synthetic strategy used to prepare the fluorinated or selectively *O*-methylated 3,5-diaryl-7-azaindoles **5a**-**5g** was essentially identical to the one we used for the synthesis of our first series of DANDYs^34^. Thus, as illustrated in Fig. 3, *N*-phenylsulfonyl-3-iodo-5-bromo-7-azaindole **1**, easily prepared from 5-bromo-7-azaindole^34^, was subjected to a first Suzuki-Miyaura coupling^35^ with 4-fluoro- or 3,4-difluorophenylboronic acid to provide good yields of the corresponding C-3-aryl 7-azaindoles **2a** and **2b**, respectively. No products resulting from coupling at the less reactive C-5 position were observed. Similarly, Suzuki coupling of **1** with 3-fluoro-4-methoxy- and 3,4-dimethoxyphenylboronic acid gave only the C-3 mono-coupled products **2c** and **2d**, respectively. Using the same reaction conditions, compounds **2a**-**2d** then served as starting materials for the second Suzuki-Miyaura coupling, this time with 3,4-dimethoxy-, 2,4-dimethoxy or 4-benzyloxyphenylboronic acid, giving access to the 3,5-diaryl-7-azaindoles **3a**-**3e** in mostly good to excellent yields. These conditions did not allow, however, coupling of **2d** with 3,4-dibenzyloxyphenylboronic acid. This was circumvented by coupling **2d** with 3,4-dibenzyloxyphenylboronic acid pinacol ester using dichlorobis(triphenylphosphine)palladium(II) and triphenylphosphine^36^ in dioxane as catalytic system, providing the desired product **3f** in 61% yield.

The *N*-phenylsulfonyl protecting groups of compounds **3a**-**3f** were efficiently removed by the action of aqueous NaOH in methanol, providing the 3,5-diaryl-7-azaindoles **4a**-**4f** in yields ranging from 66% (**4e**) to 88% (**4b**). De-*O*-methylation of the 7-azaindole derivatives **4a**-**4d** with excess boron tribromide in dichloromethane at room temperature then provided the first of the targeted F-DANDY analogues, the mono- and difluorinated products **5a-5d**. Hydrogenolytic cleavage of the *O*-benzyl groups of **4e** and **4f** using ammonium formate and

palladium black in methanol gave access to the mixed hydroxy/methoxy F-DANDY analogues **5e** and **5f**, respectively, in quantitative yields. Finally, treatment of **5e** with BBr_3_ in CH_2_Cl_2_ furnished **5g**, the monofluorinated analogue of the highly active DYRK1A inhibitors 3,5-di-(4-hydroxyphenyl)-7-azaindole (**IV**) and 3-(4-hydroxyphenyl)-5-(3,4-dihydroxyphenyl)-7-azaindole (**I**).

### Inhibition of DYRK1A activity *in vitro*

All the newly synthesized *N*-deprotected F-DANDY analogues were evaluated for their *in vitro* DYRK1A inhibitory activity using a fluorescent peptide substrate of this enzyme and UFLC (Ultra Fast Liquid Chromatography) assay as previously described^34,37^. We first tested the fluorinated analogues **4a**-**4e** having only methoxy or benzyloxy groups instead of free phenolic hydroxy functions on the C-3 and C-5-phenyl rings and compared the results to our previously reported non-fluorinated methoxy derivatives **MeO-I** to **MeO-IV**.

**Figure 4:**
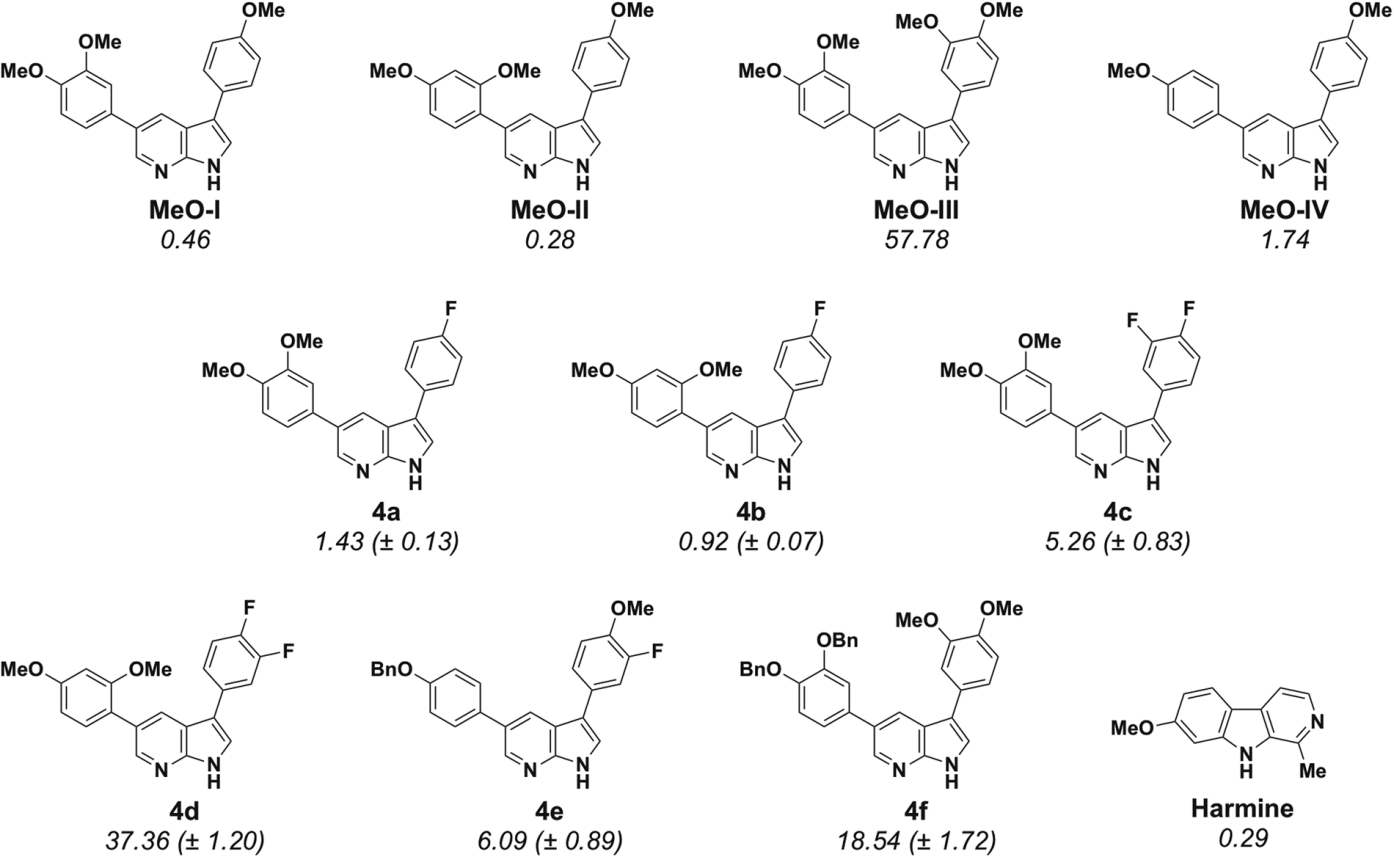
*In Vitro* inhibition of DYRK1A by 3,5-diaryl-7-azaindoles **4a**-**4f** compared to activities of previously reported DANDYs (IC_50_ values in μM)^34^.

As shown in Fig. 4, the IC_50_s of these compounds, fluorinated or not, were mostly in the micromolar range. In general, it appears that introduction of one or two fluorine atoms on the C-3 phenyl ring has little effect on DYRK1A inhibitory activity. Thus, replacement of one of the methoxy groups of **MeO-I** and **MeO-II** by a fluorine atom (compounds **4a** and **4b**) produced a 3-fold loss in activity (IC_50_ = 0.46 μM and 0.28 μM compared to IC_50_ = 1.43 μM and 0.92 μM for the fluorinated analogues, respectively). On the other hand, replacement of two methoxy groups of compound **MeO-III** (IC_50_ = 57.78 μM) by two fluorine atoms (**4c**, IC_50_ = 5.26 μM) led to a 10-fold improvement in DYRK1A inhibition. Interestingly, introduction of a supplementary fluorine atom at the C-3 position of the C-4 fluoro derivative **4b** (to give **4d**) led to an almost 40-fold loss of activity (IC_50_ = 37.36 μM for the 3,4-difluoro compound compared to 0.92 μM for the 4-fluoro analogue). This effect of an additional fluorine atom was much less evident when comparing the 3,4-difluoro derivative **4c** with the 4-fluoro analogue **4a** (both differing only in the position of a methoxy group with respect to **4b** and **4d**) which displayed only a 4-fold diminishment of inhibitory activity (**4c**: IC_50_ = 5.26 μM; **4a**: IC_50_ = 1.43 μM). Finally, our attention turned to the benzyloxy derivatives **4e** and **4f** which were prepared in order to allow orthogonal protection of the phenolic hydroxy groups. While the mono-benzyloxy derivative **4e** was equipotent with the dimethoxy compound **4c** (IC_50_ = 6.09 μM and 5.26 μM, respectively), replacement of the 3′,4′-dimethoxy groups of **MeO-III** by dibenzyloxy groups as in **4f** surprisingly led to a 3-fold superior activity though both compounds can be considered to be among the least active of our study (**4f**: IC_50_ = 18.54 μM; **MeO-III**: IC_50_ = 57.78 μM).

**Figure 5:**
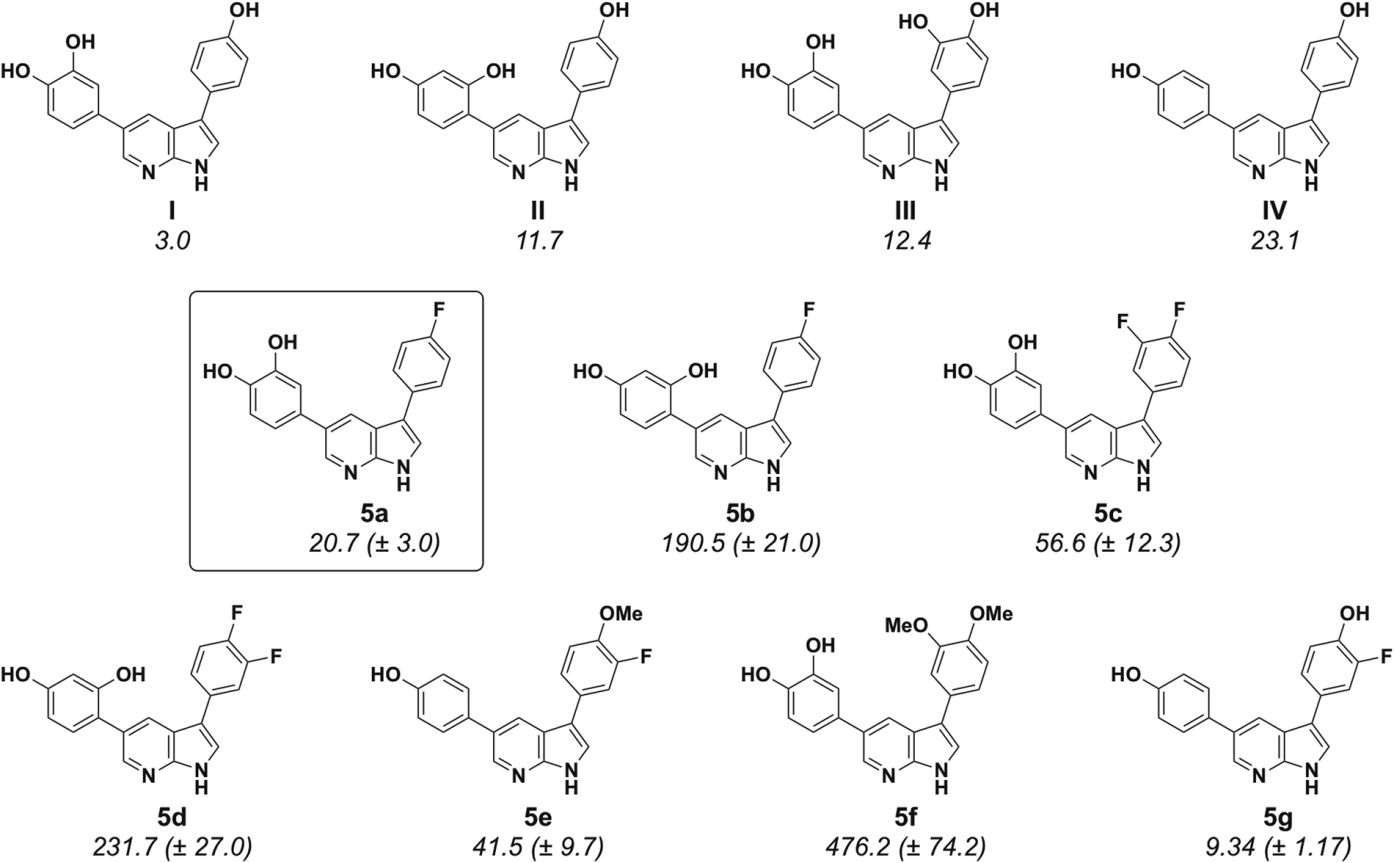
*In Vitro* inhibition of DYRK1A by 3,5-diaryl-7-azaindoles **5a**-**5g** compared to activities of previously reported DANDYs (IC_50_ values in nM)^34^.

We then went on to evaluate the DYRK1A inhibitory activities of the de-*O*-protected analogues of **4a**-**4f**, that is, compounds **5a**-**5g**. As expected from the results of our previous study, the latter phenolic derivatives proved generally to be considerably more potent inhibitors than their *O*-methylated counterparts (Fig. 5, IC_50_ values given in nM). Following the same comparative analysis as for the *O*-methyl derivatives of Fig. 4, replacement of the C-4 hydroxy group of compound **I** by a fluorine atom (compound **5a**) led to a 7-fold decrease in inhibitory activity (IC_50_ = 3.0 nM and 20.7 nM, respectively). But this effect was much less deleterious than operating the same modification on compound **II**, the resulting analogue **5b** displaying this time an almost 20-fold loss of activity (IC_50_ = 11.7 nM and 190.5 nM, respectively).

Adding a fluorine atom to the C-3 positions of compounds **5a** and **5b**, yielding compounds **5c** and **5d**, led to a further loss of activity in both cases (IC_50_ = 56.6 nM and 231.7 nM, respectively). Interestingly, the 3-fluoro-4-methoxy derivative **5e** proved to be surprisingly active despite the presence of the methoxy group (IC_50_ = 41.5 nM) and this activity was gratifyingly improved almost 5-fold in the de-*O*-methylated counterpart, compound **5g** (IC_50_ = 9.34 nM), the most active F-DANDY of our study. Compound **5g** also represents the only example wherein simple addition of a fluorine atom to an active DANDY derivative led to a significant increase in activity. Thus, **5g** was 2.5-fold more active than compound **IV** (IC_50_ = 23.1 nM). Finally, another strong indication that free phenolic hydroxy groups are essential to high inhibitory activity was provided by the observation that the dimethoxy compound **5f** was 40 times less active than its phenolic equivalent **III** (IC_50_ = 476 nM and 12.4 nM, respectively).

### Cytotoxicity Evaluation

Because the potential cytotoxicity of the F-DANDY derivatives could account, at least in part, for their DYRK1A inhibition *in cellulo*, the cytotoxicities of compounds **5a** and **5g**, as well as of **5c** and **5e**, were evaluated *in vitro* for growth inhibition of KB cells^32^. As shown in Table 1, compounds **5a** and **5c** were essentially non-cytotoxic at a concentration of 10^−6^ M and only modestly cytotoxic at 10^−5^ M, inhibiting less than 50% of KB cell growth at the latter concentration. In contrast, compounds **5e** and **5g** were somewhat more cytotoxic, compound **5g** displaying the highest inhibitory potency with an estimated IC_50_ value of 10^−6^ M.

**Table 1:** Inhibition of KB Cell Proliferation *in vitro* by Selected F-DANDYs.

Compound	% Growth inhibition 10^−5^ M	% Growth inhibition 10^−6^ M
**5a**	49 ± 3	0 ± 9
**5c**	22 ± 2	6 ± 2
**5e**	66 ± 3	30 ± 1
**5** **g**	95 ± 1	46 ± 1

### Inhibition of tau phosphorylation by DYRK1A *in cellulo*

In order to verify that compound **5a** can penetrate cells and inhibit tau phosphorylation by DYRK1A *in cellulo*, we compared the activity of DYRK1A in the absence or in the presence of various concentrations of **5a** in human embryonic kidney cells (HEK293) transiently transfected with DYRK1A and tau. The activity of **5a** was compared to the activity of **5g** and harmine. Table 2 shows that all three compounds inhibited tau phosphorylation with IC_50_s in the 0.2 to 1 μM range. Ratios between *in cellulo* and *in vitro* inhibitory potencies were 0.6, 48 and 41 for harmine, compounds **5a** and **5g** respectively (Fig. 5 and Table 2). Compounds **5a** and **5g** showed comparable ratios indicating that the difference in cytotoxicity (**5g** being more cytotoxic than **5a**, Table 1) was not due to a difference in cell penetration and activity *in cellulo*.

**Table 2:** Inhibition of Tau Phosphorylation by DYRK1A by Selected DANDYs.

Compound	IC_50_ for tau phosphorylation (μM)
**5a**	1
**5g**	0.38
Harmine	0.19

### Kinase Profiling of 5a and 5g

**Figure 6:**
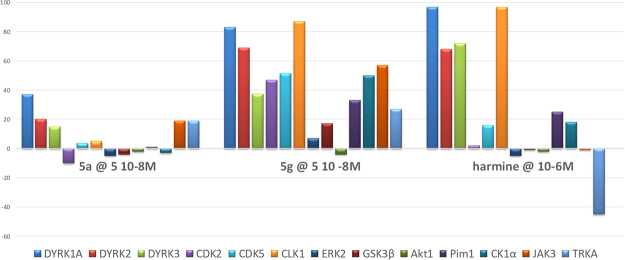
*In vitro* inhibition of a panel of 13 kinases by F-DANDYs **5a** and **5g** (5.10^−8^ M) and harmine (10^−6^ M) (100 represents full inhibition of the enzyme).

As for the DANDY derivatives **I-IV**^34^, the selectivity of F-DANDYs **5a** (the least cytotoxic) and **5g** (the most active) was evaluated at 5 × 10^−8^ M, together with harmine (at 10^−6^ M), on a panel of 13 kinases belonging to several families including DYRK (DYRK1A, DYRK2, DYRK3), the closely related CMGCs (CDK2, CDK5, CLK1, ERK2, GSK3β) as well as serine/threonine (AKT1, Pim1, CK1α) and tyrosine kinases (JAK3, TRKA) (Fig. 6). Neither **5a** nor **5g** was observed to be as selective as harmine. However, of these two F-DANDYs, **5a** was considerably more selective than **5g** for DYRK1A with respect to the other 12 kinases. As compared to harmine, **5a** did not show any activity on CLK1 and a higher selectivity for DYRK1A among the three DYRK tested.

Taken together, these results suggested that compound **5a** presented the best compromise in terms of DYRK1A inhibitory activity and selectivity as well as low cytotoxicity. Compound **5a** was thus selected for further study as described below.

### Mass Spectral Studies on Compound 5a

#### Bioavailability of 5a in plasma

**Figure 7:**
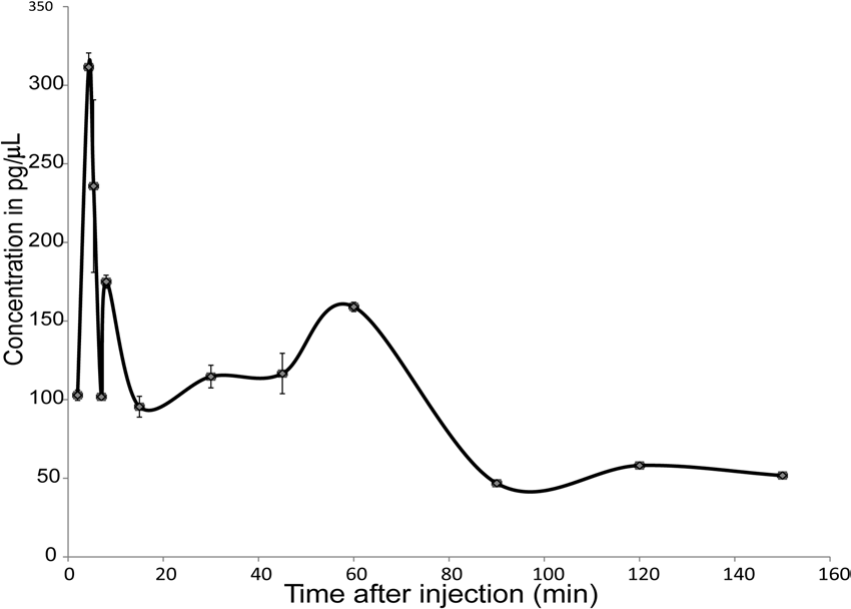
Evolution of compound **5a** concentration in plasma extracts after i.p. injection in WT mice. Standard deviations are noted after 3 injections except for points at 5.4, 8, 30 and 45 min where 3 extractions of each sample were realized.

Before undertaking behavioral studies in Ts65Dn mice, the bioavailability of F-DANDY **5a** in plasma after peripheral (intraperitoneal – i.p.) injection in mice was determined. UHPLC-MS/MS was used to detect and quantify compound **5a** in plasma of 12 wild-type (WT) mice that were treated with the drug (20 mg/kg; 2.4 mg/mL) and then sacrificed at 12 successive time points. The sample preparation and analytical protocol were adapted from Bonneau *et al*.^38,39^. As shown in Fig. 7, plasma concentrations of compound **5a** peaked a few minutes after injection (311.5 pg/μL at t = 4.4 min and 235.8 pg/μL at t = 5.4 min) and slowly decreased over time, reaching a plateau between 15 and 45 min. A 30 min interval was thus selected between time of injection and behavioral assessment.

#### Bioavailability of 5a in brain homogenates

Using UHPLC-MS/MS as described above, we detected and quantified compound **5a** in plasma and brain homogenates to assess whether it had likely crossed the blood-brain barrier. To that end, all animals were sacrificed directly after the Morris Water Maze (MWM) and plasma and brain homogenates (one hemisphere) were extracted and analyzed. Compound **5a**-treated Ts65Dn and WT mice had an average plasma concentration of 0.84 ± 0.54 μM (n = 9) and 0.50 ± 0.28 μM (n = 10), respectively, with no significant difference between Ts65Dn and WT mice. Mean brain concentrations were found to be 6.67 ± 4.64 μM (n = 5) and 6.61 ± 5.35 µM (n = 4) for Ts65Dn and WT mice, respectively, assuming a vascular space of 15 µL/g of tissue^40^. The partition coefficients at steady state (Kp = brain concentration/plasma concentration) for Ts65Dn and WT mice were 6.43 ± 3.38 and 10.81 ± 9.55 respectively, indicating that a substantial fraction of compound **5a** had crossed the blood-brain barrier. Compared to other drugs acting on the central nervous system, compound **5a** showed values similar to buproprion (4.97), fluoxetine (5.23) and perphenazine (7.12)^41^. However, Kp values might be underestimated due to high plasma levels that could saturate efflux transport to the brain.

### Behavioral Studies with Compound 5a

**Figure 8:**
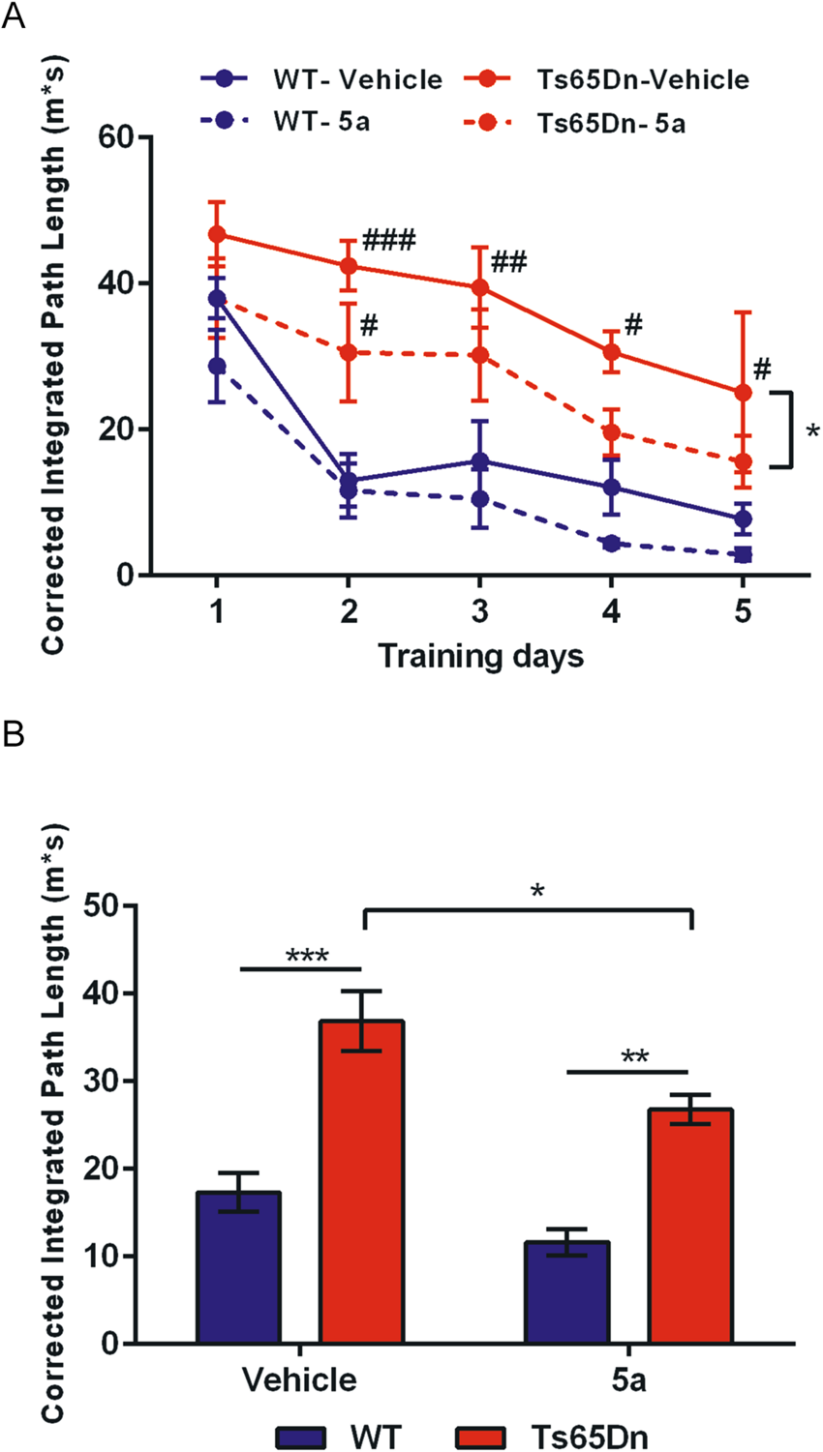
Mice were trained in a standard spatial learning protocol (MWM paradigm). (**A**) All mice showed a gradual improvement across training sessions. As expected, Ts65Dn mice showed a behavioral impairment (increased path lengths to reach the goal location as compared to WT mice). This deficit was partly corrected following treatment with **5a**. (**B**) The same differences between groups were observed using pooled data over the 5 training sessions. Overall comparisons between groups: *p < 0.05; **p < .01; ***p < 0.001. Different from control (WT-Vehicle group): ^#^p < 0.05; ^##^p < 0.01; ^###^p < 0.001.

Ts65Dn mice, the best-characterized and most widely used mouse model of DS, are trisomic for a segment of chromosome 16 extending between genes Mrp139 and Znf295 and containing approximately 92 orthologues of human chromosome 21 genes^42^ including the *Dyrk1a* gene. Using a standard Morris Water Maze (MWM) paradigm, spatial learning was assessed in Ts65Dn mice and euploid WT littermates treated either with compound **5a** (20 mg/Kg) or with vehicle (vehicle solution) every day for the two weeks preceding behavioral testing and also during the MWM protocol (mice being injected 30 min before each daily session). Mice were trained for a total of 5 days to learn a fixed spatial goal location in the maze. Figure 8 shows the learning skills of the mice assessed by their corrected integrated path length (CIPL) to reach the platform. In this paradigm, learning occurred when mice succeeded in decreasing their traveled distances after repeated training trials.

ANOVA on CIPL with two main factors (Genotype and Treatment) and repeated measures (Training days) indicated a significant effect of repetition (F(4,52) = 16.69; p < 0.0001) underscoring that mice gradually improved their performance across training sessions. A main effect of the Genotype factor was concurrently observed (F(1,13) = 64.25; p < 0.0001) due to poorer learning performance of Ts65Dn mice as compared to WT littermates. ANOVA also underscored an effect of the Treatment factor (p < 0.005) that was explained by an overall increase of performance (i.e., decrease of path lengths) in **5a**-treated animals. Importantly posthoc analysis (Bonferroni tests) confirmed a promnesiant effect of **5a** in Ts65Dn (p < 0.05, see Fig. 8A) but not in WT mice (p > 0.44). Day-by-day analysis indicated that while Ts65Dn-vehicle mice were constantly impaired when compared to WT-vehicle mice, Ts65Dn-**5a** mice displayed only an initial delay in learning but then rapidly recovered a level of performance not statistically different from that of WT-vehicle littermates. The rescuing effect of **5a** in the trisomic mice, although significant, was partial as a Genotype effect was still observed in the drug condition (WT > Ts65Dn, posthoc test, p < 0.005). A global analysis of performance with all training sessions pooled confirmed that Ts65Dn-vehicle mice were impaired in comparison to WT littermates (p < 0.001; Fig. 8B). A significant improvement of performance was observed in Ts65Dn mice following treatment with **5a** (p < 0.05) but these mice remained impaired in comparison to WT-**5a** treated animals (p < 0.01).

Subsequently, the percentage of thigmotaxis, i.e. the propensity to swim close to the pool walls, was analyzed. Thigmotaxis refers to an unadapted navigational strategy and hence is considered as a complementary index to assess learning failures in the MWM paradigm. ANOVA with two factors (Genotype and Treatment) revealed a strong increase of thigmotaxis in Ts65Dn mice as compared to WT mice (F(1,13) = 50.1; p < 0.0001) that paralleled their spatial learning impairment (see above). Interestingly, treatment with **5a** induced an overall reduction of thigmotaxis (F(1,13) = 7.8; p < 0.025), observed in WT mice (−6.5% between vehicle and **5a** conditions) which was even more pronounced in Ts65Dn mice (−12.9%). The decrease of thigmotaxis in the trisomic animals might partly be explained by their better learning proficiency under the drug condition (see above).

Finally, analysis of swim speeds underscored differences between genotypes (ANOVA) with two main factors (Genotype and Treatment) and repeated measures (Training days); F(3,65) = 6.493; p < 0.001) with untreated Ts65Dn mice swimming at lower speeds when compared to the three other groups (Bonferroni posthoc tests, all ps < 0.005). Treatment with **5a** restored normal swim speed in Ts65Dn mice (comparison with **5a**-treated WT mice: p > 0.99).

## Conclusions

The cognitive deficiencies observed in DS subjects have been linked to the over-production of cerebral DYRK1A kinase due to the extra copy of chromosome 21. Inhibition of this enzyme would seem, then, to be a viable option for the treatment of learning and memory problems encountered in DS. We have now developed a new family of potent DYRK1A inhibitors, F-DANDYs, which are fluorinated analogues of our previously described DANDY inhibitors. Our preliminary structure-activity relationship study has indicated that replacing one or two of the phenolic OH groups of the DANDY derivatives by fluoride led to some loss of activity though IC_50_s nevertheless remained in the nanomolar range (e.g., compounds **5a**, IC_50_ = 20.7 nM and **5g**, IC_50_ = 9.3 nM). Moreover, as with the DANDY derivatives, methylation of the phenolic alcohol functions led to an important loss of inhibitory potency (e.g., compounds **4a**, IC_50_ = 1.43 µM). These results encouraged us to conduct *in vivo* studies on one of these F-DANDY derivatives, compound **5a**. We first demonstrated, using tandem LC-MS/MS, that **5a** administered i.p. to WT mice at a dose of 20 mg/kg was detectable in plasma for up to one hour at a level of approximately 100 to 150 pg/µL with a slow decrease over 2.5 hours to approximately 50 pg/µL. Using a similar mass spectral technique, compound **5a** was then shown to enter the brain of both WT and Ts65Dn mice, the latter being a commonly utilized model for DS. Spatial learning evaluation of WT and Ts65Dn mice, treated with **5a** (20 mg/kg i.p.), pointed to a significant improvement in performance in the Ts65Dn mice but not the WT mice, providing proof-of-concept as to the working hypothesis that inhibition of DYRK1A by compound **5a** leads to improvement in cognitive abilities of Ts65Dn mice. Only partial recovery of performance of Ts65Dn was observed but it is expected that a more robust effect could be obtained by prenatal treatment with **5a**^43^. This study is the first demonstration of a therapeutic effect of a competitive DYRK1A inhibitor in mice modeling DS. Supporting our data, a recent report underscored similar protective effect of DYRK1A inhibition in an animal model of Alzheimer’s disease^31^, a pathology that shares many links with DS. In summary, we have demonstrated that compound **5a** is a potent, non-toxic inhibitor of DYRK1A whose metabolic stability in plasma and ability to cross the blood-brain barrier translates into cognitive rescuing effects in a mouse model of DS. While further *in vivo* studies will be required, compound **5a** thus represents a potentially viable drug candidate for the treatment of cognitive deficits associated with DS and associated pathologies.

## Methods

### Synthesis of inhibitors

F-DANDY derivatives were prepared using standard synthetic procedures as described in the Supplementary Information.

### Expression and purification of the recombinant DYRK1A catalytic domain (DYRK1A-ΔC)

The cDNA coding for the catalytic domain (residues 1-502) of rat DYRK1A (99.6% amino acid identity with human DYRK1A) was a kind gift of Prof. W. Becker (Aachen University, Germany). This cDNA was subcloned into pET28 plasmid and used to produce recombinant 6xHis-tagged DYRK1A catalytic domain (DYRK1A-ΔC). The pET28-DYRK1A-ΔC plasmid was transformed into *E. coli* BL21(DE3) cells for production and purification of the protein. Briefly, transformed bacterial cells were grown at 37 °C for 4 h in the presence of 0.5 mM isopropyl β-D-1-thiogalactopyranoside and further grown at 4 °C overnight. Cells were harvested by centrifugation and resuspended in phosphate buffered saline buffer (PBS) supplemented with protease inhibitors, 1 mg/mL lysozyme and 0.1% Triton X-100. After 30 min incubation at 4 °C, the lysate was subjected to sonication on ice and pelleted (12000 × g, 30 min). The supernatant was incubated with His-select Nickel resin (Sigma) for 2 h at 4 °C. The resin was poured into a column and washed with Tris-HCl 20 mM, pH 7.5, 10 mM imidazole. Proteins were eluted in Tris-HCl 20 mM, pH 7.5, 300 mM imidazole. Proteins were reduced with 10 mM dithiothreitol (DTT) and dialyzed overnight against Tris-HCl 20 mM, pH 7.5. Proteins were quantified with Bradford’s reagent (Bio-rad). Purity was assessed by SDS-PAGE. Proteins were kept at −80 °C.

### DYRK1A inhibition assays

- *In vitro* measurement of DYRK1A kinase activity was carried out using a UFLC-based approach in combination with a fluorescent peptide substrate of DYRK1A. The fluorescent peptide substrate was derived from the sequence of the human transcription factor FKHR that is known to be a physiological substrate of DYRK1A^37^. The peptide substrate, coupled to fluorescein by its *N*-terminal amino acid, had the following sequence: KISGRLSPIMTEQ (the serine residue that is phosphorylated by DYRK1A is underlined). Purified recombinant rat DYRK1A catalytic domain (DYRK1A-ΔC) was used in all enzymatic assays as previously described^37^. Activity assays were performed in 96-well plates, in a total volume of 50 μL consisting of kinase buffer (Tris-HCl 50 mM, pH 7.5, 10 mM DTT, 5 mM MgCl_2_), peptide substrates (ranging from 5 to 60 μM) and 20 ng of purified DYRK1A-ΔC. The reaction was initiated by addition of ATP (ranging from 50 to 800 μM) and the mixture was incubated for 30 min at 37 °C. Reactions were stopped by addition of 50 μL of 15% HClO_4_ (v/v) and 20 μL of the mixture was analyzed by UFLC (Shimadzu) on a C8 reverse-phase column (Ascentis). Mobile phases used consisted of 0.12% trifluoroacetic acid (TFA) (solvent A) and acetonitrile in 0.12% TFA. Phosphorylated and unphosphorylated peptides were separated by isocratic flow (85% solvent A/15% solvent B) at a flow rate of 1.5 mL/min. The peptides were monitored by fluorimetry (excitation at 485 nm, emission at 530 nm) and quantified by integration of the peak absorbance area. A calibration curve established with different known concentrations of peptides was used for quantification. Initial velocities and kinetic parameters were determined by fitting the data iteratively to the following equation: V_i_ = V_max_ [S_A_][S_B_]/(*K*_A_*K*_mB_ + *K*_mB_[S_A_] + *K*_mA_[S_B_] + [S_A_][S_B_]) using the Prism 5 (GraphPad) program, where V_i_ is the initial velocity, V_m_ is the maximal velocity, [S_A_] and [S_B_] are the substrate concentrations, K_A_ is the dissociation constant of the enzyme for S_A_ while *K*_mA_ and *K*_mB_ are the Michaelis-Menten constants for S_A_ and S_B_^44,45^.
  For inhibition studies, the compounds were added at different concentrations to the well prior to addition of ATP. Determination of the mode of inhibition (competitive/non-competitive) of the compounds and inhibition constant (K_i_) values were obtained by carrying out assays with different concentrations of inhibitors and fitting the data to the following equations: V_i_ = V_max_ [S]/(*K*_m_ (1 + [I]/*K*_I_) + [S]) and V_i_ = (V_max_/(1 + [I]/*K*_I_) [S])/(*K*_m_ + [S]) for competitive and non-competitive inhibition, respectively^45^.
- *In cellulo* studies were performed by Cell Assay Innovations (Beverly, MA) using ClariCELL™ technology (www.cellassayinnov.com). HEK293 human embryonic kidney cells were transiently co-transfected with plasmid sequence verified vectors encoding full-length human DYRK1A and Tau and dispensed into multi-well plates. Cells were dispersed and incubated for 2 h in the presence of harmine, compounds **5a** and **5g** at various concentrations. Harmine was used as a control. The cells were lysed and an ELISA was performed by capturing the phosphorylated Tau substrate and detecting phosphorylation levels using an antibody to phosphor-TauT212. DYRK1A-dependent kinase activity in these assays was validated using a kinase-deficient DYRK1A[K188R].

### Cell culture and proliferation assay

Cancer cell lines were obtained from the American Type Culture Collection (Rockville, MD, USA) and were cultured according to the supplier’s instructions. Briefly, human KB epidermal carcinoma cells were grown in Dulbecco minimal essential medium (DMEM) containing 4.5 g/L glucose supplemented with 10% fetal calf serum (FCS) and 1% glutamine, 100 UI penicillin, 100 µg/mL streptomycin and 1.5 µg/mL fungizone and maintained at 37 °C in a humidified atmosphere containing 5% CO_2_. Cell viability was assessed using Promega CellTiter-Blue TM reagent according to the manufacturer’s instructions. Cells were seeded in 96-well plates (5 × 10^3^ cells/well) containing 50 mL growth medium. After 24 h of culture, the cells were supplemented with 50 mL of the studied compound dissolved in DMSO (less than 0.1% in each preparation). After 72 h of incubation, 20 mL of resazurin was added for 2 h before recording fluorescence (λ_ex_ = 560 nm, λ_em_ = 590 nm) using a Victor microtiter plate fluorimeter (Perkin-Elmer, USA). Results are shown in Table 1.

### Kinase profiling

Kinase profiling was performed by Eurofins-CEREP, Le Bois l’Evêque, France. Compounds were tested at 5.10^−8^ M except for harmine which was tested at 10^−6^ M. All experiments were performed in duplicate and the values reported are the mean values.

### Liquid chromatography/tandem mass spectrometry of compound 5a

All plasma and brain homogenate extracts were analyzed by an ultra-high performance liquid chromatography (UHPLC) system coupled to a triple-quadrupole mass spectrometer (Dionex Ultimate 3000 RSLC system coupled to a TSQ Vantage EMR, Thermo Scientific, Les Ulis, France). UHPLC separation was performed using a C18 column (HSS T3, 2.1 mm × 150 mm, 1.7 μm, Waters, Guyancourt, France) equipped with a guard column (HSS T3, 2.1 mm × 5 mm, 1.7 μm, Waters, Guyancourt, France) and maintained at 40 °C. The mobile phases were water, 0.1% formic acid (A) and acetonitrile (B). Compound **5a** and the internal standard were eluted at a flow rate of 0.5 mL/min, with first 20% B for 4 min then from 20% B to 100% B in 6 min. A washing step in acetonitrile/isopropanol was added to limit carryover problems. Injection volume was 4 µL and the autosampler was maintained at 6 °C. Ionization was performed with a heated electrospray ionization source in positive mode. The source parameters were set as follows: spray voltage 3350 V, vaporizer temperature 250 °C, sheath gas (N_2_) pressure 55 psi, auxiliary gas pressure (N_2_) 25 psi, capillary temperature 350 °C. Analysis was performed under the Selected Reaction Monitoring (SRM) mode using several transitions for each compound at optimized collision energies (see Table SI2 and Figure SI1). Ion ratios were used to confirm the identity of compound **5a** in plasma and brain samples. Data acquisition was performed using ThermoScientific Xcalibur 2.1 software system. All samples were analyzed in triplicate.

**Figure 9:**
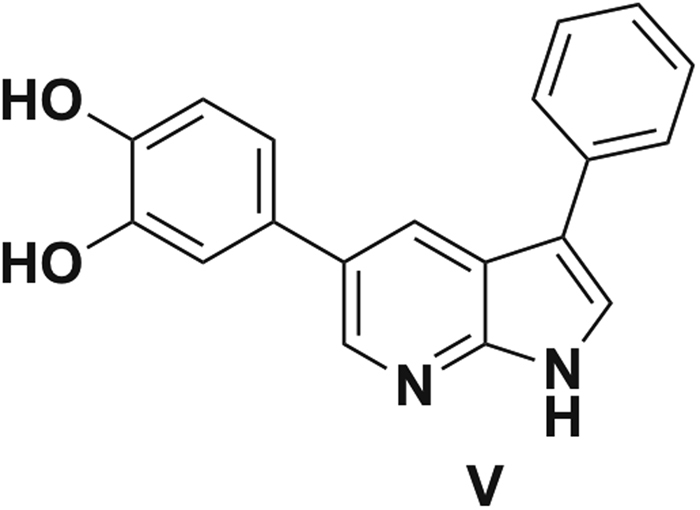
Structure of internal standard compound **V**.

Two compounds, **V**^34^ (Fig. 9) and **5d**, were tested as internal standard (IS). Compound **V** was found to be the most suitable to correct for matrix effects. Calibration curve points and quality control (QC) points were realized in spiked plasma or brain homogenate samples (Figure SI2). Spiking of compound **5a** and IS was realized before extraction to correct for the extraction recovery. In plasma samples, linear curves were obtained from 1.6 to 320.3 pg/μL of compound **5a** with within-run trueness in the 85–125% range and within-run precision better than 15% for all calibration points (r^2^ > 0.995). Samples with concentrations above the upper calibration point were diluted to be in the calibration range. Limit of quantification (LOQ) was determined as the lowest point of the calibration curve at 1.6 pg/μL. QC samples were prepared at 14.5 and 58 pg/μL to validate the between-run precision (n = 3, RSD of 13% and 5%, respectively), between-run trueness was 95 and 88% respectively. In brain homogenate samples, linear curves were obtained from 1.6 to 80 pg/mg of compound **5a** with within-run trueness in the 85–125% range and within-run precision better than 15% (r^2^ > 0.990). Limit of quantification (LOQ) was determined as the lowest point of the calibration curve at 1.6 pg/mg. QC samples were prepared at 3.2 and 22.4 pg/mg to validate the between-run precision (n = 3, RSD of 8% and 2% respectively), between-run trueness was 129 and 93% respectively.

### Animals

Male Ts65Dn (n = 8) mice and euploid wild-type (WT) littermates (n = 10) were purchased from the Jackson Laboratory (Bar Harbor, Maine, USA). As the recessive retinal degeneration 1 mutation Pde6brd1 in the original Ts65Dn mice causes blindness in homozygotes, we used an alternative strain (SN 5252) that is wild-type for Pde6b, thereby preventing retinal degeneration. Housing conditions comprised a standard cage (air-controlled system) with *ad libitum* food and water and a 12 h light/dark cycle. Mice received at least one week of habituation to the animal facility, followed by three days of handling (three minutes per mouse per day). Behavioral experiments were conducted following a two-week treatment with compound **5a** (20 mg/kg) or vehicle by i.p. injections. Treatment was continued during the MWM protocol (mice being injected 30 min before each daily session).

All experiments were conducted in compliance with the ethical standards and animal welfare regulations of the French (Ministry of Agriculture) and European authorities (European Communities Council Directive of 24 November 1986). *In vivo* protocols used in the present study were also approved by our local ethics committee (Charles Darwin Committee). MC Potier has authorization n° A-75-2138 and B. Delatour has authorization n° A-75e-1741 from the Direction Départementale de la Protection des Populations de Paris (Service Protection et Santé Animales, Environnement) to perform experiments on vertebrates.

### Formulation of compound 5a

Compound **5a** in powder form was dissolved for injection in a mixture of DMSO, Cremophor EL (Sigma-Aldrich Chemie, Steinheim, Germany) and Proamp® sterile water for parenteral use (Laboratoire Aguettant, Lyon, France) (10:15:75) with a final concentration of 2.4 mg/mL^46^. Vehicle consisted of an identical composition without compound **5a**. Mice received i.p. injections of compound **5a** (20 mg/kg) or vehicle.

### Bioavailability of compound 5a in plasma

To determine the bioavailability of compound **5a** after i.p. injections, 12 WT mice were injected with 20 mg/kg of compound **5a** and sacrificed at 12 successive time points. After decapitation, blood was sampled and transferred into BD Microtainer® SST tubes (Becton, Dickinson and Company, Plymouth, UK). Plasma was obtained according to the manufacturer’s protocol and stored at −80 °C.

Using an optimized Ultra Performance Liquid Chromatography with tandem mass spectrometry (UPLC-MS/MS), compound **5a** was subsequently quantified in plasma. In short, proteins were precipitated using liquid/liquid extraction with ethyl acetate and supernatant was transferred into 40 µL DMSO/water/acetonitrile (12/44/44). Thereupon, UPLC separation was performed using a C18 column (HSS T3, Waters, Guyancourt, France) and a gradient mobile phase (acetonitrile and water with 0.1% formic acid), followed by triple quadrupole MS (TSQ Vantage, Thermo Scientific) with electrospray ionization. All samples were analyzed in triplicate.

### Effect of compound 5a on behavioral deficit of Ts65Dn mice in the Morris water maze

To analyze the effect of compound **5a** on spatial learning, a Morris Water Maze (MWM) protocol was used as previously described^46^. A white pool with a diameter of 150 cm was filled with water made opaque with non-toxic white paint (Acusol OP301, Rohm and Haas Company, Landskrone, Sweden) and kept at a constant temperature (20–21.5 °C). A 9-cm diameter platform was placed in the south-east (SE) quadrant of the pool, 1 cm under the water surface. The light intensity in the experimental room was kept constant at 70 Lux. Mice were habituated to the room for at least 30 min before starting each daily session. The acquisition phase comprised 5 days of training, four trials per day with an inter-trial interval of at least 20 min to minimize motor fatigue. The order of release positions in the pool was pseudo-randomly at the four cardinal points and changed every day to maximize spatial allocentric training and formation of a “cognitive map”. Each trial ended as soon as the mouse found the platform or after a maximum of 90 s, after which the animal was gently manually guided to the platform. Mice were left on the platform for 20 s before they were gently dried with a towel and individually placed in a cage with paper towels for further drying. The animals returned to their homecage after 5 min.

ANY-maze Video Tracking System (Stoelting, USA) was used to record all trials. Calculated measures included 1) thigmotaxis (percent distance traveled in the 10 cm wide peripheral annulus of the pool) indicative of unsuccessful spatial strategy to locate the platform and 2) the Corrected Integrated Pathway Length (CIPL) an unbiased measure of distance-to-platform travelled taking into account the animal’s speed and the initial distance from the goal for each starting position^47^.

### Quantification of compound 5a in mouse brain

Immediately following last day of training in the MWM, mice were sacrificed by decapitation for maximal blood collection and the brain was immediately extracted from the skull, the cerebellum removed and the hemispheres separated and frozen at −80 °C. Plasma was obtained as described above. For UHPLC-MS/MS quantification of compound **5a** in the brain, one hemisphere of each mouse brain was homogenized in methanol (J.T. Baker analyzed LC-MC reagent, Avantor Performance Materials BV, Deventer, The Netherlands), 1 mL per 100 mg brain. Subsequently, the IS was added to 400 µL of homogenate which was then centrifuged (12 min, 14000 × g, 4 °C). The supernatant was evaporated and transferred into 40 µL acetonitrile/methanol/water (2/2/1). After centrifugation (12 min, 14000 × g, 4 °C), 4 µL of supernatant was used for UHPLC-MS/MS analysis.

## Electronic supplementary material

Supplementary Information
