# Supplementary material for: DYRK1A inhibition and cognitive rescue in a Down syndrome mouse model are induced by new fluoro-DANDY derivatives
Source: Sci Rep. 2018 Feb 12;8:2859. doi: 10.1038/s41598-018-20984-z (PMC5809559; doi:10.1038/s41598-018-20984-z)
Supplement: Supplementary file 1 — Supplementary Information [file 41598_2018_20984_MOESM1_ESM.pdf]

Supplementary Information for:

DYRK1A inhibition and cognitive rescue in a Down syndrome mouse model are induced by new fluoro-DANDY derivatives

Fernanda Neumann[1], Stéphanie Gourdain[1], Christelle Albac[2], Alain D. Dekker[2,3], Linh Chi Bui[4], Julien Dairou[5], Isabelle Schmitz-Afonso[1], Nathalie Hue[1], Fernando Rodrigues-Lima[4], Jean M. Delabar[2], Marie-Claude Potier[2], Jean-Pierre Le Caër[1], David Touboul[1], Benoît Delatour[2], Kevin Cariou[1], and Robert H. Dodd[1]

[1] Institut de Chimie des Substances Naturelles, CNRS UPR 2301, Université Paris-Sud, Université Paris-Saclay, Avenue de la Terrasse, 91198 Gif-sur-Yvette, France. [2] Sorbonne Universités, Université Pierre et Marie Curie (UPMC), Université Paris 06, Institut National de la Santé et de la Recherche Médicale (INSERM) and Centre National de la Recherche Scientifique (CNRS) Unités de Recherche U75, U1127, U7225, and Institut du Cerveau et de la Moelle Epinière (ICM), 75013 Paris, France. [3] Department of Neurology, University of Groningen, University Medical Center Groningen (UMCG), Hanzeplein 1, 9713 GZ, Groningen, The Netherlands. [4] Université Paris Diderot, Sorbonne Paris Cité, Unité BFA, CNRS UMR 8251, 75013 Paris, France. [5] UMR 8601 CNRS, Laboratoire de Chimie et Biochimie Pharmacologiques et Toxicologiques, Université Paris Descartes-Sorbonne Paris Cité, 75270 Paris, France.

Supplementary information contains:

1. Synthesis of the fluoro-DANDY inhibitors S3

1.1 General Remarks S3
1.2 General Procedure and Analytical Data for 2a-2d and 3a-3e. S4
1.3 General Procedure and Analytical Data for 4a-4f. S11
1.4 General Procedure and Analytical Data for 5a-5g S16

2. NMR Spectra of 2a-2d, 3a-3e, 4a-4f and 5a-5g S21

3. Supplementary Figures S45

Figure SI1: MS/MS spectrum of compound 5a S45
Figure SI2: Extracted Ion Chromatograms S46

4. Supplementary Table S47

Table SI2: Optimized SRM transitions for compound 5a and IS S47

1. Synthesis of the fluoro-DANDY inhibitors

1.1 General Remarks.

Melting points were measured in capillary tubes on a Büchi B-540 apparatus and are uncorrected. Infrared spectra were recorded on a Perkin Elmer Spectrum BX FT-IR spectrometer. Proton (^{1}H) and carbon (^{13}C) NMR spectra were recorded on Bruker spectrometers: Avance 300 MHz (QNP - ^{13}C, ^{31}P, ^{19}F - probe or Dual ^{13}C probe) and Avance 500 MHz (BB0 - ATM probe or BBI - ATM probe). Carbon NMR (^{13}C) spectra were recorded at 125 or 75 MHz using a broadband decoupled mode with the multiplicities obtained using a DEPT sequence. NMR experiments were carried out in deuterochloroform ($CDCl_3$), for which chemical shifts (δ) are reported in parts per million (ppm) with reference to $CDCl_3$ (^{1}H: 7.26; ^{13}C: 77.16) and deuteromethanol (CD_3OD), for which chemical shifts (δ) are reported in parts per million (ppm) with reference to CD_3OD (^{1}H: 3.34; ^{13}C: 49.86). The following abbreviations are used for the proton spectra multiplicities: s: singlet, brs: broad singlet, d: doublet, t: triplet, q: quartet, m: multiplet, br: broad. Coupling constants (*J*) are reported in Hertz (Hz). Mass spectra were obtained either with an LCT (Micromass) instrument using electrospray ionization (ES) or from a Time of Flight analyzer (ESI-MS) for the high resolution mass spectra (HRMS). Thin-layer chromatography was performed on silica gel 60 F254 on aluminum plates (Merck) and visualized under a UVP Mineralight UVLS-28 lamp (254 nm) and with 4-anisaldehyde and phosphomolybdic acid stains in ethanol. Flash chromatography was conducted on Merck silica gel 60 (40-63 µm) at medium pressure (300 mbar). All reagents were obtained from commercial suppliers unless otherwise stated.

1.2 General Procedure for the Preparation of the 3-Aryl-7-azaindole Derivatives 2a-2d and 3,5-Diaryl-7-azaindoles 3a-3e.

To solution of 5-bromo-3-iodo-1-(phenylsulfonyl)-1*H*-pyrrolo[2,3-*b*]pyridine (**1**, 1 equiv)[32] or of 5-bromo-3-aryl-1-(phenylsulfonyl)-1*H*-pyrrolo[2,3-*b*]pyridine (**2a**-**2d**, 1 equiv) in a degassed mixture of toluene and ethanol (3:1, 0.03 M) were added the appropriately substituted phenylboronic acid (1 equiv), K_2CO_3 (2M solution in water, 3 equiv) and $Pd(PPh_3)_4$ (1.5 mol%). The reaction mixture was heated at 110 °C for 5 h under argon then cooled to room temperature and concentrated under vacuum. The residue was partitioned between water and CH_2Cl_2 and the aqueous layer was extracted with CH_2Cl_2 (2x). The organic extracts were combined, dried over $MgSO_4$ and the solvents were removed under vacuum. The residue was purified as described below to give the following compounds:

3-(4-Fluorophenyl)-5-bromo-1-(phenylsulfonyl)-1H-pyrrolo[2,3-b]pyridine (***2a***).

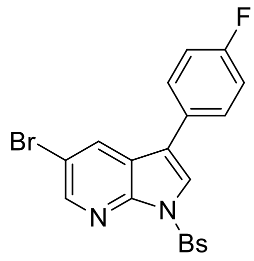

Prepared as described above using **1** (200 mg, 0.43 mmol) and 4-fluorophenylboronic acid (60 mg, 0.43 mmol) and purified by flash chromatography on silica gel (6:4 heptane/CH_2Cl_2). White solid (120 mg, 65%). ^{1}H NMR (300 MHz, $CDCl_3$) δ 7.16-7.22 (m, 2H), 7.50-7.65 (m, 5H), 7.85 (s, 1H), 8.16 (d, J = 2.1 Hz, 1H), 8.21-8.24 (m, 2H), 8.50 (d, J = 2.1 Hz, 1H). ^{13}C NMR (75 MHz, $CDCl_3$) δ 115.6, 116.1(d, J = 20.0 Hz, 2C), 118.8, 123.0, 123.8, 127.9 (d, J = 3.3 Hz, 1C), 128.1, 129.0 (d, J = 8.2 Hz, 2C), 129.1, 130.9, 134.4, 137.9, 145.7, 145.8, 160.8 (d, J = 248.1 Hz, 1C). IR (cm^{-1}) ν 3146, 3077, 2922, 1379, 1165. HRMS (ES+) *m/z* calcd for $C_{19}H_{13}FN_2O_2S^{79}Br$ $[M + H]^+$ 430.9865, found 430.9845; *m/z* calcd for $C_{19}H_{13}FN_2O_2S^{81}Br$ $[M + H]^+$ 432.9845, found 432.9831.

3-(3,4-Difluorophenyl)-5-bromo-1-(phenylsulfonyl)-1H-pyrrolo[2,3-b]pyridine (***2b***).

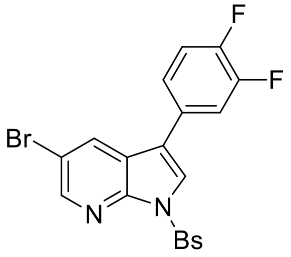

Prepared as described above using **1** (300 mg, 0.65 mmol) and 3,4-difluorophenylboronic acid (50 mg, 0.32 mmol) and purified by trituration in CH_3OH/CH_2Cl_2. White solid (175 mg, 60%). ^{1}H NMR (300 MHz, $CDCl_3$) δ 7.28-7.39 (m, 3H), 7.51-7.57 (m, 2H), 7.61-7.66 (m, 1H), 7.87 (s, 1H), 8.15 (d, J = 2.1 Hz, 1H), 8.22-8.26 (m, 2H), 8.52 (d, J = 2.1 Hz, 1H). ^{13}C NMR (75 MHz, $CDCl_3$) δ 115.8, 116.3 (d, J = 18.1 Hz, 1C), 117.8, 118.1 (d, J = 18.1 Hz, 1C), 122.6, 123.5 (m, 1C), 124.2, 128.2, 128.9 (d, J = 3.8 Hz, 1C), 129.2, 130.7, 134.5, 137.7, 145.6, 146.1, 148.3 (dd, J = 249.7, 12.6 Hz, 1C), 149.0 (dd, J = 249.7, 12.6 Hz, 1C). IR (cm^{-1}) ν 3140, 3069, 1369, 1168. HRMS (ES+) *m/z* calcd for $C_{19}H_{12}F_2N_2O_2S^{79}Br$ $[M + H]^+$ 448.9771, found 448.9782; *m/z* calcd for $C_{19}H_{12}F_2N_2O_2S^{81}Br$ $[M + H]^+$ 450.9750 found, 450.9767.

3-(3-Fluoro-4-methoxyphenyl)-5-bromo-1-(phenylsulfonyl)-1H-pyrrolo[2,3-b]pyridine (***2c***).

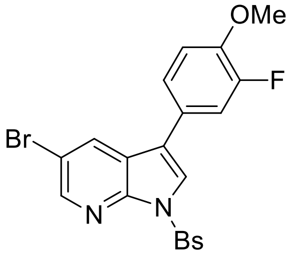

Prepared as described above using **1** (604 mg, 1.31 mmol) and 3-fluoro-4-methoxyphenylboronic acid (111 mg, 0.65 mmol) and purified by flash chromatography on silica gel (CH_2Cl_2). White solid (297 mg, 50%). ^{1}H NMR (300 MHz, $CDCl_3$) δ 3.96 (s, 3H), 7.04-7.10 (m, 1H), 7.26-7.30 (m, 2H), 7.50-7.55 (m, 2H), 7.60-7.65 (m, 1H), 7.83 (s, 1H), 8.17 (d, J = 2.3 Hz, 1H), 8.20-8.23 (m, 2H), 8.50 (d, J = 2.1 Hz, 1H). ^{13}C NMR (75 MHz,

$CDCl_3$) δ 56.4, 114.0 (d, *J* = 2.7 Hz, 1C), 115.0 (d, *J* = 19.2 Hz, 1C), 115.6, 118.5 (d, *J* = 2.2 Hz, 1C), 123.0, 123.3 (d, *J* = 3.8 Hz, 1C), 123.6, 124.8 (d, *J* = 7.1 Hz, 1C), 128.1, 129.2, 130.9, 134.4, 137.8, 145.7 (d, *J* = 15.9 Hz, 1C), 145.9, 147.4, 150.9 (d, *J* = 247.6 Hz, 1C). IR (cm^{-1}) ν 3121, 3037, 2886, 1378, 1161. HRMS (ES+) *m/z* calcd for $C_{20}H_{15}FN_2O_3S^{79}Br$ [M + H]$^+$ 460.9971, found 460.9975; *m/z* calcd for $C_{20}H_{15}FN_2O_3S^{81}Br$ [M + H]$^+$ 462.9950, found 462.9966.

3-(3,4-Dimethoxyphenyl)-5-bromo-1-(phenylsulfonyl)-1H-pyrrolo[2,3-b]pyridine (***2d***).

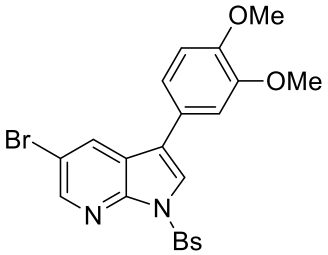

Prepared as described above using **1** (115 mg, 0.25 mmol) and 3,4-dimethoxyphenylboronic acid (45 mg, 0.25 mmol) and purified by flash chromatography on silica gel (95:5 CH_2Cl_2/heptane). White solid (85 mg, 72%). ^{1}H NMR (300 MHz, $CDCl_3$) δ 3.94 (s, 3H), 3.96 (s, 3H), 6.97 (d, *J* = 8.3 Hz, 1H), 7.02 (d, *J* = 2.1 Hz, 1H), 7.09 (dd, *J* = 8.3, 2.1 Hz, 1H), 7.49-7.55 (m, 2H), 7.59-7.64 (m, 1H), 7.84 (s, 1H), 8.18 (d, *J* = 2.1 Hz, 1H), 8.20-8.24 (m, 2H), 8.49 (d, *J* = 2.1 Hz, 1H). ^{13}C NMR (75 MHz, $CDCl_3$) δ 56.0, 56.1, 110.7, 111.7, 115.5, 119.8, 120.0, 123.3, 124.5, 128.1, 129.1, 131.1, 134.3, 138.0, 145.7, 149.1, 149.6. IR (cm^{-1}) ν 3127, 3000, 2928, 2853, 1384, 1148. HRMS (ES+) *m/z* calcd for $C_{21}H_{18}N_2O_4S^{79}Br$ [M + H]$^+$ 473.0171, found 473.0150; *m/z* calcd for $C_{21}H_{18}N_2O_4S^{81}Br$ [M + H]$^+$ 475.0150, found 475.0135.

3-(4-Fluorophenyl)-5-(3,4-dimethoxyphenyl)-1-(phenylsulfonyl)-1H-pyrrolo[2,3-b]pyridine (***3a***).

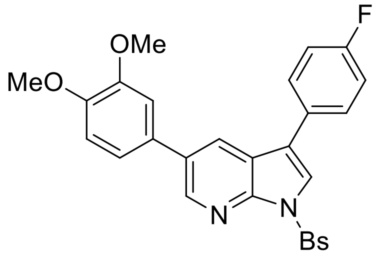

Prepared as described above using **2a** (150 mg, 0.35 mmol) and 3,4-dimethoxyphenylboronic acid (64 mg, 0.35 mmol) and purified by flash chromatography on silica gel (CH_2Cl_2). White solid (159 mg, 92%). ^{1}H NMR (300 MHz, $CDCl_3$) δ 3.93 (s, 3H), 3.94 (s, 3H), 6.96 (d, J = 8.5 Hz, 1H), 7.05 (d, J = 1.9 Hz, 1H), 7.09 (dd, J = 8.3, 2.1 Hz, 1H), 7.17-7.22 (m, 2H), 7.50-7.64 (m, 5H), 7.87 (s, 1H), 8.12 (d, J = 2.1 Hz, 1H), 8.27-8.30 (m, 2H), 8.67 (d, J = 2.1 Hz, 1H). ^{13}C NMR (75 MHz, $CDCl_3$) δ 56.0, 56.1, 110.8, 111.7, 116.0 (d, J = 22.0 Hz, 2C), 119.6, 120.0, 121.5, 123.1, 126.6, 128.1, 128.6 (d, J = 3.3 Hz, 1C), 129.1, 129.1 (d, J = 8.2 Hz, 2C), 131.2, 133.1, 134.1, 138.3, 144.4, 146.5, 149.1, 149.4, 160.8 (d, J = 248.1 Hz, 1C). IR (cm^{-1}) ν 3131, 3059, 2935, 1381, 1154. HRMS (ES+) *m/z* calcd for $C_{27}H_{22}FN_2O_4S$ [M + H]$^+$ 489.1284, found 489.1290.

*3-(4-Fluorophenyl)-5-(2,4-dimethoxyphenyl)-1-(phenylsulfonyl)-1H-pyrrolo[2,3-b]pyridine (**3b**).*

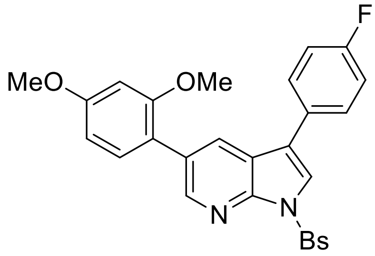

Prepared as described above using **2a** (108 mg, 0.25 mmol) and 3,4-dimethoxyphenylboronic acid (45 mg, 0.25 mmol) and purified by flash chromatography on silica gel (8:2 CH_2Cl_2/heptane). White solid (92 mg, 75%). ^{1}H NMR (300 MHz, $CDCl_3$) δ 3.78 (s, 3H), 3.87 (s, 3H), 6.58-6.61 (m, 2H), 7.14-7.24 (m, 3H), 7.50-7.63 (m, 5H), 7.84 (s, 1H), 8.13 (d, J =

1.7 Hz, 1H), 8.27 (d, J = 7.4 Hz, 2H), 8.60 (d, J = 1.7 Hz, 1H). ^{13}C NMR (75 MHz, $CDCl_3$) δ 55.5, 55.6, 99.0, 104.9, 115.9 (d, J = 21.4 Hz, 2C), 119.6, 120.1, 121.0, 122.5, 128.1, 129.0 (m, 2C), 130.0, 131.4, 134.0, 138.4, 146.2, 146.4, 157.5, 160.9 (d, J = 230.5 Hz, 1C). IR (cm^{-1}) ν 3140, 3060, 2921, 1371, 1155. HRMS (ES+) *m/z* calcd for $C_{27}H_{22}FN_2O_4S$ $[M + H]^+$ 489.1284, found 489.1288.

*3-(3,4-Difluorophenyl)-5-(3,4-dimethoxyphenyl)-1-(phenylsulfonyl)-1H-pyrrolo[2,3-b]pyridine (**3c**).*

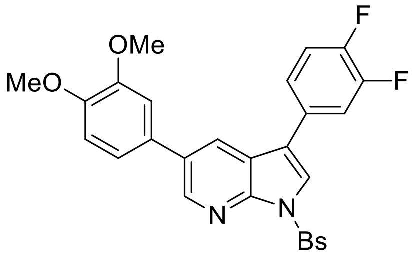

Prepared as described above using **2b** (100 mg, 0.22 mmol) and 3,4-dimethoxyphenylboronic acid (40 mg, 0.22 mmol) and purified by flash chromatography on silica gel (CH_2Cl_2). White solid (97 mg, 87%). ^{1}H NMR (300 MHz, $CDCl_3$) δ 3.94 (s, 3H), 3.95 (s, 3H), 7.00 (d, J = 8.3 Hz, 1H), 7.04 (d, J = 2.1 Hz, 1H), 7.10 (dd, J = 8.3, 2.1 Hz, 1H), 7.25-7.38 (m, 2H), 7.39-7.46 (m, 1H), 7.51-7.57 (m, 2H), 7.60-7.66 (m, 1H), 7.89 (s, 1H), 8.10 (d, J = 2.1 Hz, 1H), 8.27-8.30 (m, 2H), 8.68 (d, J = 2.1 Hz, 1H). ^{13}C NMR (75 MHz, $CDCl_3$) δ 56.0, 56.1, 110.8, 111.8, 116.3 (d, J = 18.1 Hz, 1C), 118.0 (d, J = 17.0 Hz, 1C), 118.6 (d, J = 2.2 Hz, 1C), 120.0, 121.1, 123.5, 123.5-123.7 (m), 126.4, 128.2, 129.1, 129.6-129.7 (m), 131.0, 133.2, 134.2, 138.2, 144.6, 146.5, 148.2 (dd, J = 249.7, 12.6 Hz, 1C), 149.0 (dd, J = 249.2, 12.6 Hz, 1C), 149.2, 149.5. IR (cm^{-1}) ν 3167, 3030, 2936, 1381, 1170. HRMS (ES+) *m/z* calcd for $C_{27}H_{21}F_2N_2O_4S$ $[M + H]^+$ 507.1190, found 507.1198.

*3-(3,4-Difluorophenyl)-5-(2,4-dimethoxyphenyl)-1-(phenylsulfonyl)-1H-pyrrolo[2,3-b]pyridine (**3d**).*

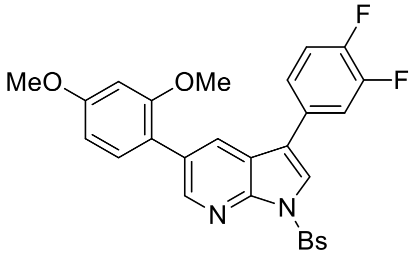

Prepared as described above using **2b** (200 mg, 0.44 mmol) and 2,4-dimethoxyphenylboronic acid (80 mg, 0.44 mmol) and purified by flash chromatography on silica gel (8:2 CH_2Cl_2/heptane). White solid (37 mg, 35%). ^{1}H NMR (300 MHz, $CDCl_3$) δ 3.79 (s, 3H), 3.87 (s, 3H), 6.59-6.62 (m, 2H), 7.21-7.28 (m, 2H), 7.30-7.36 (m, 1H), 7.38-7.45 (m, 1H), 7.50-7.55 (m, 2H), 7.59-7.64 (m, 1H), 7.86 (s, 1H), 8.13 (d, *J* = 2.1 Hz, 1H), 8.27-8.30 (m, 2H), 8.61 (d, *J* = 2.1 Hz, 1H). ^{13}C NMR (75 MHz, $CDCl_3$) δ 55.48, 55.54, 99.0, 104.9, 116.2 (d, *J* = 18.1 Hz, 1C), 117.8 (d, *J* = 17.6 Hz, 1C), 118.6 (d, *J* = 1.6 Hz, 1C), 119.9, 120.6, 123.0, 123.4-123.6 (m), 128.1, 129.0 (d, *J* = 8.2 Hz, 1C), 129.1, 129.8 (d, *J* = 2.2 Hz, 1C), 130.2, 131.4, 134.1, 138.3, 146.1, 146.6, 148.1 (dd, *J* = 249.2, 12.1 Hz, 1C), 148.9 (dd, *J* = 249.2, 12.6 Hz, 1C), 157.5, 161.0. IR (cm^{-1}) *v* 3134, 3060, 2930, 1373, 1160. HRMS (ES+) *m/z* calcd for $C_{27}H_{21}F_2N_2O_4S$ $[M + H]^+$ 507.1190, found 507.1179.

*3-(3-Fluoro-4-methoxyphenyl)-5-(4-benzyloxyphenyl)-1-(phenylsulfonyl)-1H-pyrrolo[2,3-b]pyridine (**3e**).*

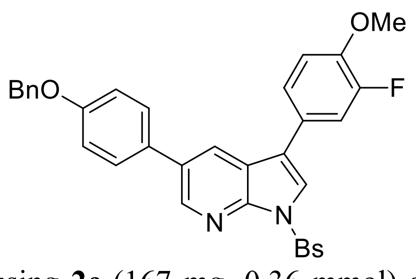

Prepared as described above using **2c** (167 mg, 0.36 mmol) and 4-benzyloxyphenylboronic acid (82 mg, 0.36 mmol) and purified by flash chromatography on silica gel (CH_2Cl_2). White solid (108 mg, 53%). ^{1}H NMR (300 MHz, $CDCl_3$) δ 3.96 (s, 3H), 5.13 (s, 2H), 7.05-7.11 (m,

3H), 7.32-7.55 (m, 11H), 7.58-7.64 (m, 1H), 7.84 (s, 1H), 8.14 (d, *J* = 2.1 Hz, 1H), 8.26-8.29 (m, 2H), 8.67 (d, *J* = 2.1 Hz, 1H). ^{13}C NMR (75 MHz, $CDCl_3$) δ 56.3, 70.1, 113.9 (d, *J* = 2.7 Hz, 1C), 115.1 (d, *J* = 19.2 Hz, 1C), 115.4, 115.9 (d, *J* = 3.8 Hz, 1C), 119.3, 121.4, 122.8, 123.3 (d, *J* = 3.3 Hz, 1C), 125.5 (d, *J* = 7.1 Hz, 1C), 126.5, 127.4, 128.0, 128.5, 128.6, 129.1, 130.9, 132.7, 134.1, 136.7, 138.2, 144.2, 146.5, 147.2 (d, *J* = 11.0 Hz, 1C), 150.9 (d, *J* = 247.0 Hz, 1C), 158.7. IR (cm^{-1}) ν 3033, 2932, 1381, 1232, 1175. HRMS (ES+) *m/z* calcd for $C_{33}H_{26}FN_2O_4S$ $[M + H]^+$ 565.1597, found 565.1594.

*3-(3,4-Dimethoxyphenyl)-5-(3,4-dibenzyloxyphenyl)-1-(phenylsulfonyl)-1H-pyrrolo[2,3-b]pyridine (**3f**).*

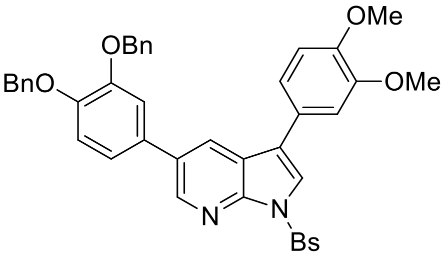

To solution of **2d** (63 mg, 0.13 mmol), 3,4-dibenzyloxyphenylboronic acid pinacol ester[34] (67 mg, 0.16 mmol) and PPh_3 (2 mg, 6 mol%) in degassed dioxane (5 mL) were added potassium acetate (0.4 mL of a 2 M aqueous solution, 0.78 mmol) and $Pd(PPh_3)_2Cl_2$ (3 mg, 3 mol%). The reaction mixture was heated at 100 °C for 12 h under argon then cooled to room temperature and concentrated under vacuum. The residue was partitioned between water and CH_2Cl_2 and the aqueous layer was extracted with CH_2Cl_2 (2x). The organic extracts were combined, dried over $MgSO_4$ and the solvents were removed under vacuum. The residue was purified by flash column chromatography on silica gel (9:1 CH_2Cl_2/heptane) affording compound **3f** as a yellow solid (54 mg, 61%). ^{1}H NMR (300 MHz, $CDCl_3$) δ 3.95 (s, 3H), 3.96 (s, 3H), 5.22 (s, 4H), 6.98-7.03 (m, 1H), 7.06-7.09 (m, 1H), 7.13 (d, *J* = 1.9 Hz, 1H), 7.14 (dd, *J* = 8.3, 1.9 Hz, 1H), 7.33-7.39 (m, 7H), 7.45-7.55 (m, 7H), 7.58-7.63 (m, 1H), 7.84 (s, 1H), 8.08 (d, *J* = 2.1 Hz, 1H), 8.25-8.28 (m, 2H), 8.59 (d, *J* = 2.1 Hz, 1H). ^{13}C NMR (75 MHz, $CDCl_3$) δ 56.0, 56.1, 71.2, 71.5, 110.7, 111.6, 114.6, 115.3, 120.0, 120.5, 120.6, 121.8,

122.6, 125.2, 126.8, 127.2, 127.3, 127.8, 127.9, 128.0, 128.5, 129.1, 131.8, 132.7, 134.1, 136.9, 137.0, 138.3, 144.2, 146.6, 148.9, 149.0, 149.2, 149.5. IR (cm^{-1}) *v* 3063, 3031, 2927, 2854, 1380, 1174. HRMS (ES+) *m/z* calcd for $C_{41}H_{35}N_2O_6S$ $[M + H]^+$ 683.2216, found 683.2224.

1.3 General Procedure for the Preparation of the *N*-Deprotected 3,5-Diaryl-7-azaindoles 4a-4f.

To a solution of the 3,5-diaryl-1-(phenylsulfonyl)-1*H*-pyrrolo[2,3-*b*]pyridine (**3a**-**3f**, 1 equiv) in methanol (0.4 M) was added an aqueous solution of NaOH (2N, 0.5 equiv). The reaction mixture was heated at 80 °C for 2 h, then cooled to room temperature and concentrated under vacuum. The residue was partitioned between water and CH_2Cl_2 and the aqueous layer was extracted with CH_2Cl_2 (2x). The organic extracts were combined, dried over $MgSO_4$ and the solvents were removed under vacuum. The residue was purified as described below to give the following compounds:

3-(4-Fluorophenyl)-5-(3,4-dimethoxyphenyl)-1H-pyrrolo[2,3-b]pyridine (***4a***).

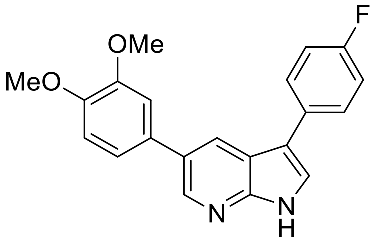

Prepared as described above from **3a** (159 mg, 0.32 mmol) and purified by flash chromatography on silica gel (99:1 CH_2Cl_2/MeOH). Pale yellow solid (84 mg, 75%). ^{1}H NMR (300 MHz, $CDCl_3$) δ 3.96 (s, 3H), 4.00 (s, 3H), 7.00 (d, J = 8.3 Hz, 1H), 7.14-7.21 (m, 4H), 7.56 (s, 1H), 7.61-7.65 (m, 2H), 8.31 (d, J = 1.9 Hz, 1H), 8.59 (d, J = 2.1 Hz, 1H), 11.0 (s, 1H). ^{13}C NMR (75 MHz, $CDCl_3$) δ 56.0, 56.1, 110.9, 111.7, 115.7 (d, J = 21.4 Hz, 2C), 115.8, 118.7, 119.9, 123.0, 126.5, 128.6 (d, J = 7.7 Hz, 2C), 130.3, 130.8 (d, J = 3.3 Hz, 1C), 132.3, 141.9, 148.1, 148.7, 149.4, 160.0 (d, J = 245.4 Hz, 1C). IR (cm^{-1}) ν 3130, 3033, 2904, 1247. HRMS (ES+) *m/z* calcd for $C_{21}H_{18}FN_2O_2$ $[M + H]^+$ 349.1352, found 349.1357.

UPLC R_t = 4.07 min; area 100%.

*3-(4-Fluorophenyl)-5-(2,4-dimethoxyphenyl)-1H-pyrrolo[2,3-b]pyridine (**4b**).*

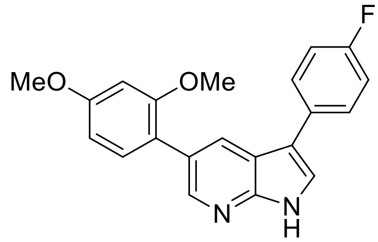

Prepared as described above from **3b** (61 mg, 0.12 mmol) and purified by preparative chromatography on silica gel (9:1 CH_2Cl_2/MeOH). Pale yellow solid (37 mg, 88%). ^{1}H NMR (300 MHz, $CDCl_3$) δ 3.83 (s, 3H), 3.89 (s, 3H), 6.62-6.64 (m, 2H), 7.12-7.18 (m, 2H), 7.29-7.32 (m, 1H), 7.54 (s, 1H), 7.59-7.64 (m, 2H), 8.31 (s, 1H), 8.53 (s, 1H), 11.32 (s, 1H). ^{13}C NMR (75 MHz, $CDCl_3$) δ 55.5, 55.6, 99.1, 104.8, 115.6 (d, J = 21.4 Hz, 2C), 118.5, 121.1, 122.6, 127.0, 128.5, (d, J = 7.7 Hz, 2C), 129.3, 131.0 (d, J = 3.3 Hz, 1C), 131.5, 143.5, 147.4, 157.6, 159.9 (d, J = 244.8 Hz, 1C), 160.6. IR (cm^{-1}) ν 3118, 3011, 2837, 1208. HRMS (ES+) *m/z* calcd for $C_{21}H_{18}FN_2O_2$ $[M + H]^+$ 349.1352, found 349.1356.

UPLC R_t = 4.34 min; area 100%.

*3-(3,4-Difluorophenyl)-5-(3,4-dimethoxyphenyl)-1H-pyrrolo[2,3-b]pyridine (**4c**).*

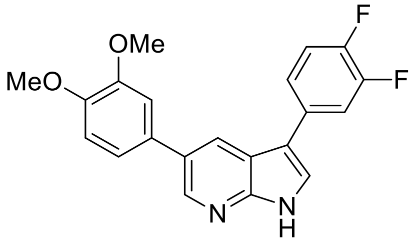

Prepared as described above from **3c** (70 mg, 0.14 mmol) and purified by preparative chromatography on silica gel (96:4 CH_2Cl_2/MeOH). Pale yellow solid (37 mg, 72%). ^{1}H NMR (300 MHz, $CDCl_3$) δ 3.96 (s, 3H), 3.99 (s, 3H), 7.00 (d, J = 8.1 Hz, 1H), 7.13 (d, J = 1.9 Hz, 1H), 7.17 (dd, J = 8.3, 1.7 Hz, 1H), 7.22-7.31 (m, 1H), 7.35-7.40 (m, 1H), 7.42-7.50 (m, 1H), 7.58 (s, 1H), 8.32 (d, J = 1.7 Hz, 1H), 8.59 (d, J = 1.9 Hz, 1H), 11.19 (s, 1H). ^{13}C NMR (75 MHz, $CDCl_3$) δ 56.0, 56.1, 110.9, 111.8, 114.9, 115.7 (d, J = 17.6 Hz, 1C), 117.7 (d, J = 17.0 Hz, 1C), 118.4, 119.9, 122.9-123.0 (m), 123.4, 126.4, 130.6, 131.8-131.9 (m), 132.1, 142.2, 147.4 (dd, J = 247.6, 12.6 Hz, 1C), 148.1, 148.8, 148.9 (dd, J = 247.6, 12.6 Hz,

1C), 149.4. IR (cm^{-1}) ν 3128, 3027, 2965, 1268. HRMS (ES+) *m/z* calcd for $C_{21}H_{17}F_2N_2O_2$ [M + H]$^+$ 367.1258, found 367.1266.

UPLC R_t = 4.24 min; area 100%.

*3-(3,4-Difluorophenyl)-5-(2,4-dimethoxyphenyl)-1H-pyrrolo[2,3-b]pyridine (**4d**).*

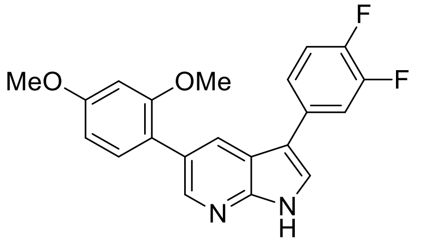

Prepared as described above from **3d** (35 mg, 0.07 mmol) and purified by preparative chromatography on silica gel (9.8:0.2 CH_2Cl_2/MeOH). Pale yellow solid (21 mg, 81%). ^{1}H NMR (300 MHz, DMSO-d_6) δ 3.77 (s, 3H), 3.82 (s, 3H), 6.63 (dd, J = 8.4, 2.4 Hz, 1H), 6.69 (d, J = 2.4 Hz, 1H), 7.33 (d, J = 8.4 Hz, 1H), 7.42-7.51 (m, 1H), 7.56-7.60 (m, 1H), 7.73-7.81 (m, 1H), 7.95 (d, J = 2.6 Hz, 1H), 8.22 (d, J = 1.8 Hz, 1H), 8.33 (d, J = 2.0 Hz, 1H), 12.03 (s, 1H). ^{13}C NMR (75 MHz, DMSO-d_6) δ 55.3, 55.6, 99.0, 105.4, 112.4, 114.7 (d, J = 17.3 Hz, 1C), 116.6, 117.7 (d, J = 17.0 Hz, 1C), 120.6, 122.7, 124.9, 126.5, 127.5, 131.4, 132.8-132.9 (m), 144.0, 145.9 (dd, J = 244.0, 12.6 Hz, 1C), 147.6, 148.1 (dd, J = 244.3, 12.6 Hz, 1C), 157.2, 160.1. IR (cm^{-1}) ν 3108, 3006, 2869, 1257. HRMS (ES+) *m/z* calcd for $C_{21}H_{17}F_2N_2O_2$ [M + H]$^+$ 367.1258, found 367.1272.

UPLC R_t = 4.54 min; area 100%.

*3-(3-Fluoro-4-methoxyphenyl)-5-(4-benzyloxyphenyl)-1H-pyrrolo[2,3-b]pyridine (**4e**).*

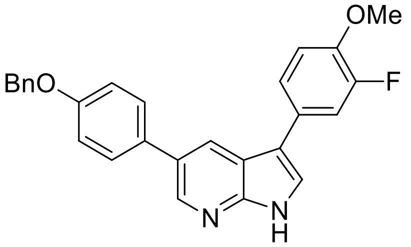

Prepared as described above from **3e** (90 mg, 0.16 mmol) and purified by preparative chromatography on silica gel (9.8:0.2 CH_2Cl_2/MeOH). Pale yellow solid (45 mg, 66%). ^{1}H NMR (300 MHz, $CDCl_3$) δ 3.96 (s, 3H), 5.15 (s, 2H), 7.04-7.14 (m, 3H), 7.35-7.59 (m, 10H),

8.32 (d, J = 2.1 Hz, 1H), 8.56 (d, J = 2.1 Hz, 1H), 11.15 (s, 1H). ^{13}C NMR (75 MHz, $CDCl_3$) δ 56.4, 70.1, 114.1 (d, J = 2.2 Hz, 1C), 114.7 (d, J = 18.7 Hz, 1C), 115.4, 116.0 (d, J = 7.1 Hz, 1C), 118.7, 122.7 (d, J = 3.8 Hz, 1C), 122.9, 126.5, 127.4, 128.0, 128.1, 128.2, 128.5 (d, J = 8.2 Hz, 1C), 129.9, 131.9, 136.9, 141.5, 146.1 (d, J = 10.4 Hz, 1C), 147.8, 151.1 (d, J = 245.9 Hz, 1C), 158.4. IR (cm^{-1}) ν 3089, 2921, 1237. HRMS (ES+) *m/z* calcd for $C_{27}H_{22}FN_2O_2$ [M + H]$^+$ 425.1665, found 425.1669.

UPLC R_t = 5.09 min; area 96%.

*3-(3,4-Dimethoxyphenyl)-5-(3,4-dibenzyloxyphenyl)-1H-pyrrolo[2,3-b]pyridine (**4f**).*

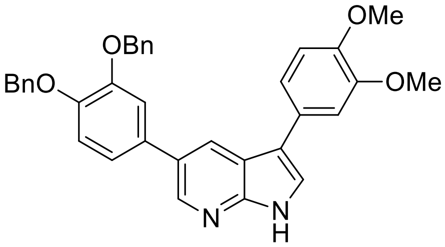

Prepared as described above from **3f** (64 mg, 0.09 mmol) and purified by preparative chromatography on silica gel (9.9:0.1 CH_2Cl_2/MeOH). Pale yellow solid (39 mg, 79%). ^{1}H NMR (300 MHz, $CDCl_3$) δ 3.95 (s, 3H), 3.96 (s, 3H), 5.24 (s, 2H), 5.25 (s, 2H), 6.99-7.06 (m, 2H), 7.12-7.15 (m, 2H), 7.18-7.21 (m, 2H), 7.31-7.42 (m, 7H), 7.48-7.54 (m, 5H), 8.30 (s, 1H), 8.49 (s, 1H). ^{13}C NMR (75 MHz, $CDCl_3$) δ 56.0, 71.3, 71.5, 110.7, 111.7, 114.6, 115.4, 116.9, 119.6, 120.5, 123.0, 127.2, 127.3, 127.4, 127.6, 127.8, 127.9, 128.5, 129.8, 132.4, 137.0, 137.1, 146.7, 148.0, 148.7, 149.2, 149.3. IR (cm^{-1}) ν 3120, 3033, 2930, 2835, 1251. HRMS (ES+) *m/z* calcd for $C_{35}H_{31}N_2O_4$ [M + H]$^+$ 543.2284, found 543.2279.

UPLC R_t = 5.17 min; area 100%.

1.4 General Procedure for Preparation of the De-*O*-methylated 3,5-Diaryl-7-azaindole Derivatives 5a-5d, 5g. To a solution of the methoxy 3,5-diaryl-1*H*-pyrrolo[2,3-*b*]pyridine derivative (**4a-4d**, **5e**, 1 equiv) in anhydrous CH_2Cl_2 (1.2 M) was added BBr_3 (1N solution in CH_2Cl_2, 3 equiv/OCH_3). The reaction mixture was stirred for 1 h at room temperature and then cooled to 0 °C before addition of excess methanol. The mixture was concentrated under vacuum and the residue was purified as described below to give the following compounds:

3-(4-Fluorophenyl)-5-(3,4-dihydroxyphenyl)-1H-pyrrolo[2,3-b]pyridine (***5a***).

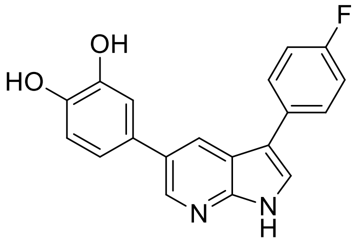

Prepared as described above from **4a** (67 mg, 0.19 mmol) and purified by preparative chromatography on silica gel (9:1 CH_2Cl_2/MeOH). White solid (21 mg, 34%). ^{1}H NMR (300 MHz, DMSO-d_6) δ 6.83 (d, J = 8.1 Hz, 1H), 6.98 (dd, J = 8.1, 2.1 Hz, 1H), 7.09 (d, J = 2.3 Hz, 1H), 7.24-7.30 (m, 2H), 7.77-7.81 (m, 2H), 7.85 (d, J = 2.4 Hz, 1H), 8.24 (d, J = 2.1 Hz, 1H), 8.43 (d, J = 2.1 Hz, 1H), 8.99 (d, J = 3.0 Hz, 2H), 11.90 (s, 1H). ^{13}C NMR (75 MHz, DMSO-d_6) δ 113.4, 114.4, 115.5 (d, J = 21.4 Hz, 2C), 116.1, 117.1, 118.0, 124.3, 128.0 (d, J = 7.7 Hz, 2C), 129.2, 130.3, 131.5 (d, J = 3.3 Hz, 1C), 141.6, 144.8, 145.7, 148.1, 158.9 (d, J = 242.1 Hz, 1C). IR (cm^{-1}) v 3246, 3044, 2926, 1217. HRMS (ES+) *m/z* calcd for $C_{19}H_{14}FN_2O_2$ $[M + H]^+$ 321.1039, found 321.1045.

UPLC R_t = 3.25 min; area 100%.

Large scale preparation of ***5a***. Prepared as described above from **4a** (760 mg, 2.18 mmol) and purified by precipitation in CH_2Cl_2 and washing with CH_2Cl_2 then MeOH. White solid (590 mg, 84%). ^{1}H NMR (300 MHz, CD_3OD) δ 6.94 (d, J = 8.1 Hz, 1H), 7.10 (dd, J = 8.1, 2.1 Hz, 1H), 7.17 (d, J = 2.1 Hz, 1H), 7.24-7.30 (m, 2H), 7.76-7.81 (m, 2H), 7.93 (s, 1H), 8.60 (d, J = 1.4 Hz, 1H), 8.86 (d, J = 3.6 Hz, 1H).

*3-(4-Fluorophenyl)-5-(2,4-dihydroxyphenyl)-1H-pyrrolo[2,3-b]pyridine (**5b**).*

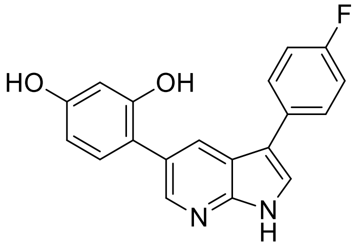

Prepared as described above from **4b** (52 mg, 0.15 mmol) and purified by preparative chromatography on silica gel (93:7 CH_2Cl_2/MeOH). White solid (24 mg, 50%). ^{1}H NMR (300 MHz, CD_3OD) δ 6.42-6.45 (m, 2H), 7.14-7.19 (m, 3H), 7.56-7.59 (m, 1H), 7.66-7.70 (m, 2H), 8.33 (s, 1H), 8.38 (s, 1H). ^{13}C NMR (75 MHz, CD_3OD) δ 104.1, 108.5, 116.3, 116.4 (d, J = 21.4 Hz, 2C), 119.3, 119.5, 124.2, 129.0, 129.5, (d, J = 7.7 Hz, 2C), 129.8, 132.5, 132.8 (d, J = 3.3 Hz, 1C), 144.8, 148.3, 156.5, 159.2, 161.2 (d, J = 242.6 Hz, 1C). IR (cm^{-1}) ν 3303, 2586, 1220. HRMS (ES+) *m/z* calcd for $C_{19}H_{14}FN_2O_2$ $[M + H]^+$ 321.1039, found 321.1024. UPLC R_t = 3.07 min; area 100%.

*3-(3,4-Difluorophenyl)-5-(3,4-dihydroxyphenyl)-1H-pyrrolo[2,3-b]pyridine (**5c**).*

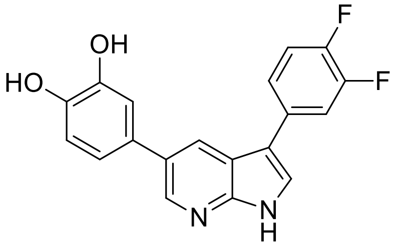

Prepared as described above from **4c** (27 mg, 0.07 mmol) and purified by trituration with MeOH/CH_2Cl_2. Yellow solid (24 mg, 100%). ^{1}H NMR (300 MHz, DMSO-d_6) δ 6.85 (d, J = 8.1 Hz, 1H), 7.05 (d, J = 8.3 Hz, 1H), 7.16 (s, 1H), 7.45-7.55 (m, 1H), 7.64-7.68 (m, 1H), 7.83-7.90 (m, 1H), 8.05 (s, 1H), 8.48-8.54 (m, 2H), 12.38 (s, 1H). ^{13}C NMR (75 MHz, DMSO-d_6) δ 113.7, 115.1, 115.6 (d, J = 17.6 Hz, 1C), 116.6, 118.3 (d, J = 16.7 Hz, 1C), 118.8, 119.1 (d, J = 5.2 Hz, 1C), 123.6, 126.5-126.7 (m), 127.6-127.7 (m), 129.6, 130.1, 132.5, 139.3-139.7 (m), 145.7, 146.2, 146.7 (dd, J = 244.5, 12.6 Hz, 1C), 147.0, 148.6 (dd, J

= 244.5, 12.6 Hz, 1C). IR (cm^{-1}) ν 3117, 2924, 1269. HRMS (ES+) *m/z* calcd for $C_{19}H_{13}F_2N_2O_2$ $[M + H]^+$ 339.0945, found 339.0932.

UPLC R_t = 3.43 min; area 100%.

*3-(3,4-Difluorophenyl)-5-(2,4-dihydroxyphenyl)-1H-pyrrolo[2,3-b]pyridine (**5d**).*

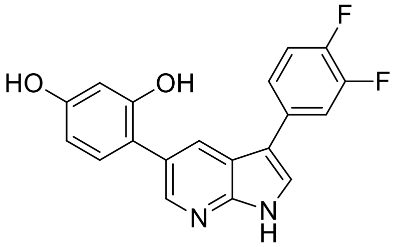

Prepared as described above from **4d** (10 mg, 0.03 mmol) and purified by trituration with MeOH/CH_2Cl_2. Yellow solid (9 mg, 100%). ^{1}H NMR (300 MHz, CD_3OD) δ 6.45-6.49 (m, 2H), 7.29-7.34 (m, 1H), 7.37-7.43 (m, 1H), 7.50-7.54 (m, 1H), 7.59-7.66 (m, 1H), 7.95 (s, 1H), 8.63 (d, J = 1.1 Hz, 1H), 8.94 (d, J = 1.5 Hz, 1H). ^{13}C NMR (75 MHz, CD_3OD) δ 104.0, 109.1, 115.0, 117.3 (d, J = 18.1 Hz, 1C), 117.8, 119.0 (d, J = 18.1 Hz, 1C), 124.8, 125.0-125.2 (m, 1C), 128.3, 130.0, 131.2, 132.3, 134.7, 138.2, 139.0, 149.3 (dd, J = 247.6, 12.6 Hz, 1C), 150.2 (dd, J = 247.0, 12.6 Hz, 1C), 156.8, 160.8. IR (cm^{-1}) ν 3095, 2922, 2851, 1267. HRMS (ES+) *m/z* calcd for $C_{19}H_{13}F_2N_2O_2$ $[M + H]^+$ 339.0945, found 339.0933.

UPLC R_t = 3.27 min; area 100%.

*3-(3-Fluoro-4-hydroxyphenyl)-5-(4-hydroxyphenyl)-1H-pyrrolo[2,3-b]pyridine (**5g**).*

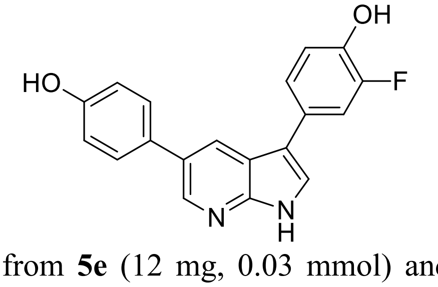

Prepared as described above from **5e** (12 mg, 0.03 mmol) and purified by trituration with MeOH/CH_2Cl_2. Yellow solid (10 mg, 100%). ^{1}H NMR (300 MHz, CD_3OD) δ 6.93-6.96 (m, 2H), 7.03 (t, J = 8.8 Hz, 1H). 7.34-7.38 (m, 1H), 7.39 (dd, J = 12.1, 2.1 Hz, 1H), 7.56-7.59 (m, 2H), 7.79 (s, 1H), 8.56 (s, 1H), 8.74 (d, J = 1.3 Hz, 1H). ^{13}C NMR (75 MHz, CD_3OD) δ 115.9 (d, J = 19.2 Hz, 1C), 117.2, 119.4 (d, J = 3.3 Hz, 1C), 124.7 (d, J = 2.7 Hz, 1C), 126.2

(d, J = 6.0 Hz, 1C), 126.8, 129.0, 129.7, 133.3, 135.2, 142.1, 145.5 (d, J = 12.6 Hz, 1C), 151.6 (d, J = 241.0 Hz, 1C), 153.5, 153.8, 154.1, 159.2. IR (cm^{-1}) ν 3173, 2922, 1259. HRMS (ES+) *m/z* calcd for $C_{19}H_{14}FN_2O_2$ $[M + H]^+$ 321.1039, found 321.1045.

UPLC R_t = 2.68 min; area 95%.

*3-(3-Fluoro-4-methoxyphenyl)-5-(4-hydroxyphenyl)-1H-pyrrolo[2,3-b]pyridine (**5e**).*

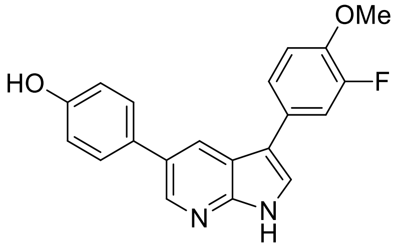

To a solution of **4e** (26 mg, 0.06 mmol) in degassed MeOH (1 mL) were added 10% palladium on charcoal (10 mg) and ammonium formate (19 mg, 0.30 mmol). The reaction mixture was heated for 15 h at 35 °C under argon, then cooled and filtered through Celite. The filter pad was washed with MeOH and CH_2Cl_2, the filtrates were combined and concentrated under vacuum to afford compound **5e** as a white solid (20 mg, 100%). ^{1}H NMR (300 MHz, DMSO-d_6) δ 3.87 (s, 3H), 6.87 (d, J = 8.7 Hz, 2H), 7.20 (t, J = 8.9 Hz, 1H), 7.55 (d, J = 8.5 Hz, 4H), 7.86 (s, 1H), 8.29 (d, J = 2.1 Hz, 1H), 8.47 (d, J = 1.9 Hz, 1H), 9.69 (s, 1H), 11.93 (s, 1H). ^{13}C NMR (75 MHz, DMSO-d_6) δ 56.1, 113.2 (d, J = 2.2 Hz, 1C), 113.7 (d, J = 18.7 Hz, 1C), 114.4 (d, J = 1.6 Hz, 1C), 115.9, 117.1, 122.4 (d, J = 2.7 Hz, 1C), 124.3, 124.5, 128.2, 128.3 (d, J = 7.1 Hz, 1C), 129.0, 129.6, 141.7, 145.1 (d, J = 11.0 Hz, 1C), 148.1, 150.3 (d, J = 243.2 Hz, 1C), 157.0. IR (cm^{-1}) ν 3371, 3015, 2931, 1266. HRMS (ES+) *m/z* calcd for $C_{20}H_{16}FN_2O_2$ $[M + H]^+$ 335.1196, found 335.1191.

UPLC R_t = 3.42 min; area 95%.

*3-(3,4-Dimethoxyphenyl)-5-(3,4-dihydroxyphenyl)-1H-pyrrolo[2,3-b]pyridine (**5f**).*

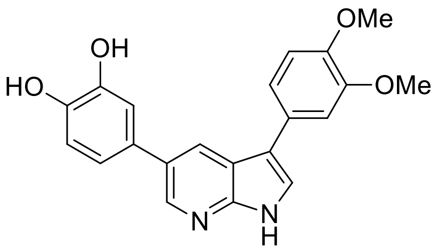

To a solution of **4f** (15 mg, 0.03 mmol) in degassed MeOH (1 mL) were added 10% palladium on charcoal (10 mg) and ammonium formate (15 mg, 0.24 mmol). The reaction mixture was heated for 3 h at 35 °C under argon, then cooled and filtered through Celite. The filter pad was washed with EtOH and CH_2Cl_2, the filtrates were combined and concentrated under vacuum to afford compound **5f** as a white solid (11 mg, 100%). ^{1}H NMR (300 MHz, CD_3OD) δ 3.86 (s, 3H), 3.90 (s, 3H), 6.87 (d, J = 8.1 Hz, 1H), 6.95 (dd, J = 8.1, 2.1 Hz, 1H), 7.02-7.04 (m, 1H), 7.08 (d, J = 2.1 Hz, 1H), 7.21-7.24 (m, 2H), 7.57 (s, 1H), 8.28 (d, J = 1.9 Hz, 1H), 8.38 (s, 1H), 8.50 (s, 1H). ^{13}C NMR (75 MHz, CD_3OD) δ 56.6, 112.2, 113.6, 115.3, 117.0, 117.2, 119.7, 120.1, 120.6, 124.3, 127.1, 129.6, 131.3, 132.5, 142.3, 146.1, 146.8, 148.9, 149.1, 150.8. IR (cm^{-1}) ν 3420, 3005, 2922, 2851, 1251. HRMS (ES+) *m/z* calcd for $C_{21}H_{19}N_2O_4$ $[M + H]^+$ 363.1345, found 363.1337.

UPLC R_t = 2.74 min; area 100%.

2. NMR Spectra of 2a-2d, 3a-3e, 4a-4f and 5a-5g

3-(4-Fluorophenyl)-5-bromo-1-(phenylsulfonyl)-1H-pyrrolo[2,3-b]pyridine (***2a***).

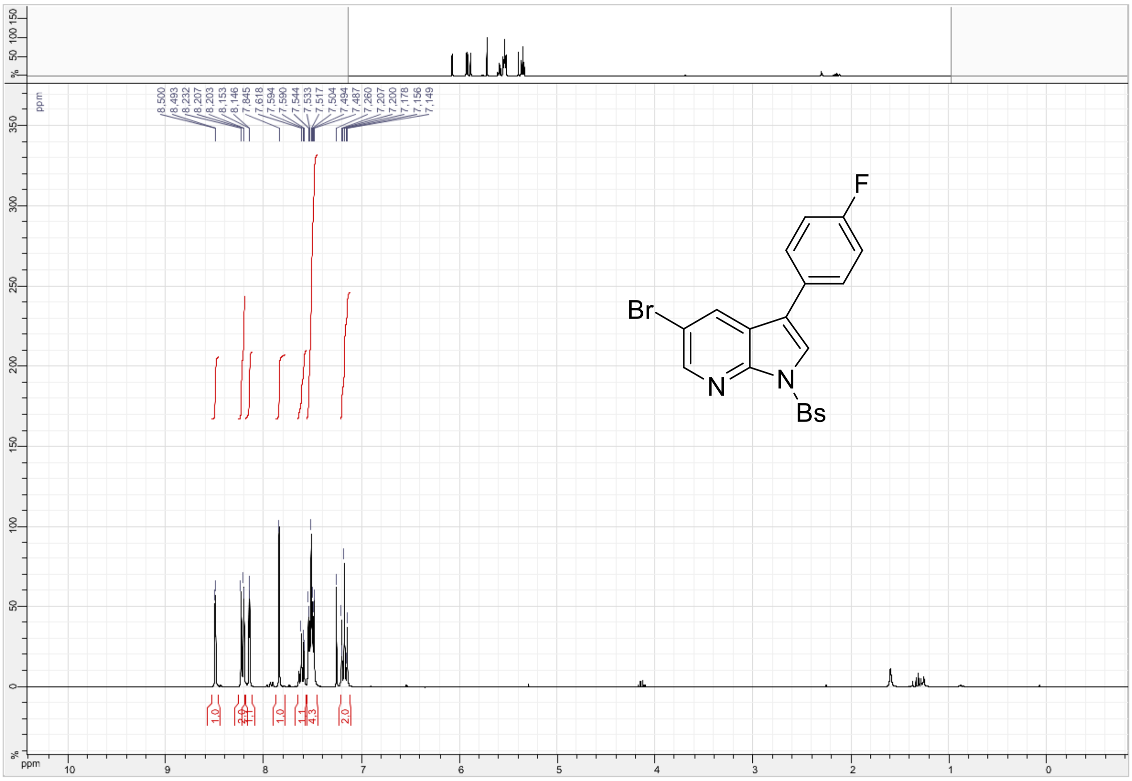

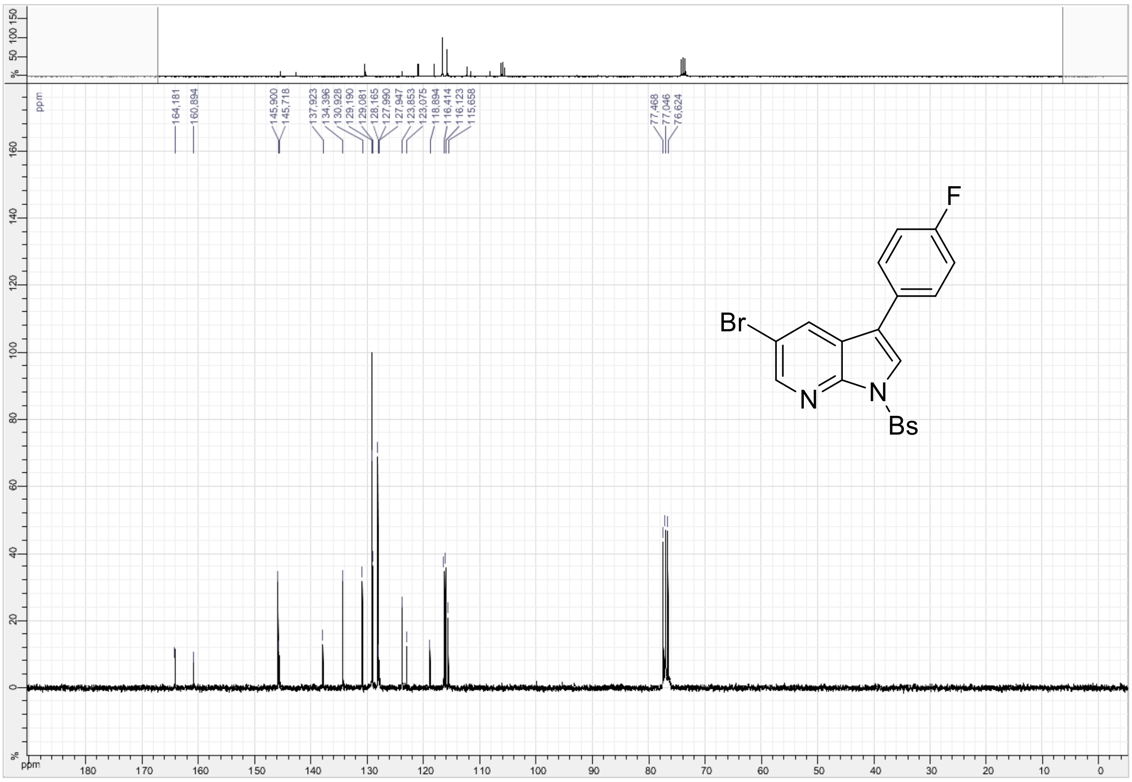

3-(3,4-Difluorophenyl)-5-bromo-1-(phenylsulfonyl)-1H-pyrrolo[2,3-b]pyridine (***2b***).

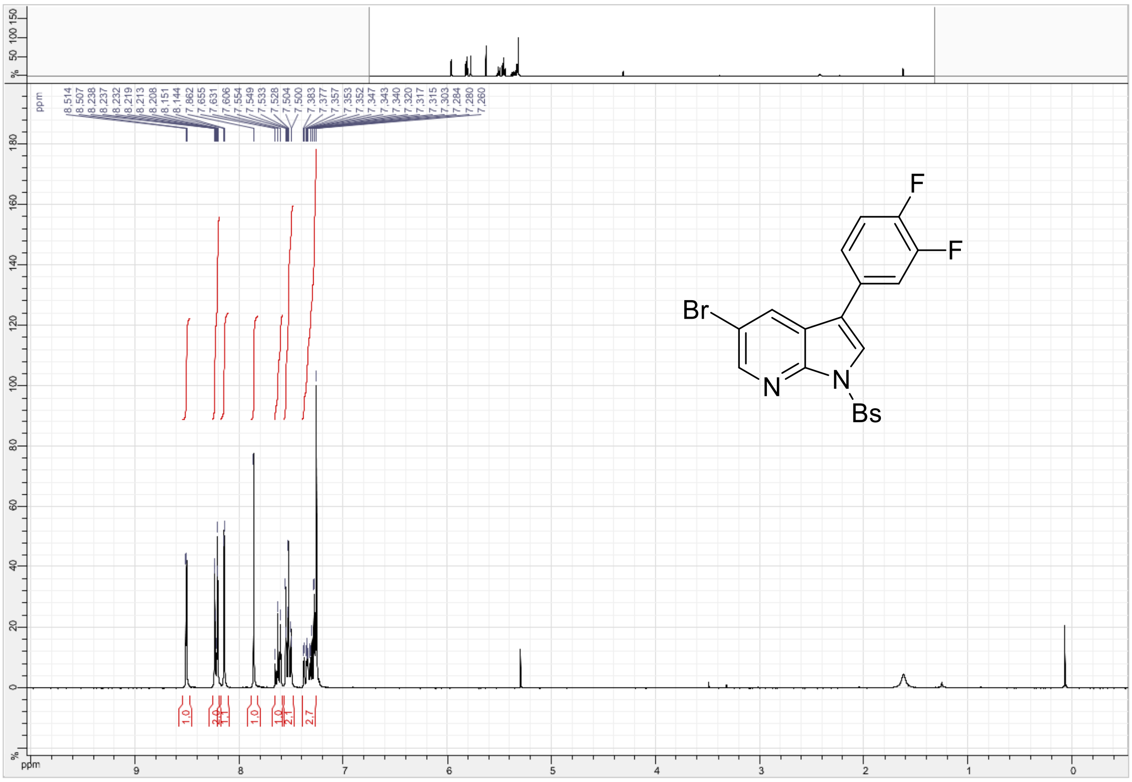

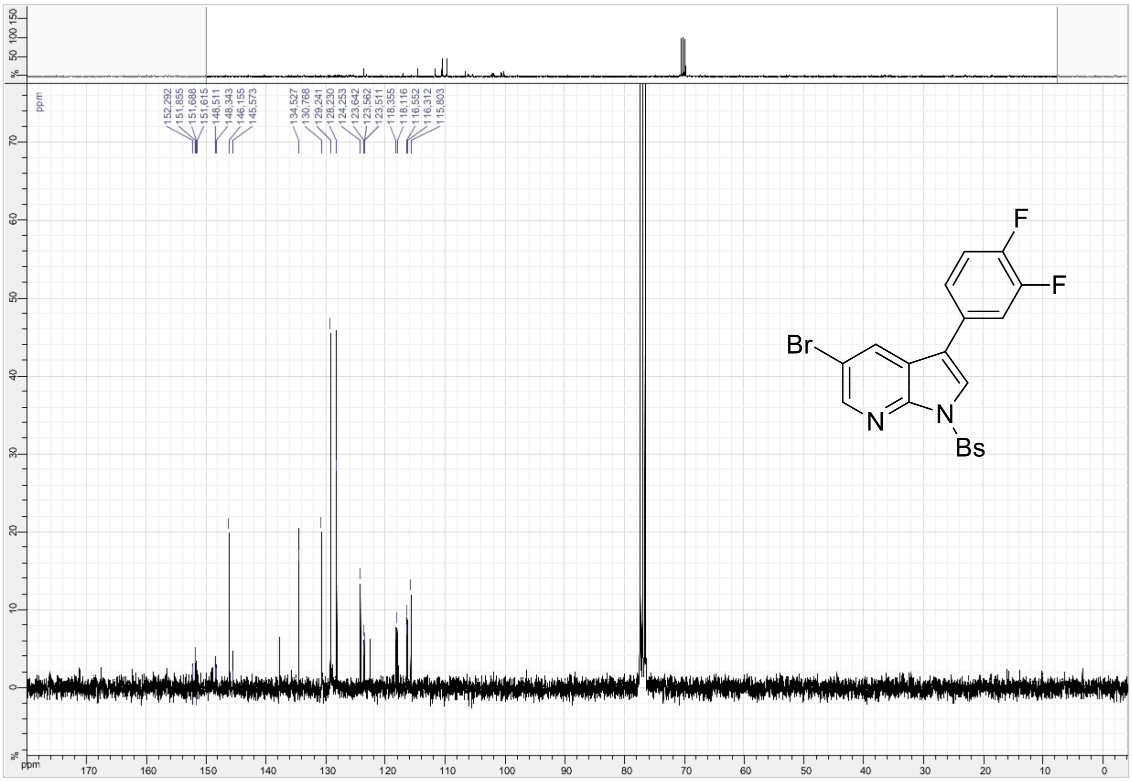

3-(3-Fluoro-4-methoxyphenyl)-5-bromo-1-(phenylsulfonyl)-1H-pyrrolo[2,3-b]pyridine (**2c**).

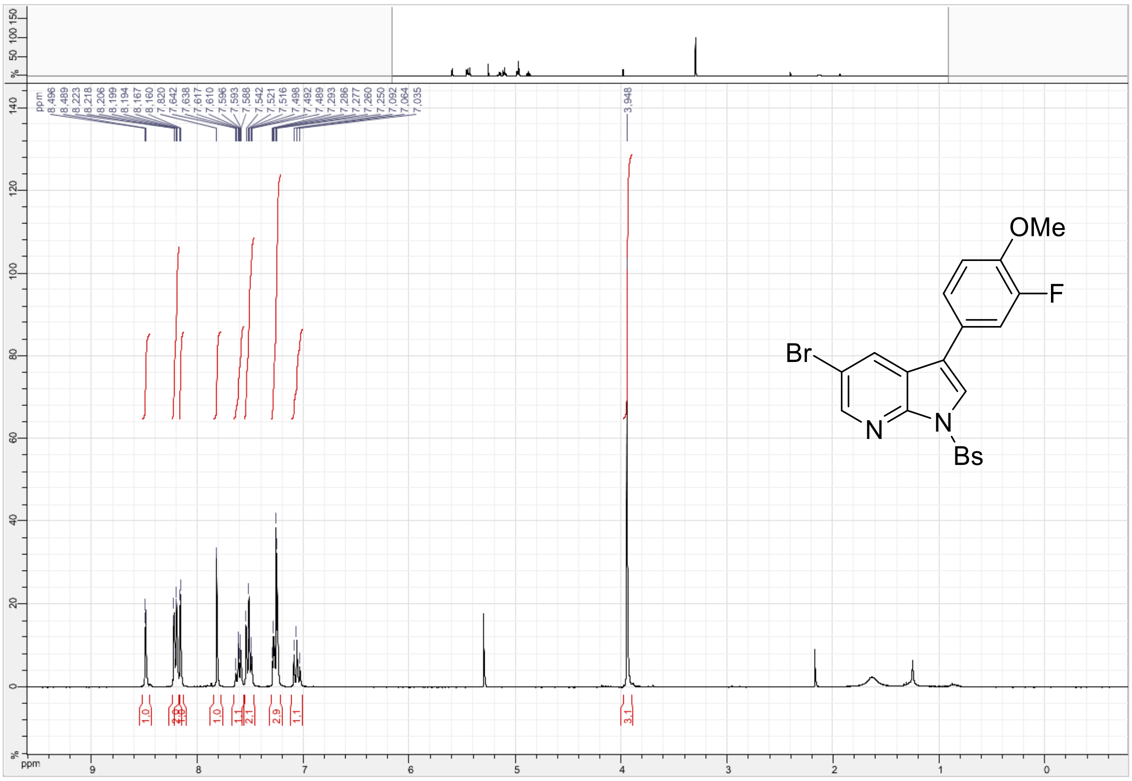

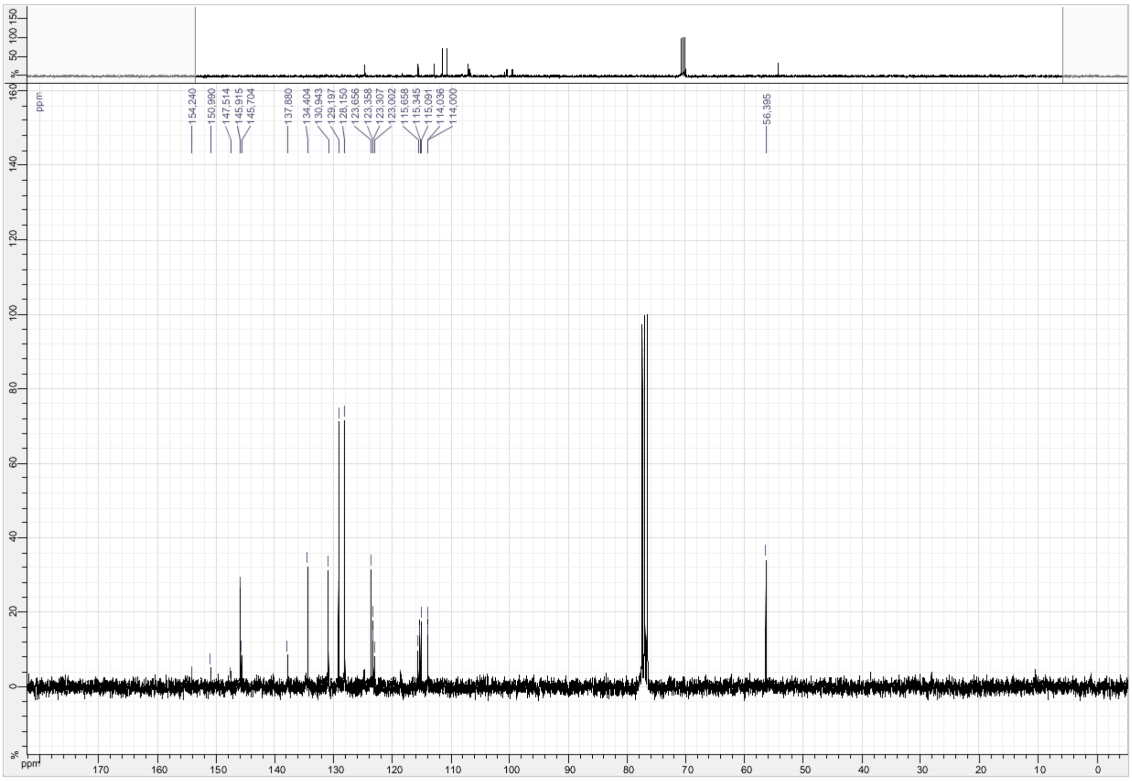

3-(3,4-Dimethoxyphenyl)-5-bromo-1-(phenylsulfonyl)-1H-pyrrolo[2,3-b]pyridine (**2d**).

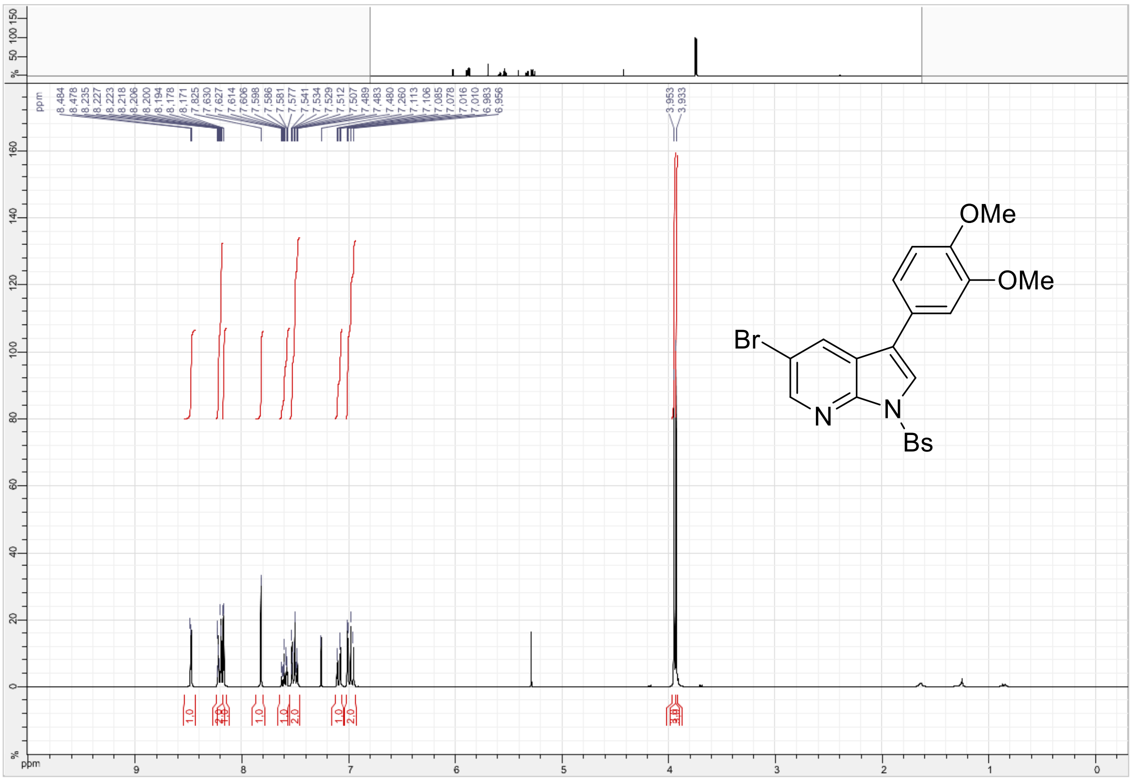

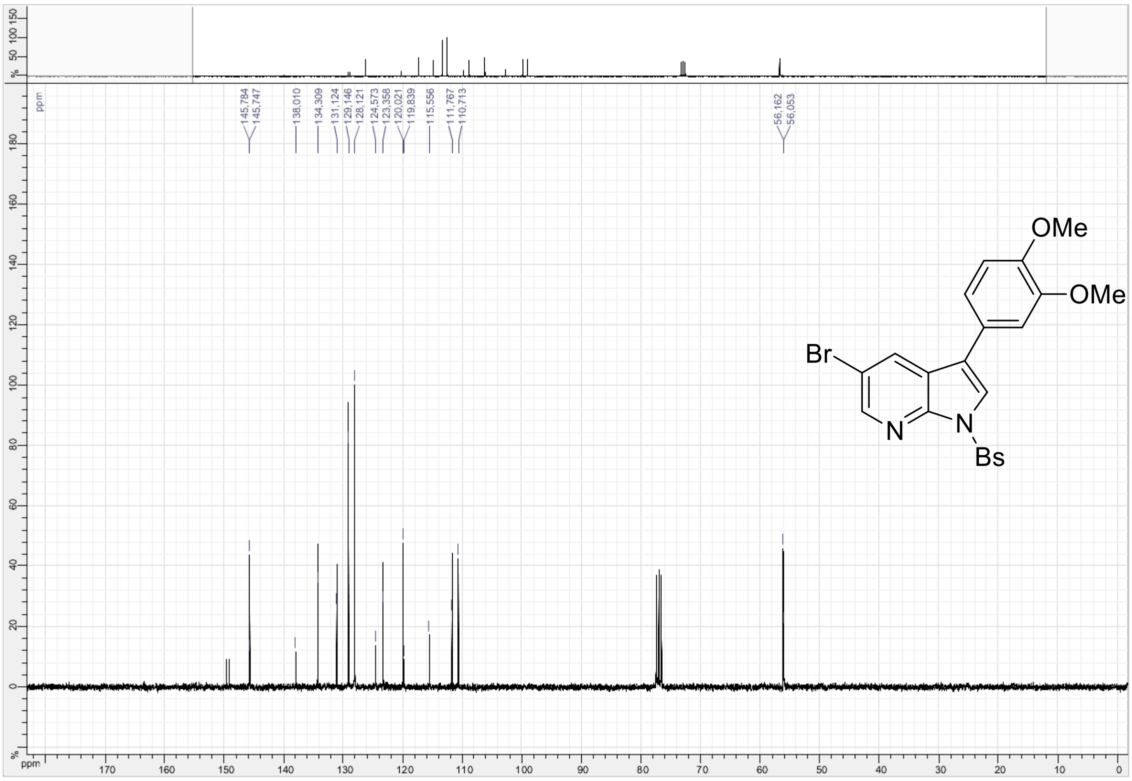

3-(4-Fluorophenyl)-5-(3,4-dimethoxyphenyl)-1-(phenylsulfonyl)-1H-pyrrolo[2,3-b]pyridine (***3a***).

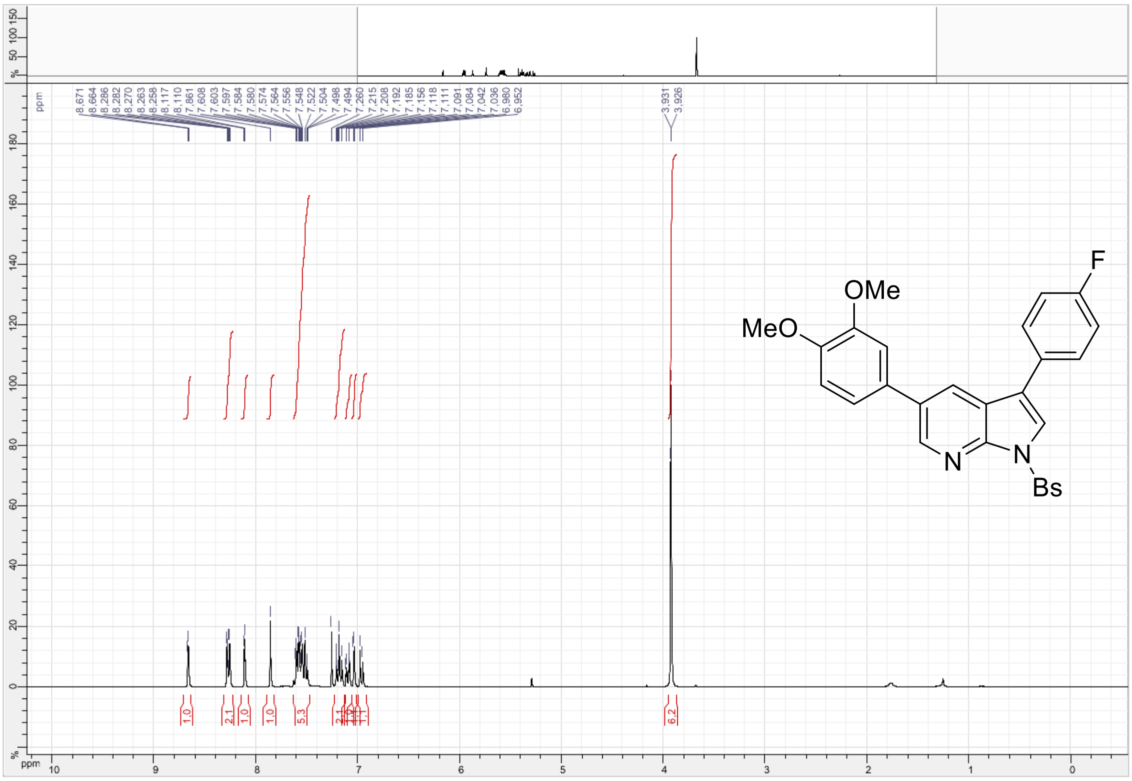

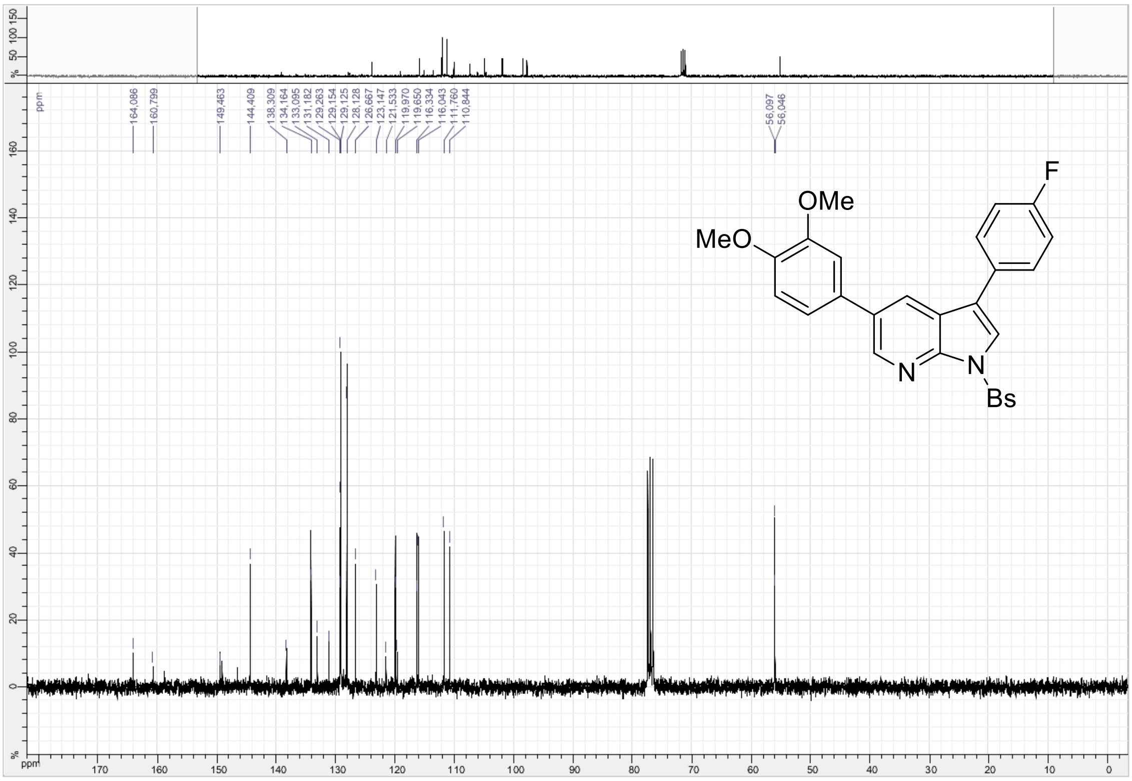

*3-(4-Fluorophenyl)-5-(2,4-dimethoxyphenyl)-1-(phenylsulfonyl)-1H-pyrrolo[2,3-b]pyridine (**3b**).*

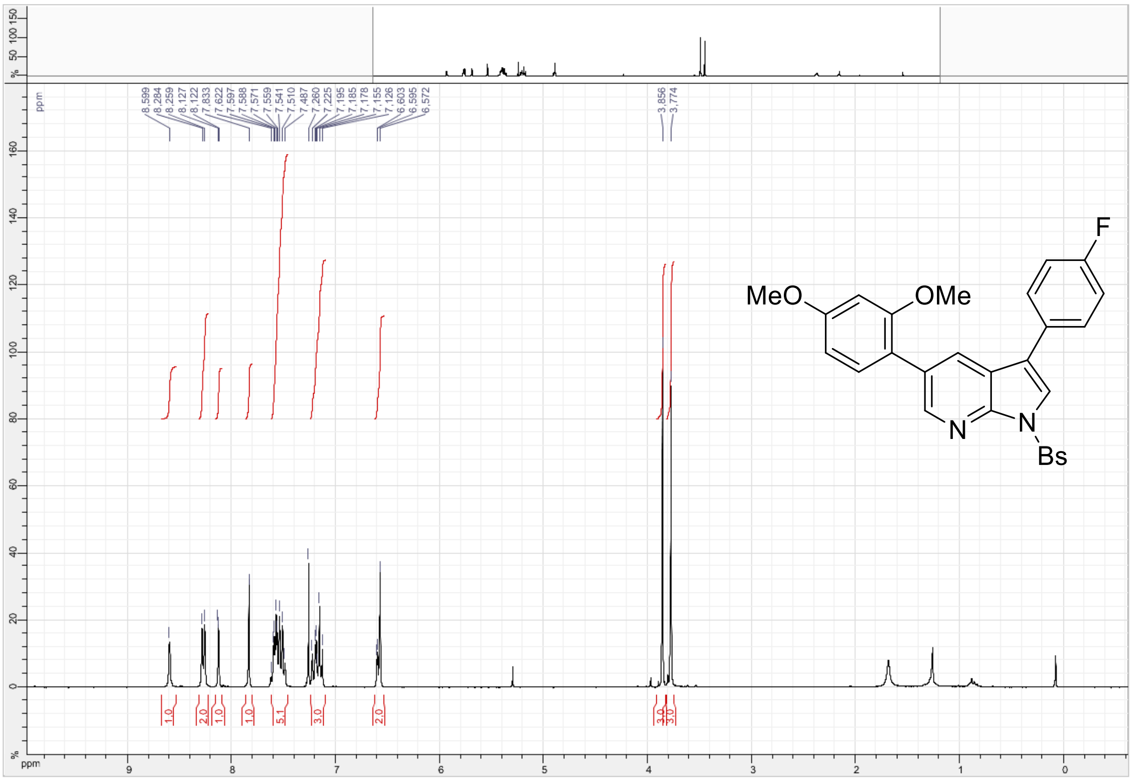

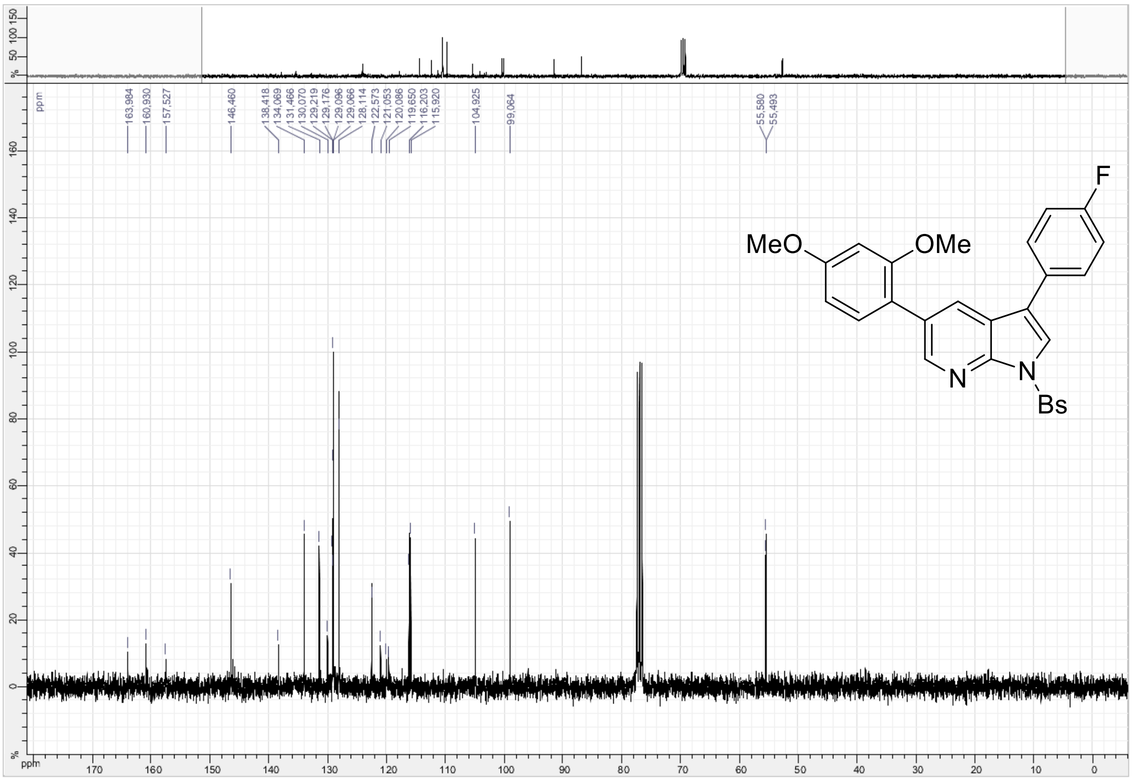

*3-(3,4-Difluorophenyl)-5-(3,4-dimethoxyphenyl)-1-(phenylsulfonyl)-1H-pyrrolo[2,3-b]pyridine (**3c**).*

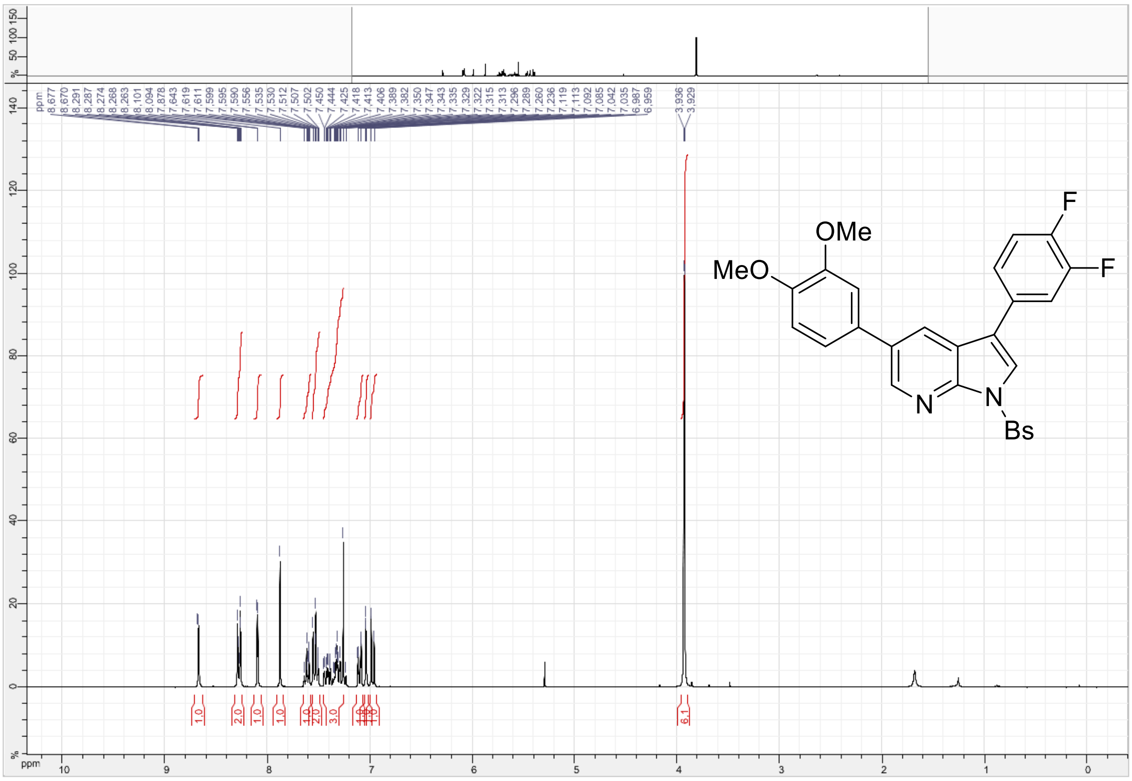

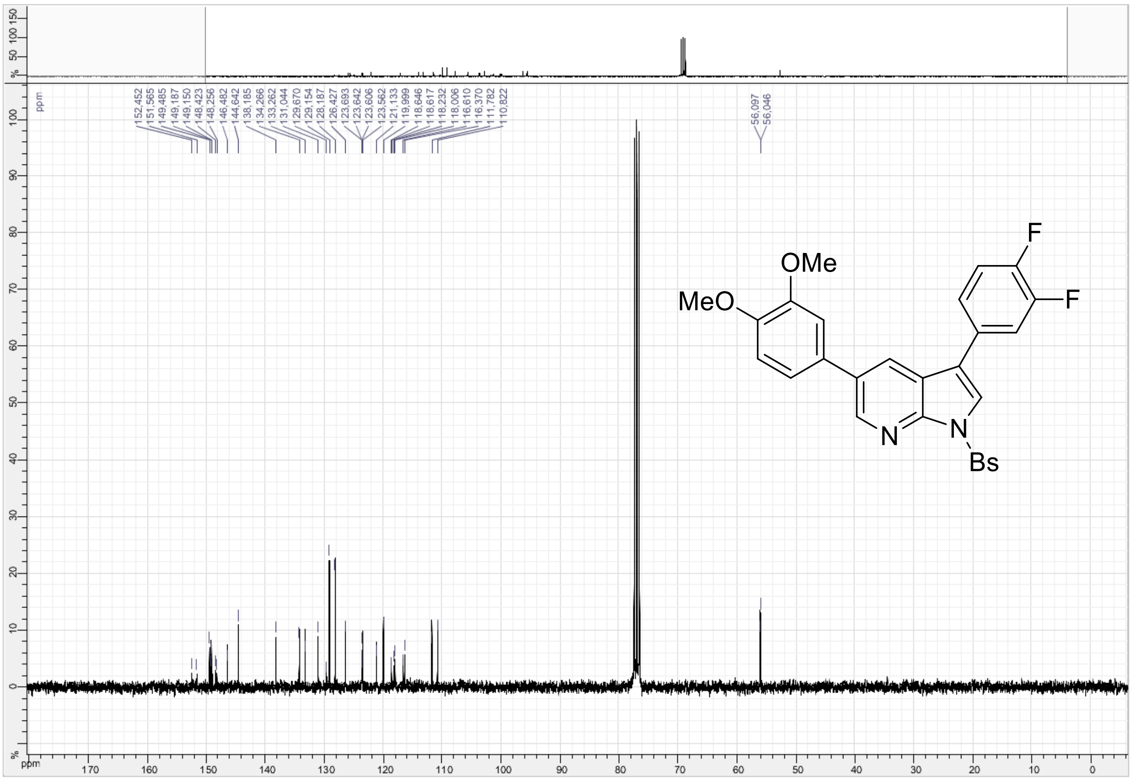

*3-(3,4-Difluorophenyl)-5-(2,4-dimethoxyphenyl)-1-(phenylsulfonyl)-1H-pyrrolo[2,3-b]pyridine (**3d**).*

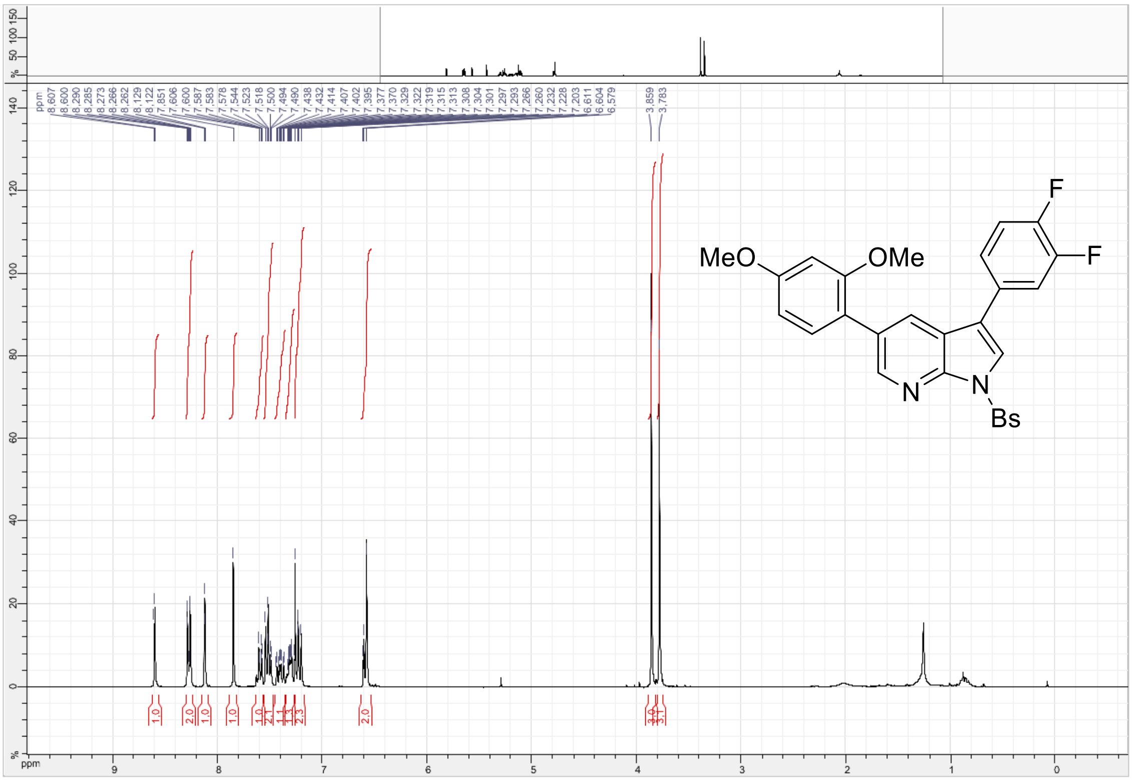

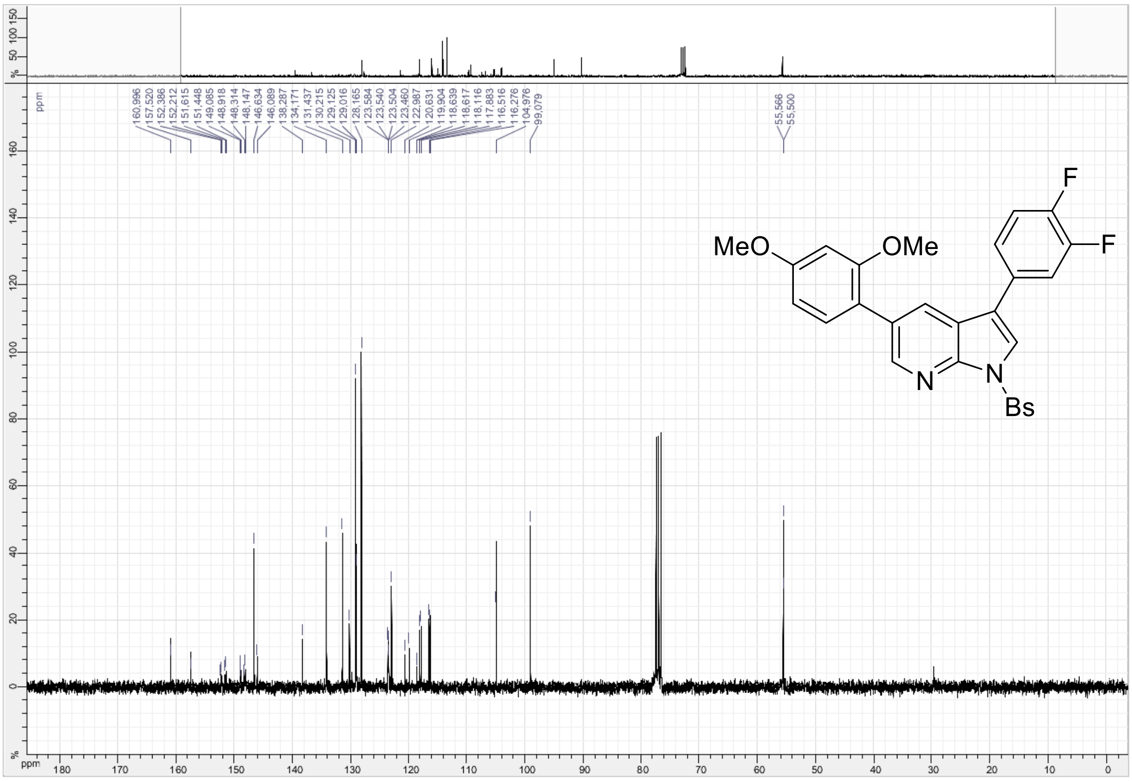

*3-(3-Fluoro-4-methoxyphenyl)-5-(4-benzyloxyphenyl)-1-(phenylsulfonyl)-1H-pyrrolo[2,3-b]pyridine (**3e**).*

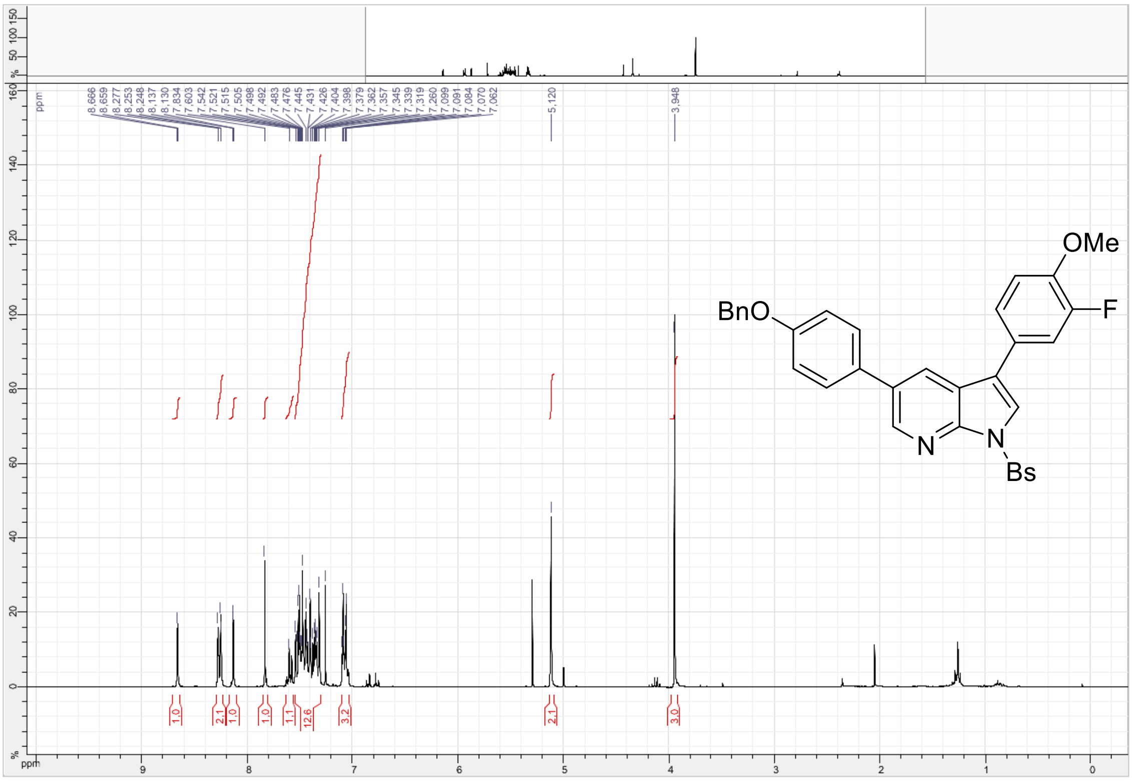

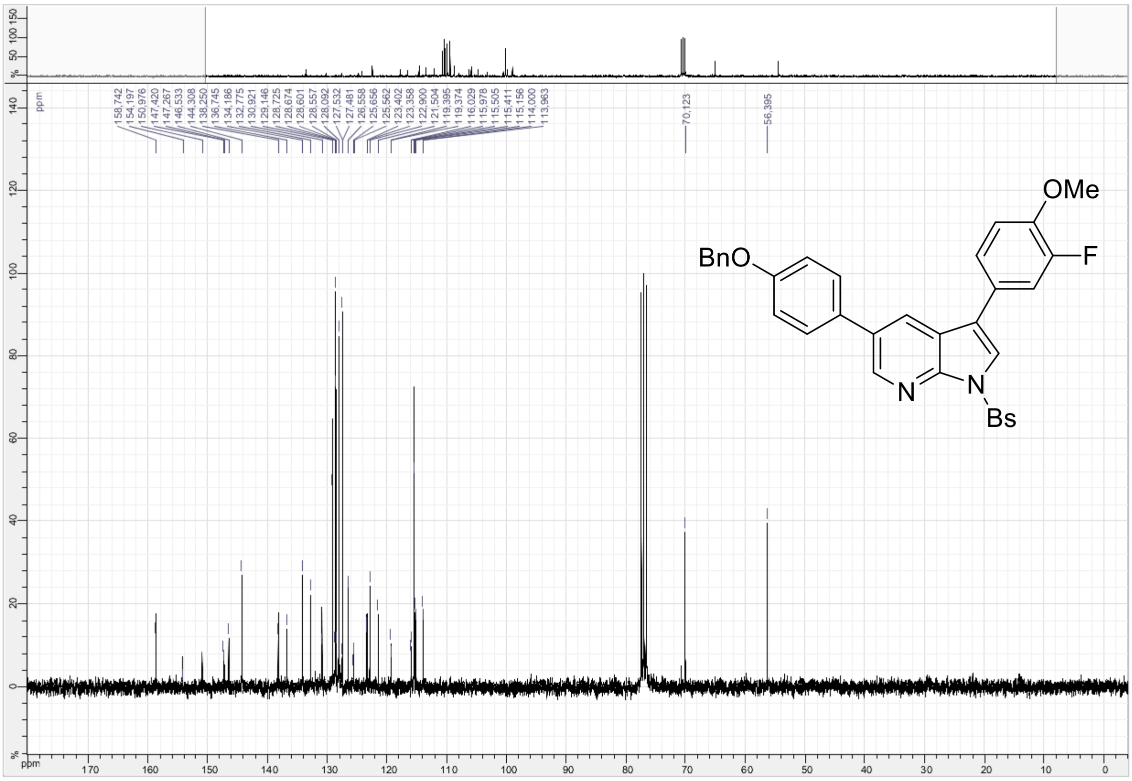

*3-(3,4-Dimethoxyphenyl)-5-(3,4-dibenzyloxyphenyl)-1-(phenylsulfonyl)-1H-pyrrolo[2,3-b]pyridine (**3f**).*

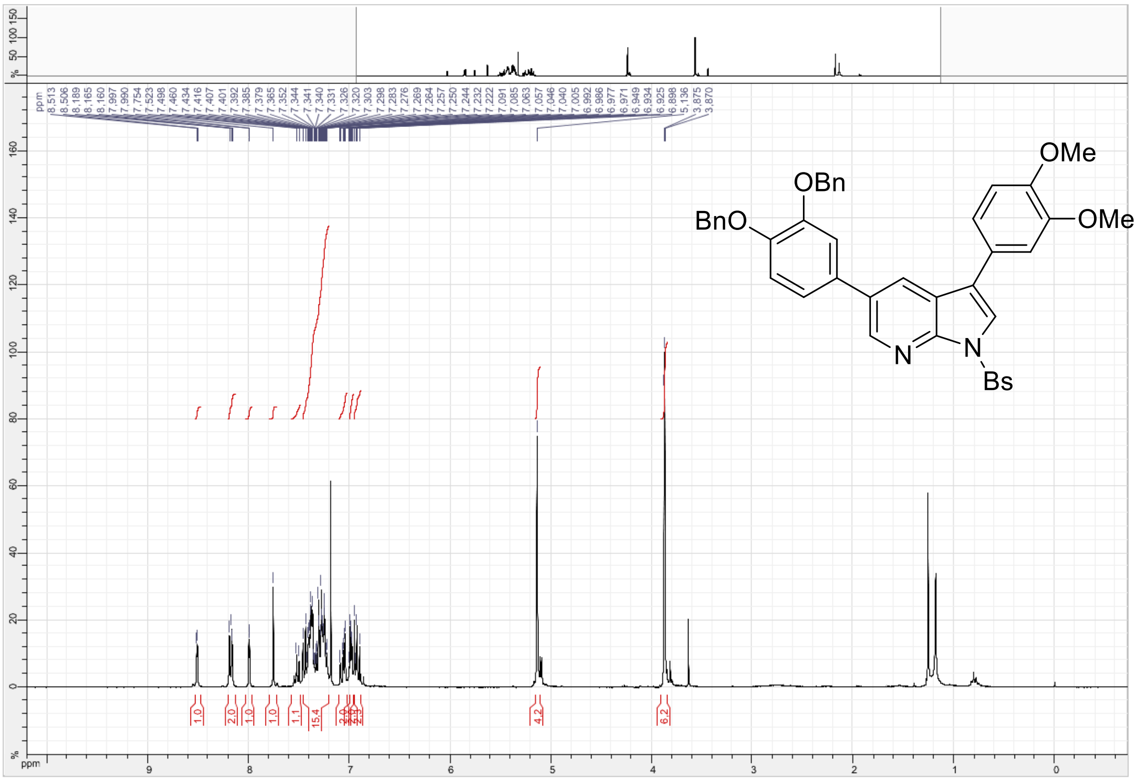

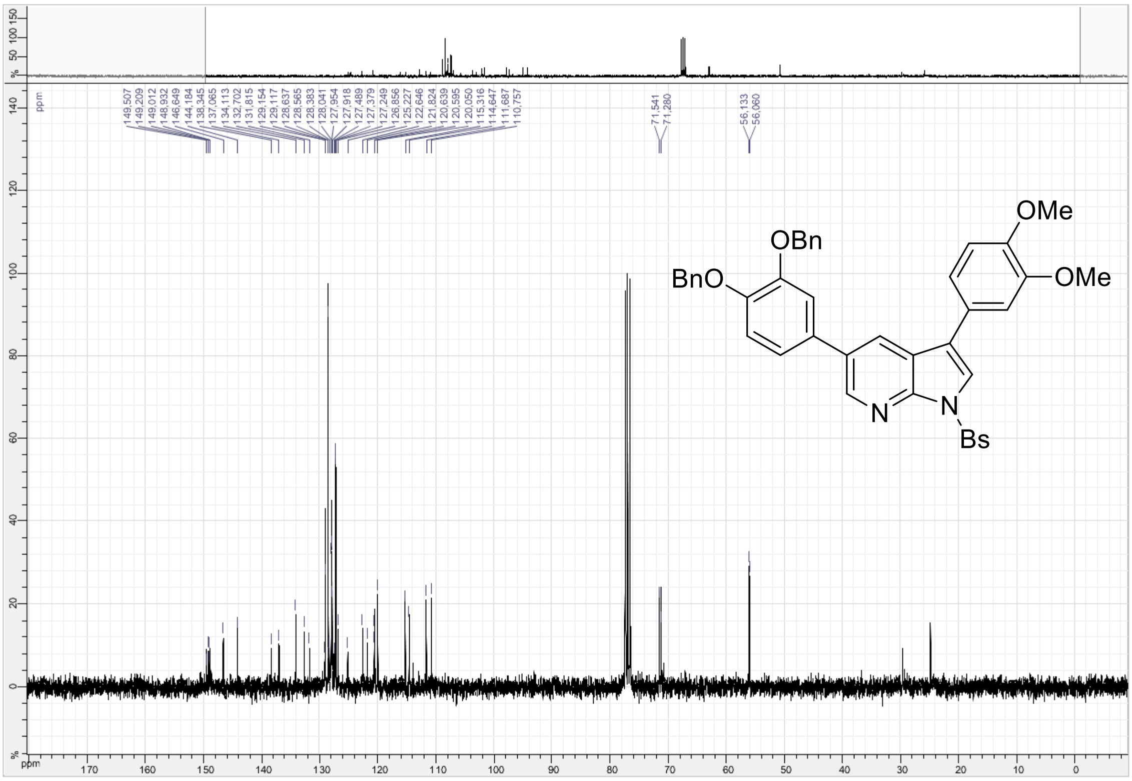

3-(4-Fluorophenyl)-5-(3,4-dimethoxyphenyl)-1H-pyrrolo[2,3-b]pyridine (***4a***).

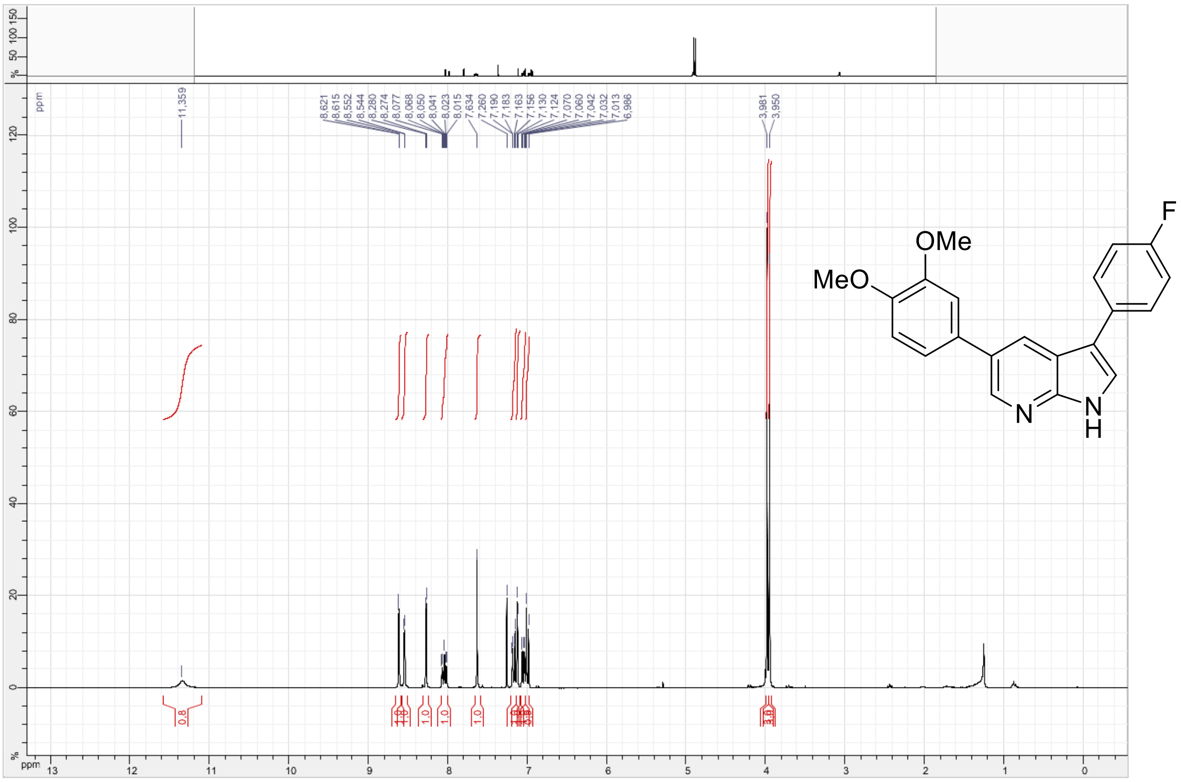

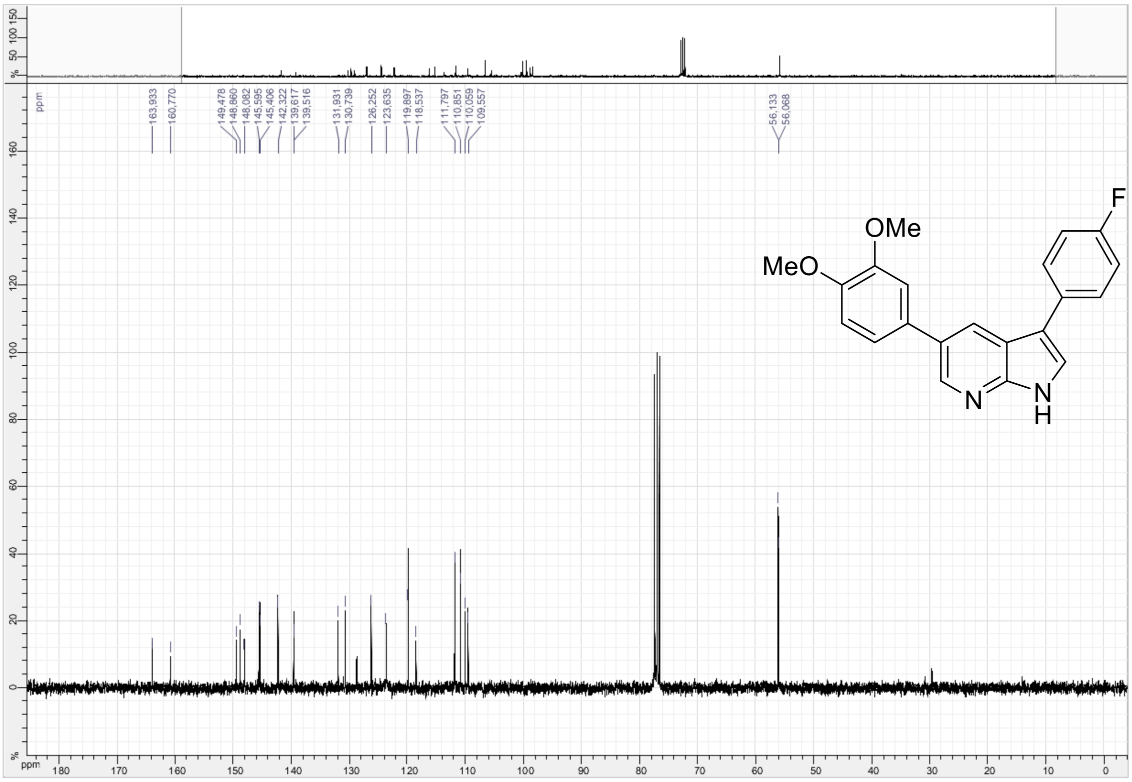

*3-(4-Fluorophenyl)-5-(2,4-dimethoxyphenyl)-1H-pyrrolo[2,3-b]pyridine (**4b**).*

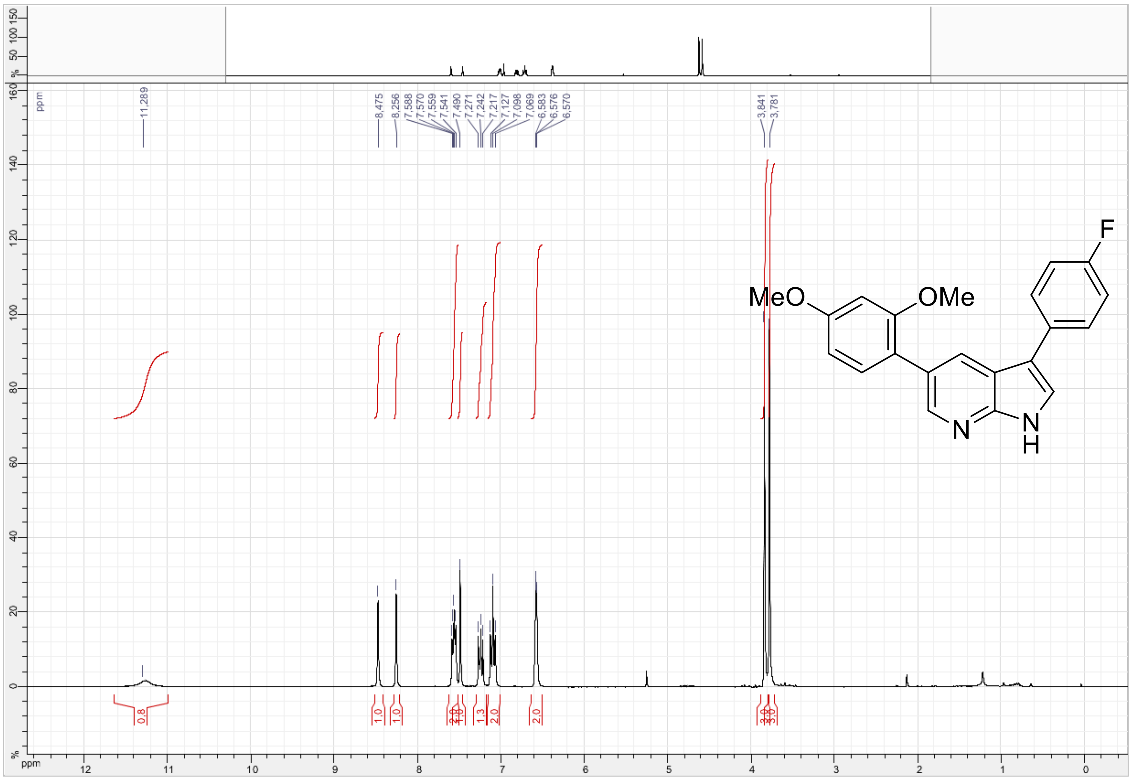

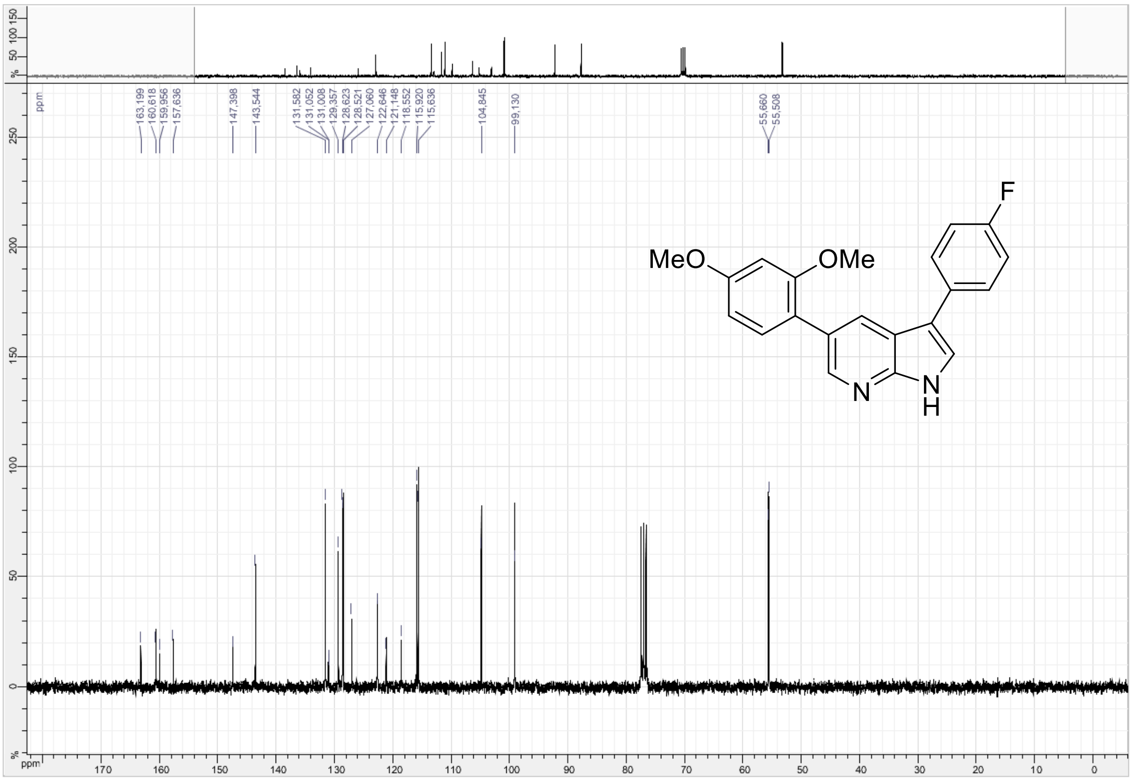

3-(3,4-Difluorophenyl)-5-(3,4-dimethoxyphenyl)-1H-pyrrolo[2,3-b]pyridine (**4c**).

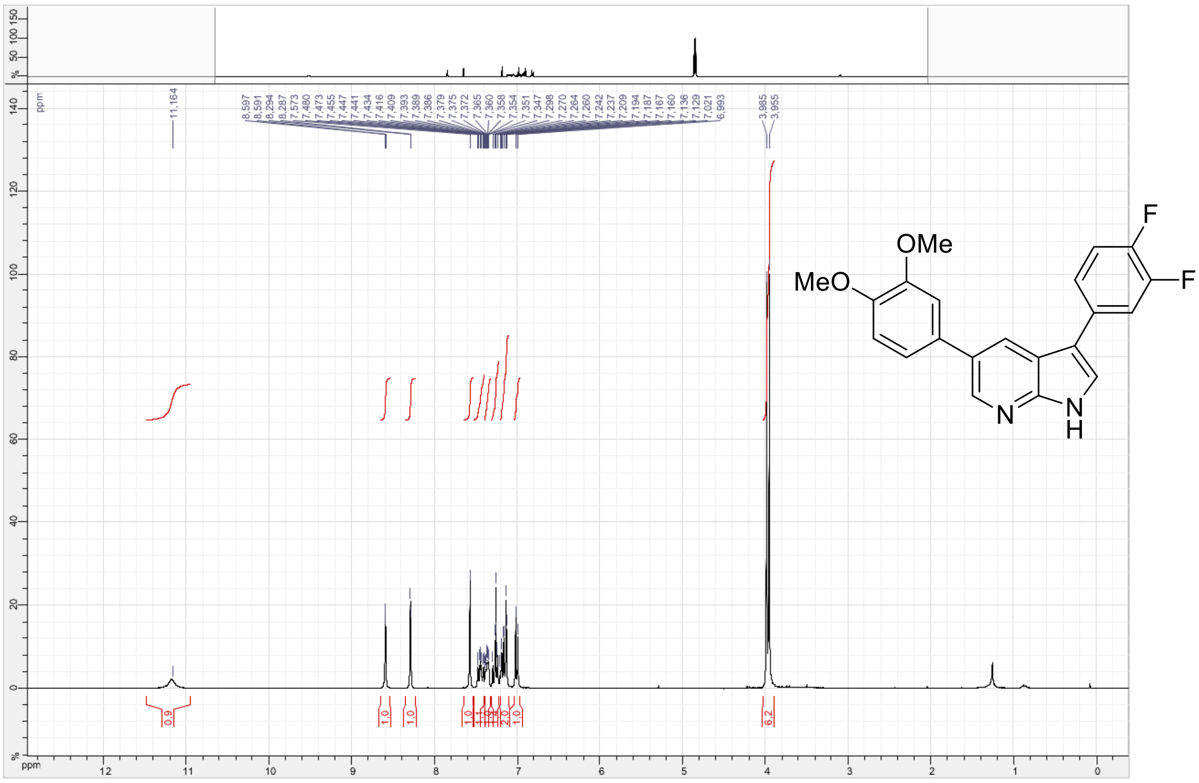

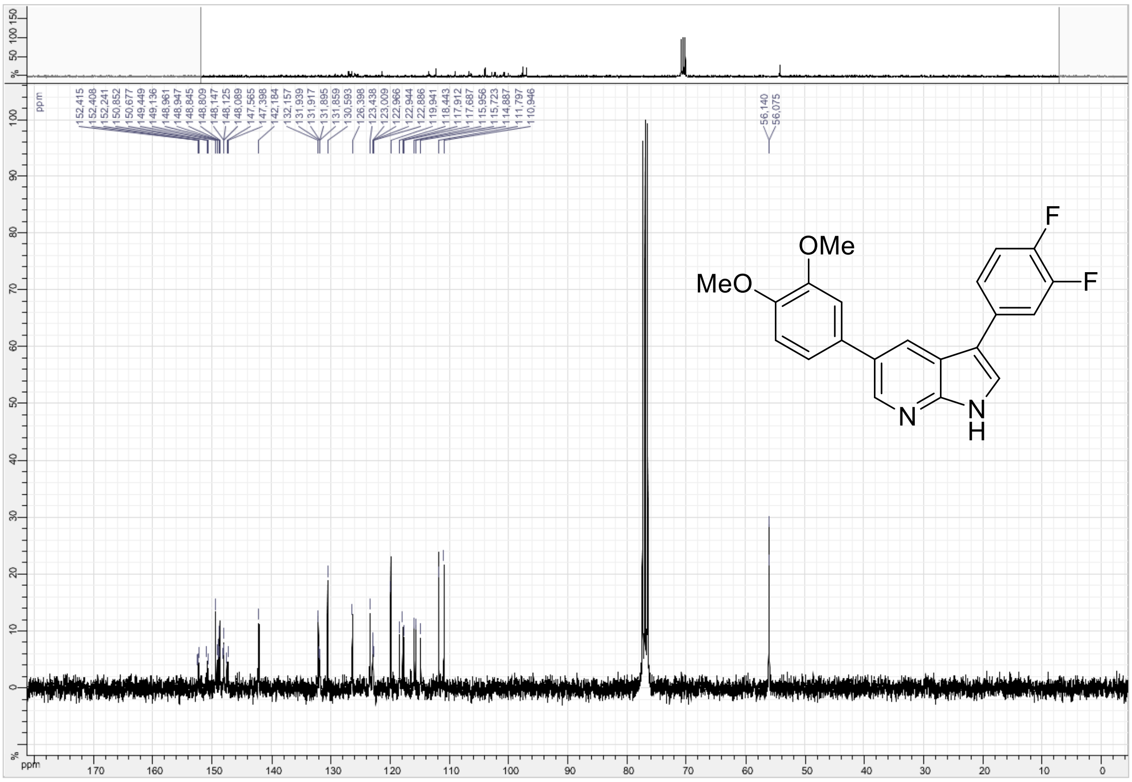

*3-(3,4-Difluorophenyl)-5-(2,4-dimethoxyphenyl)-1H-pyrrolo[2,3-b]pyridine (**4d**).*

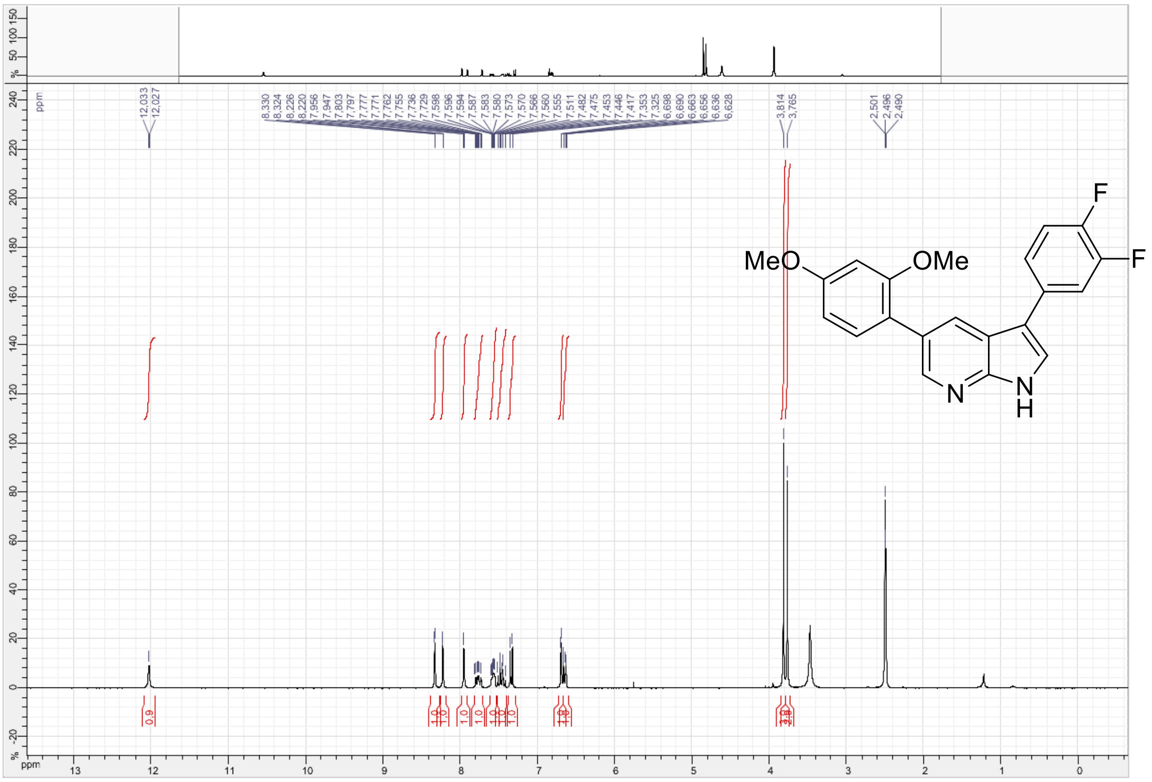

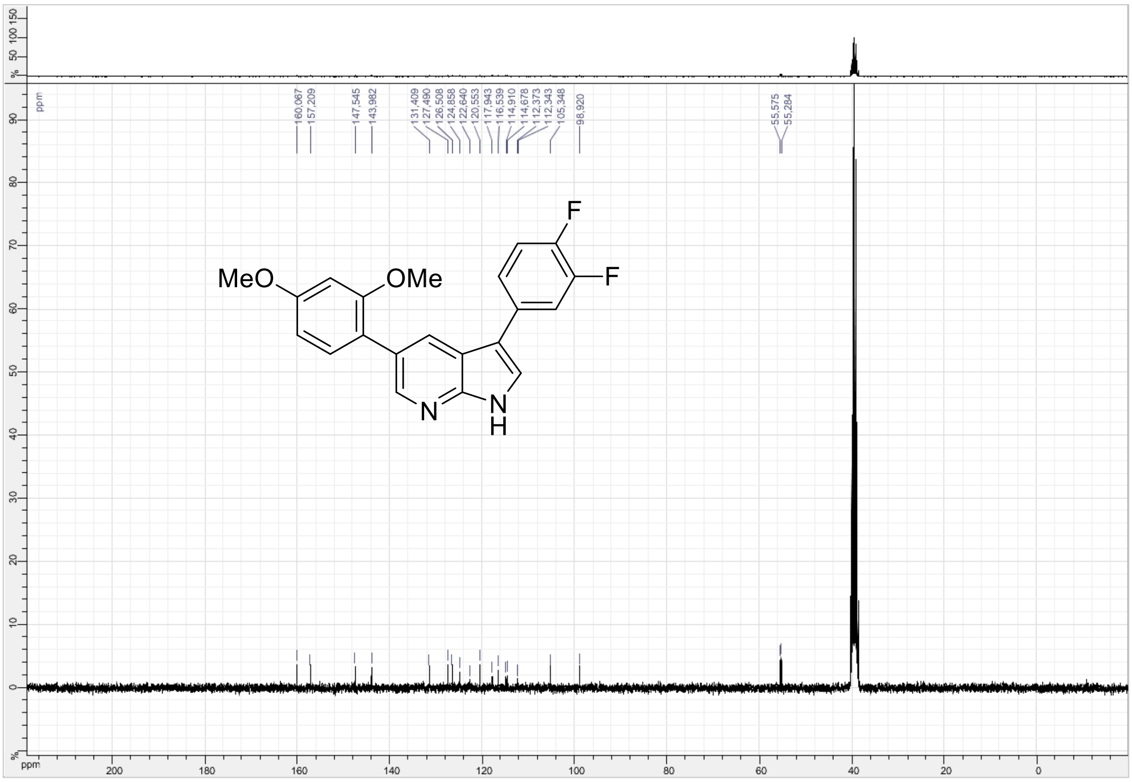

*3-(3-Fluoro-4-methoxyphenyl)-5-(4-benzyloxyphenyl)-1H-pyrrolo[2,3-b]pyridine (**4e**).*

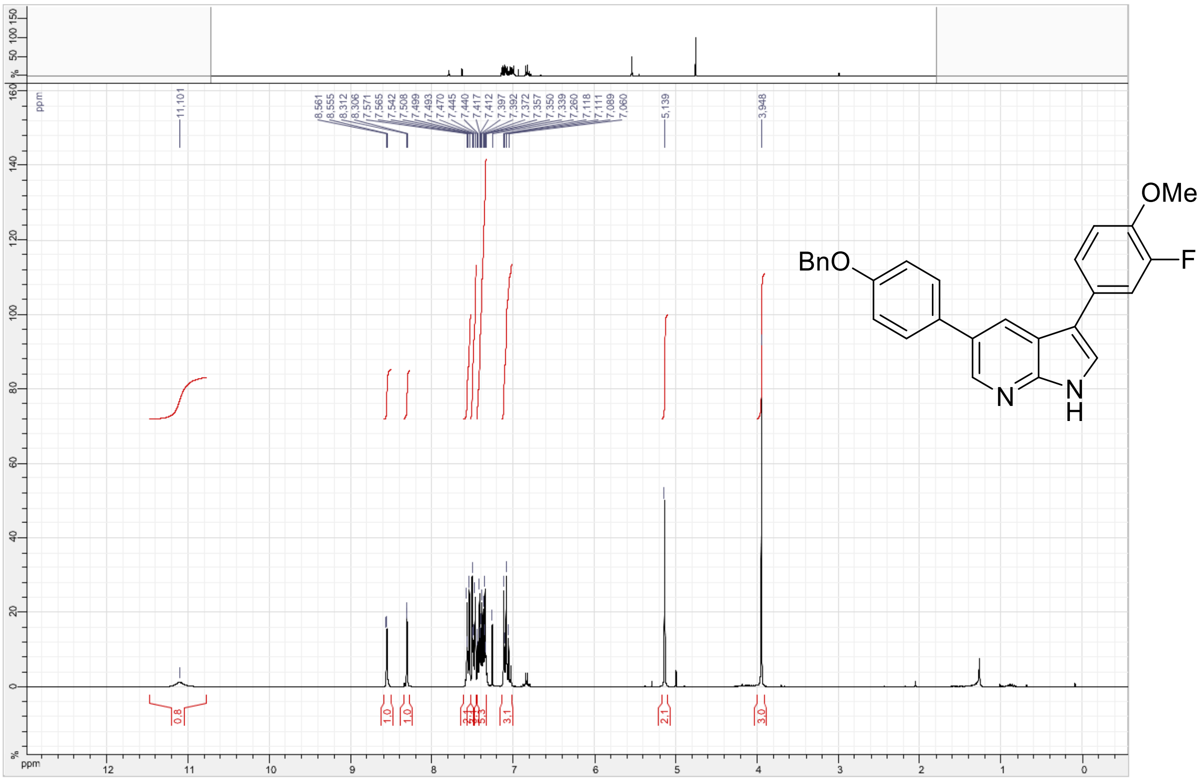

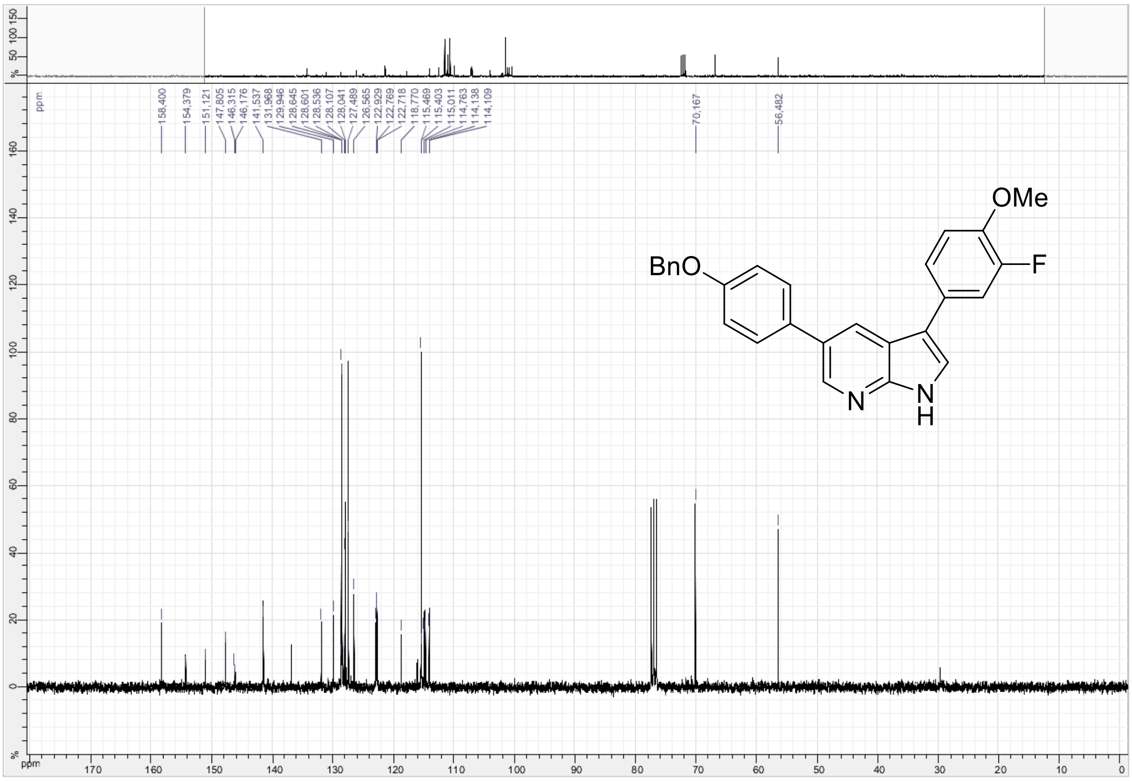

*3-(3,4-Dimethoxyphenyl)-5-(3,4-dibenzyloxyphenyl)-1H-pyrrolo[2,3-b]pyridine (**4f**).*

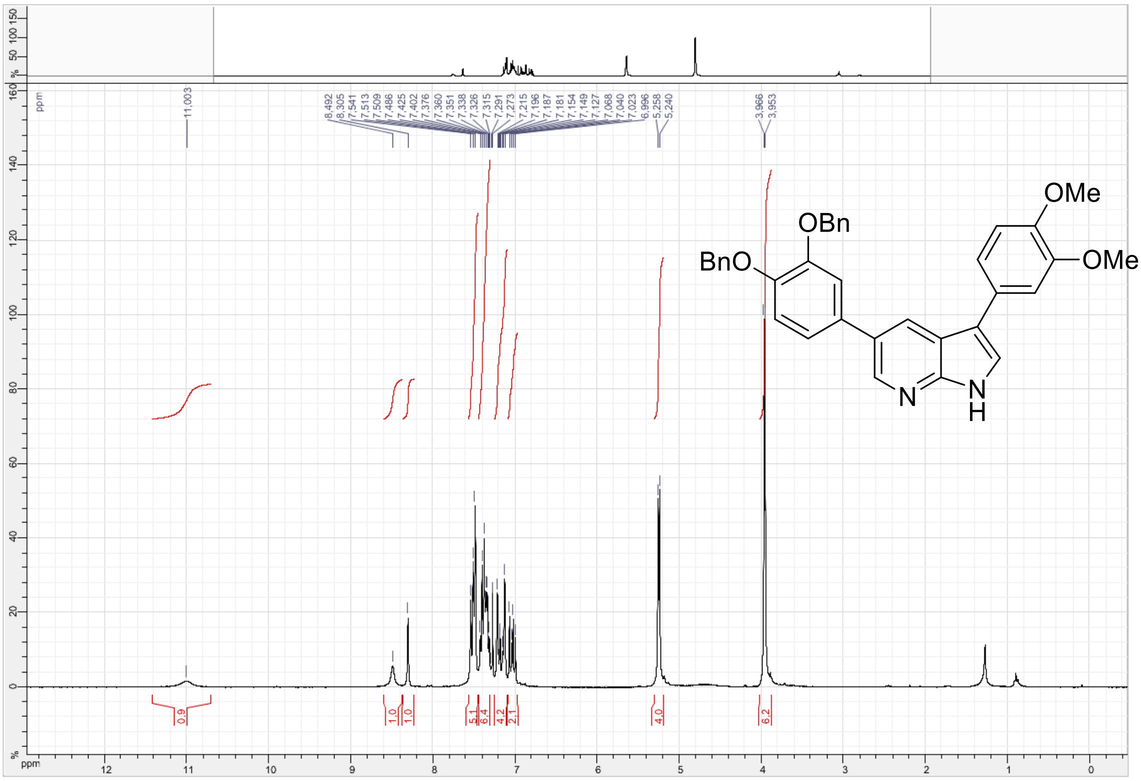

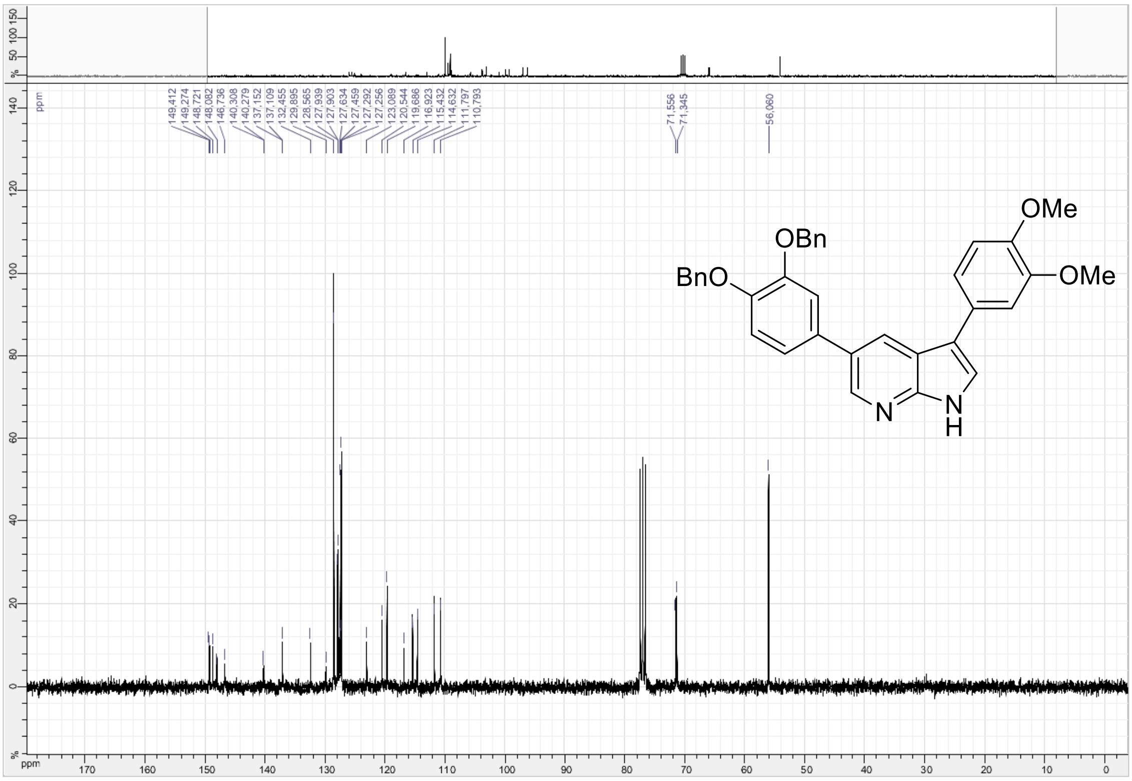

*3-(4-Fluorophenyl)-5-(3,4-dihydroxyphenyl)-1H-pyrrolo[2,3-b]pyridine (**5a**), in DMSO-d6*

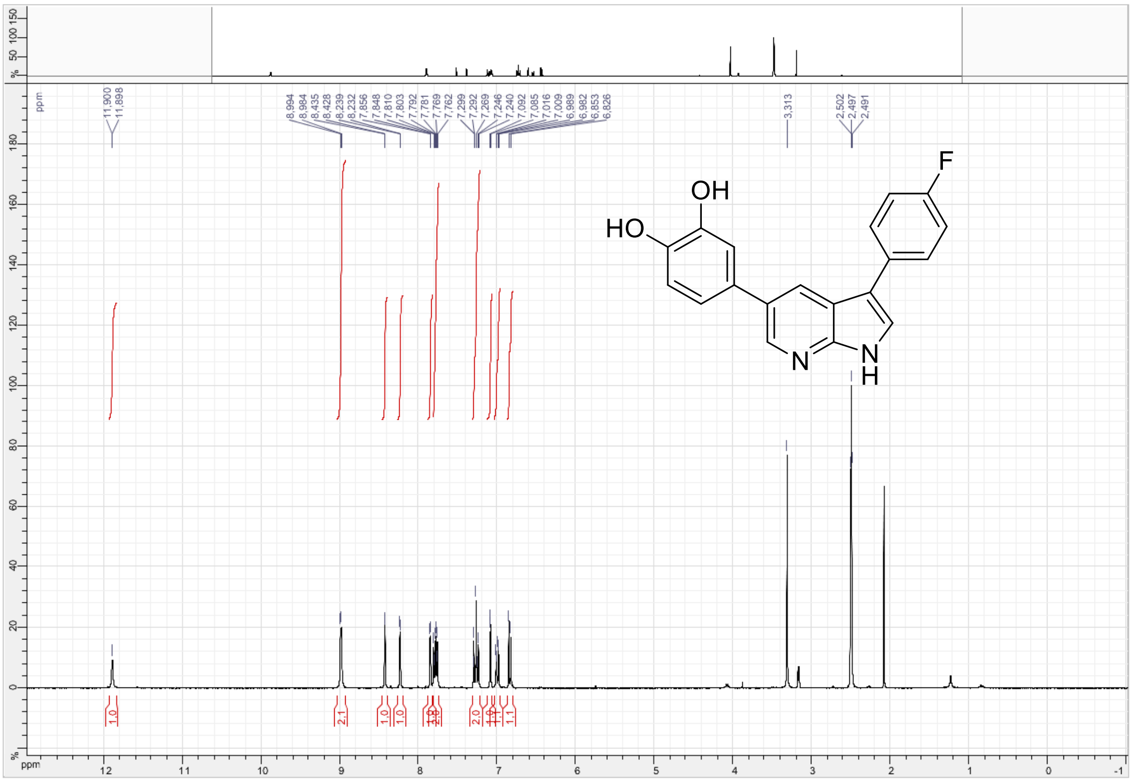

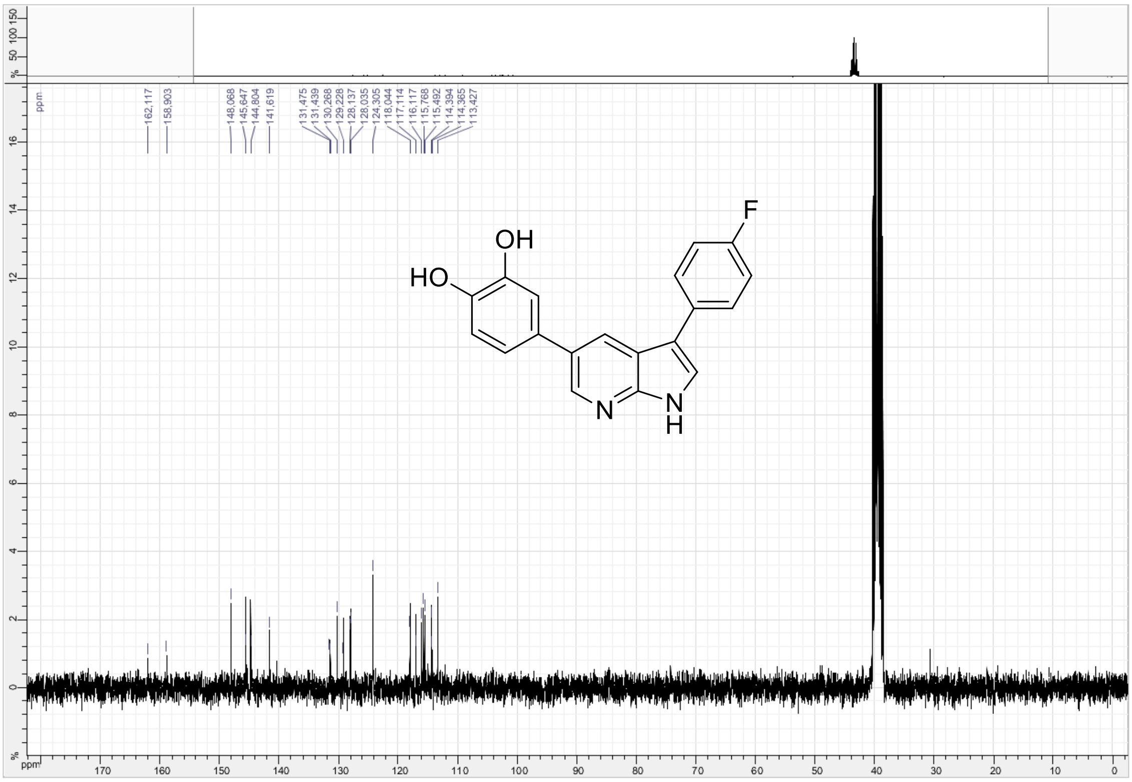

*3-(4-Fluorophenyl)-5-(3,4-dihydroxyphenyl)-1H-pyrrolo[2,3-b]pyridine (**5a**), in CD_3OD*

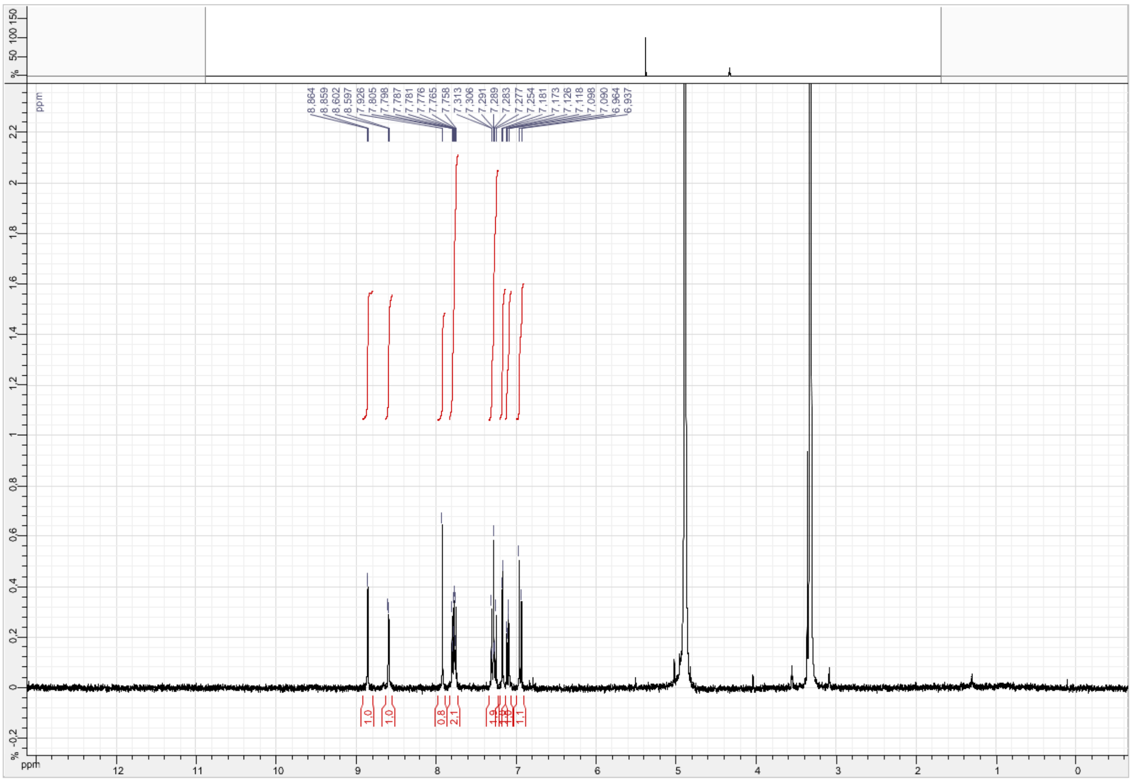

*3-(4-Fluorophenyl)-5-(2,4-dihydroxyphenyl)-1H-pyrrolo[2,3-b]pyridine (**5b**).*

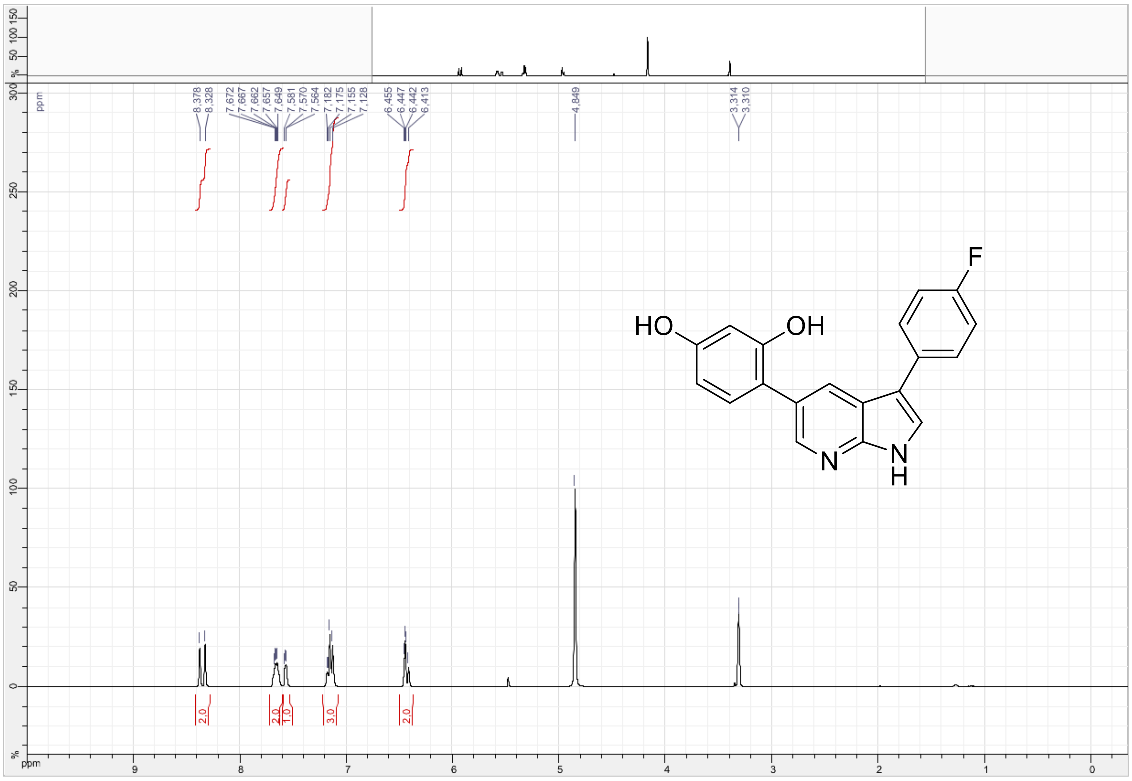

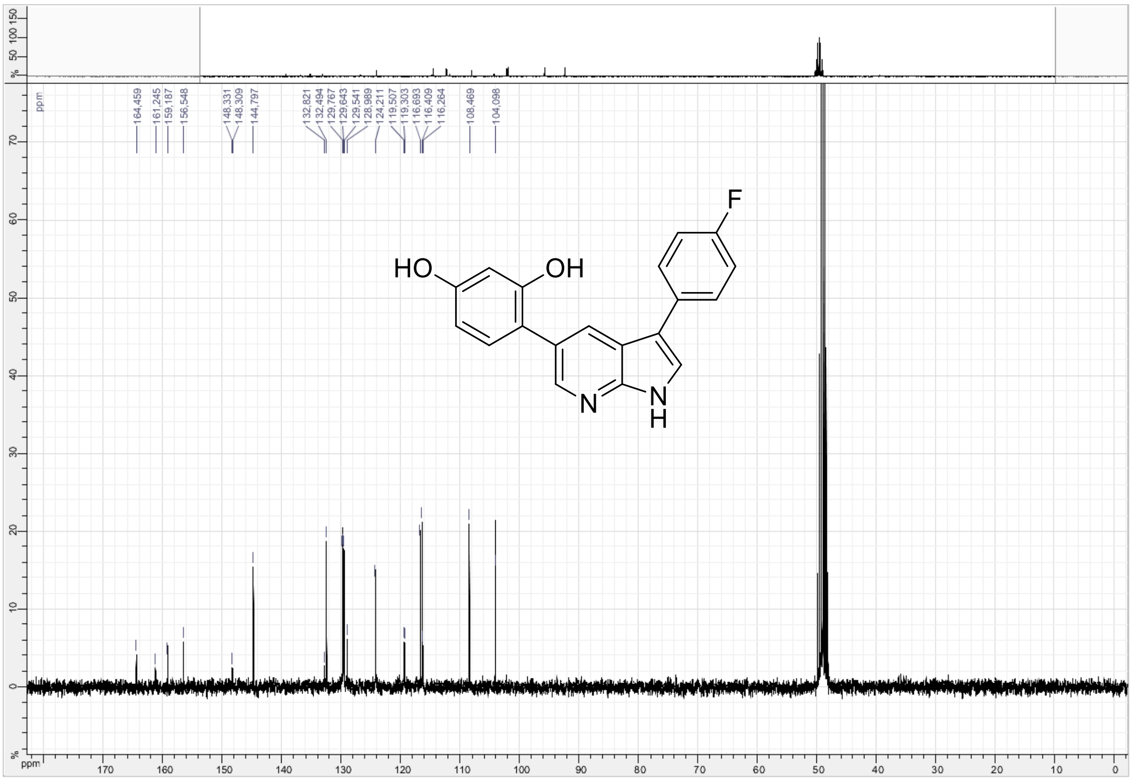

3-(3,4-Difluorophenyl)-5-(3,4-dihydroxyphenyl)-1H-pyrrolo[2,3-b]pyridine (**5c**).

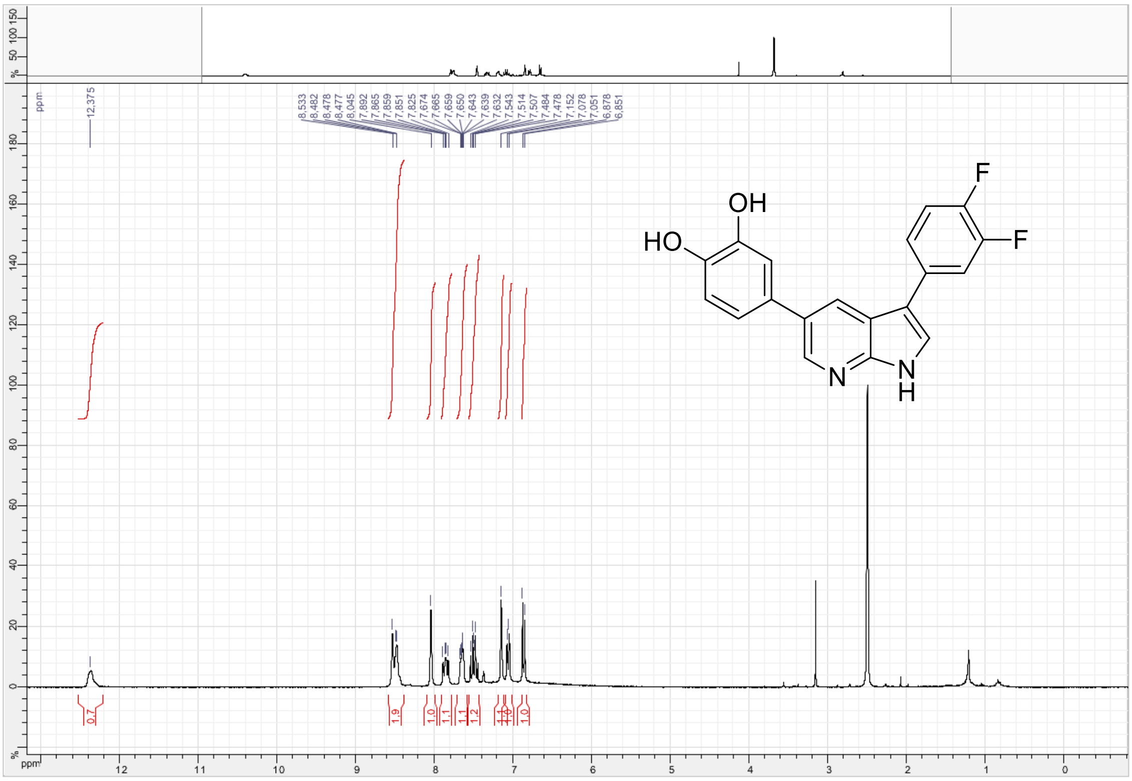

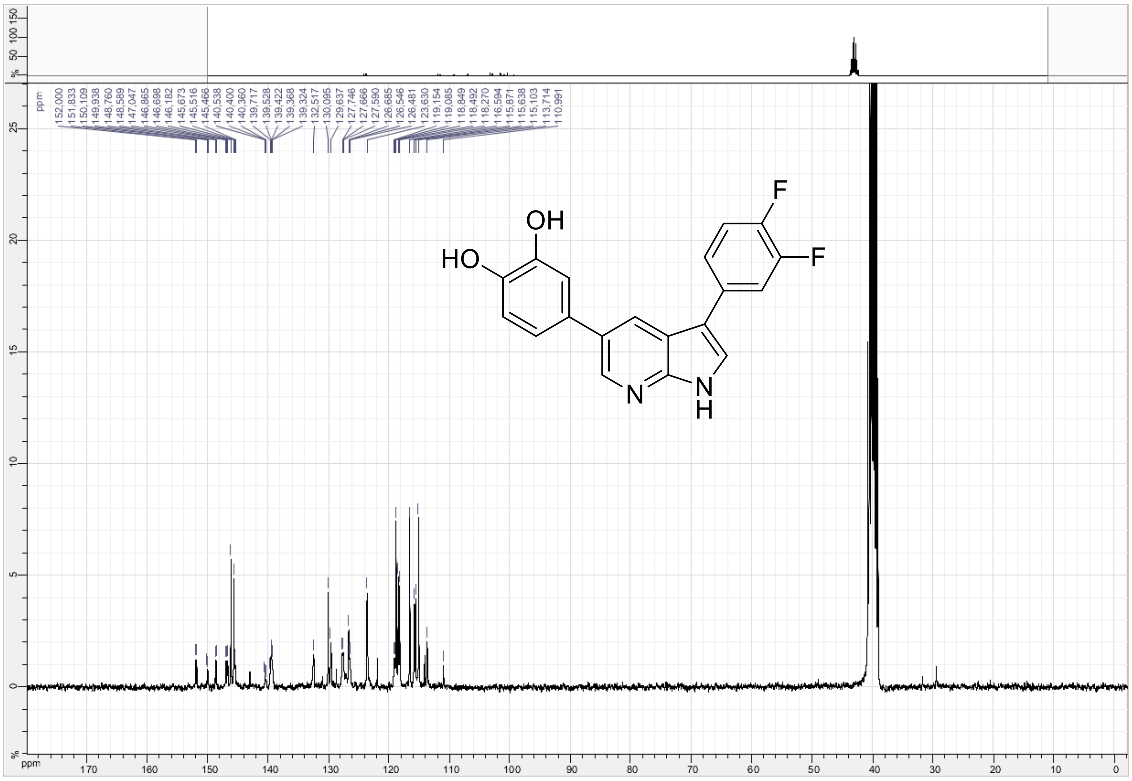

3-(3,4-Difluorophenyl)-5-(2,4-dihydroxyphenyl)-1H-pyrrolo[2,3-b]pyridine (**5d**).

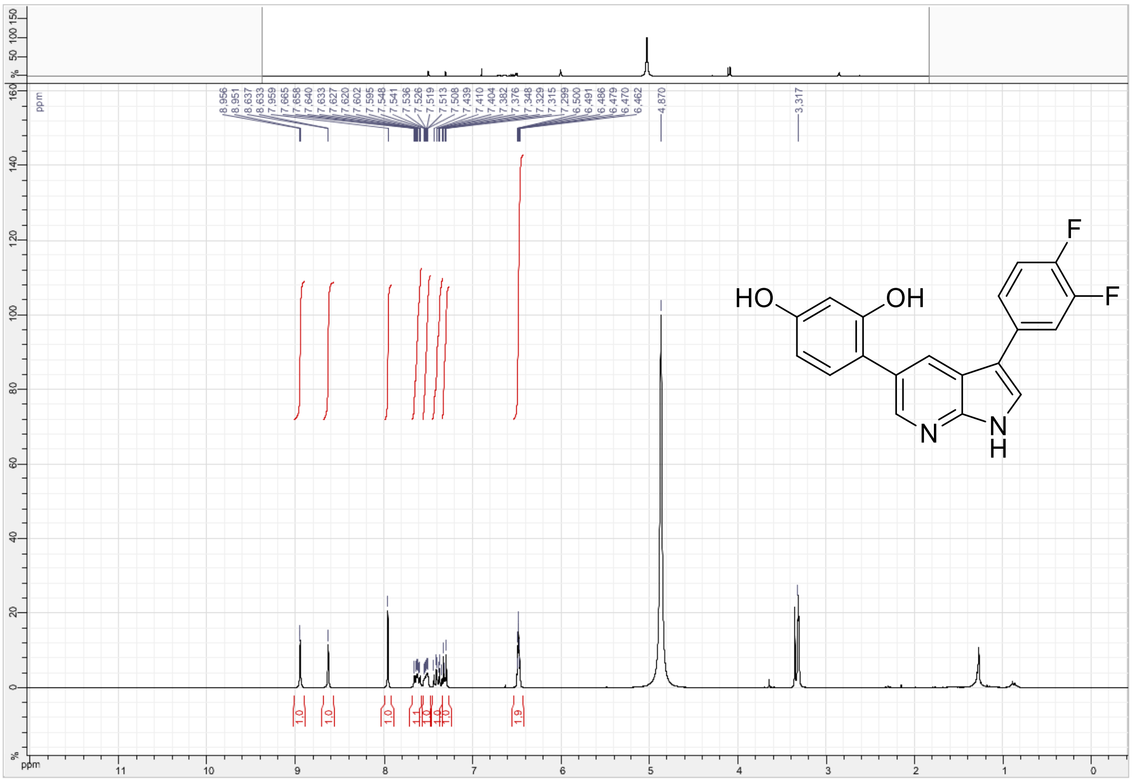

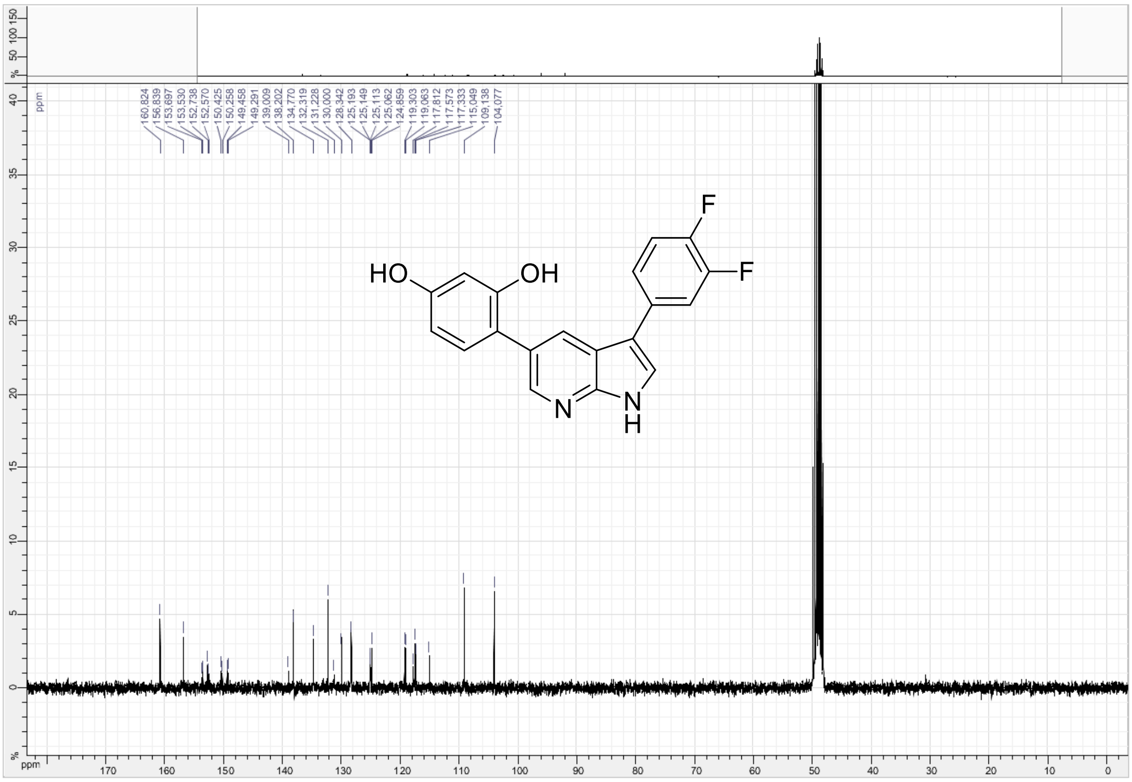

*3-(3-Fluoro-4-hydroxyphenyl)-5-(4-hydroxyphenyl)-1H-pyrrolo[2,3-b]pyridine (**5g**).*

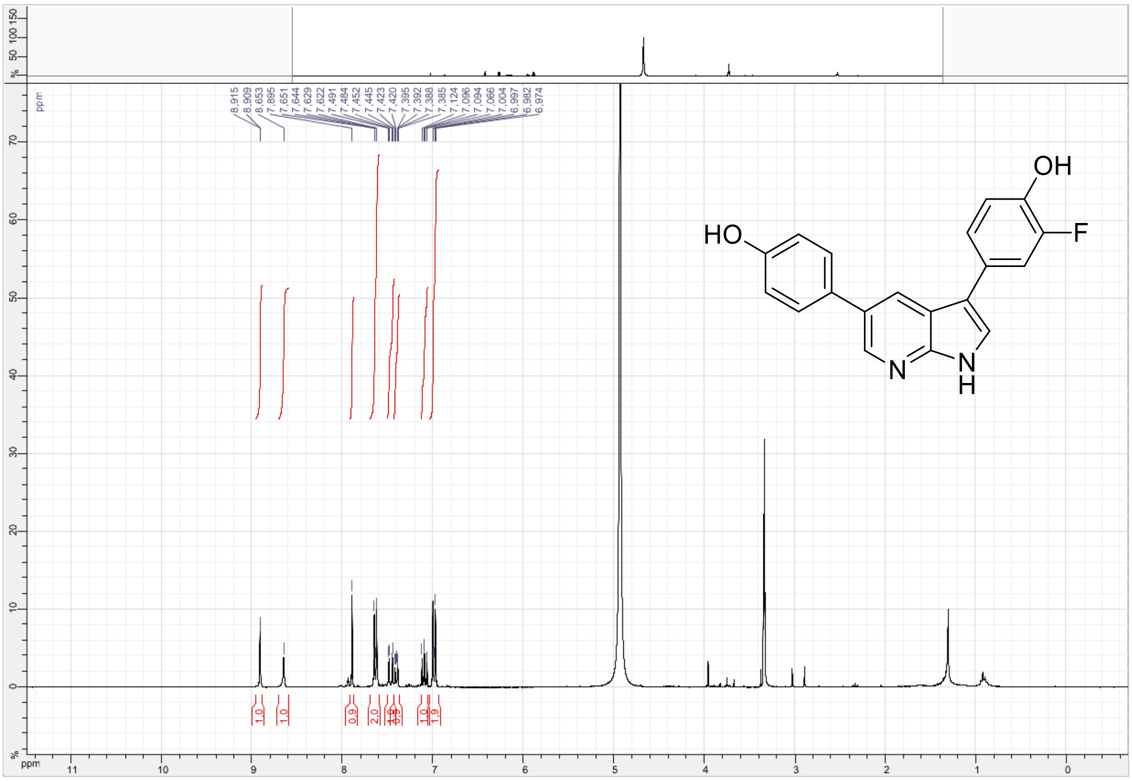

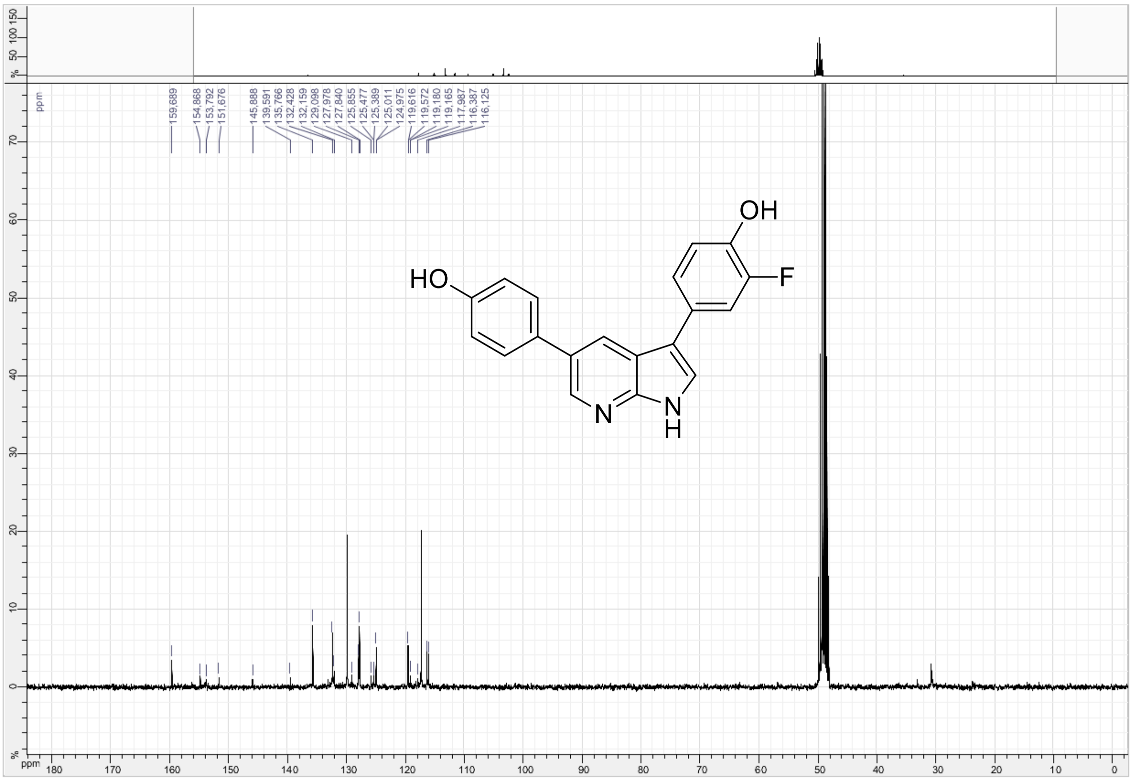

*3-(3-Fluoro-4-methoxyphenyl)-5-(4-hydroxyphenyl)-1H-pyrrolo[2,3-b]pyridine (**5e**).*

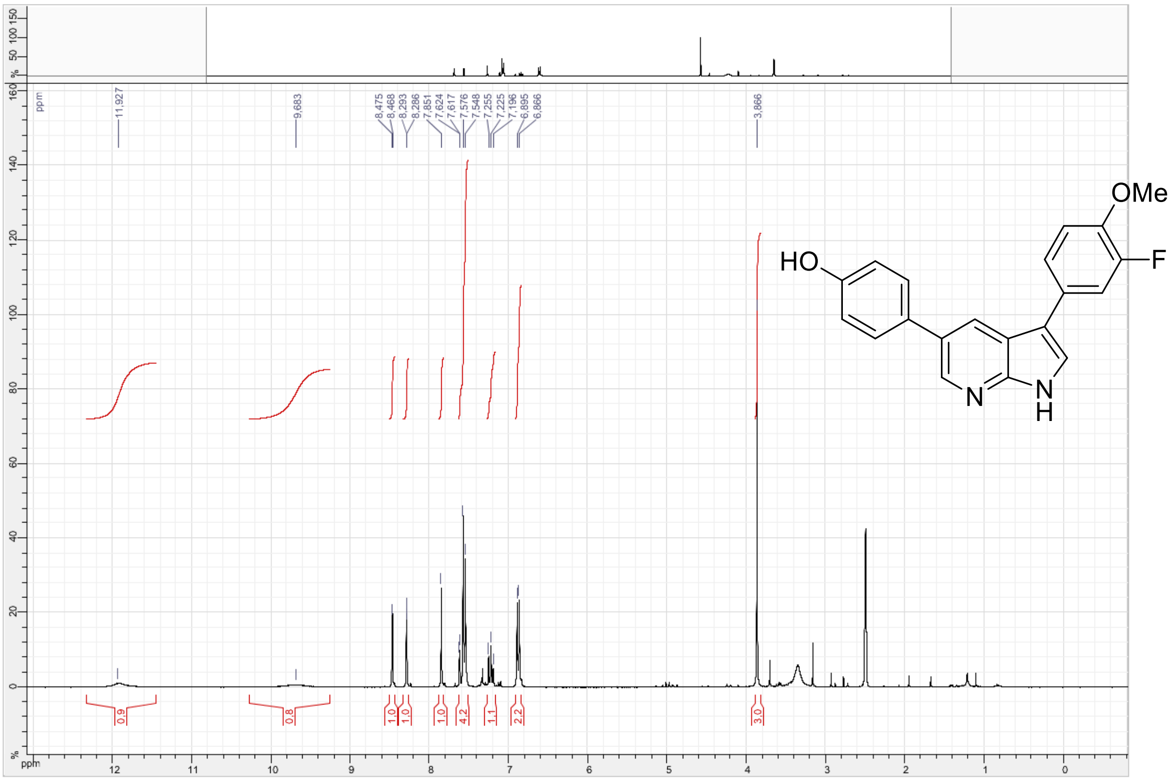

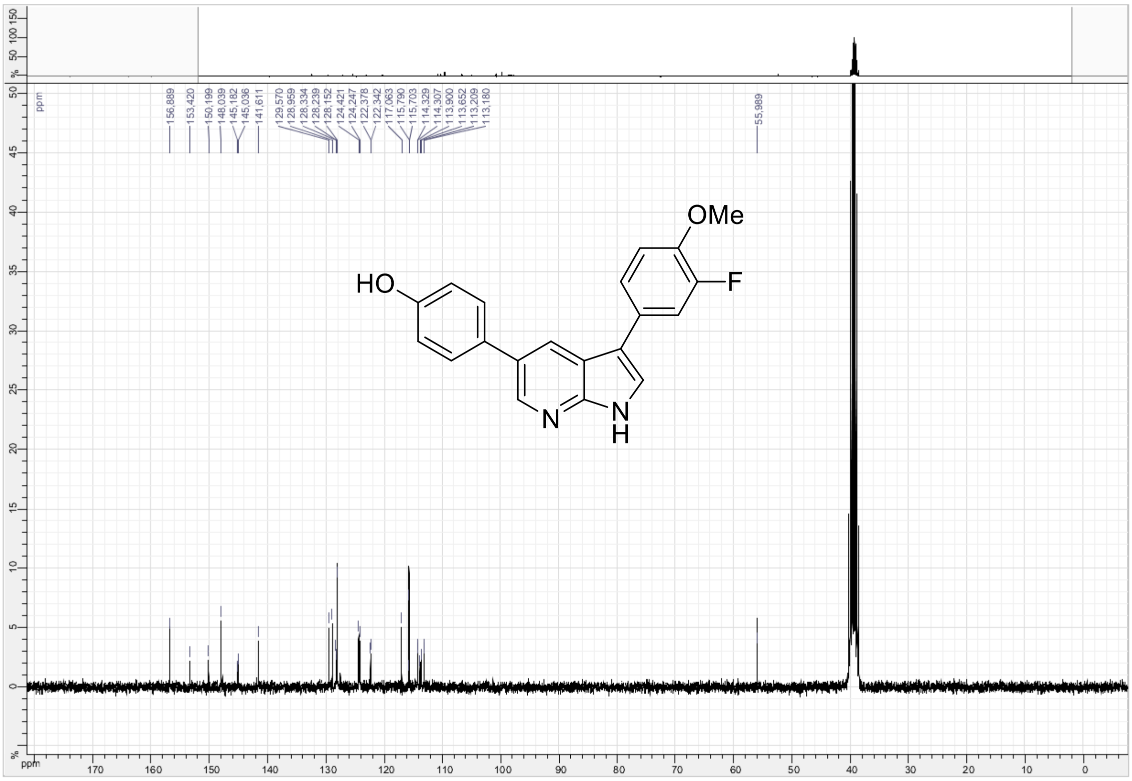

*3-(3,4-Dimethoxyphenyl)-5-(3,4-dihydroxyphenyl)-1H-pyrrolo[2,3-b]pyridine (**5f**).*

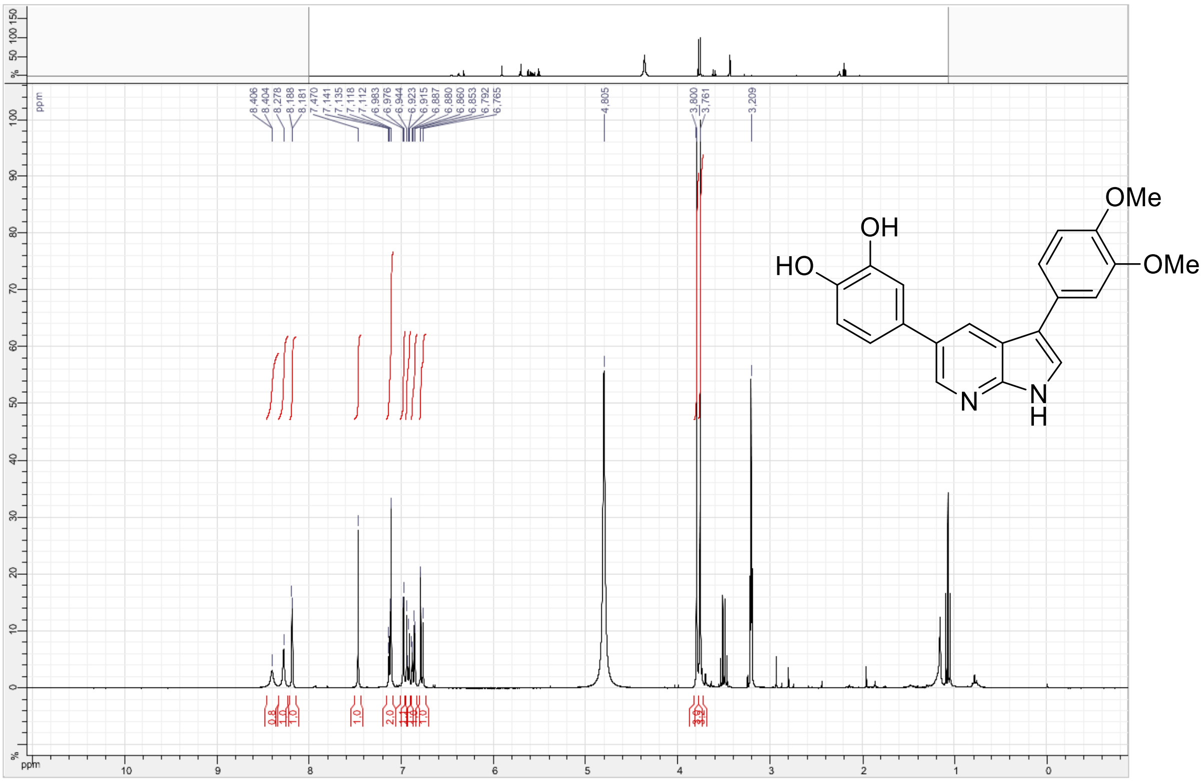

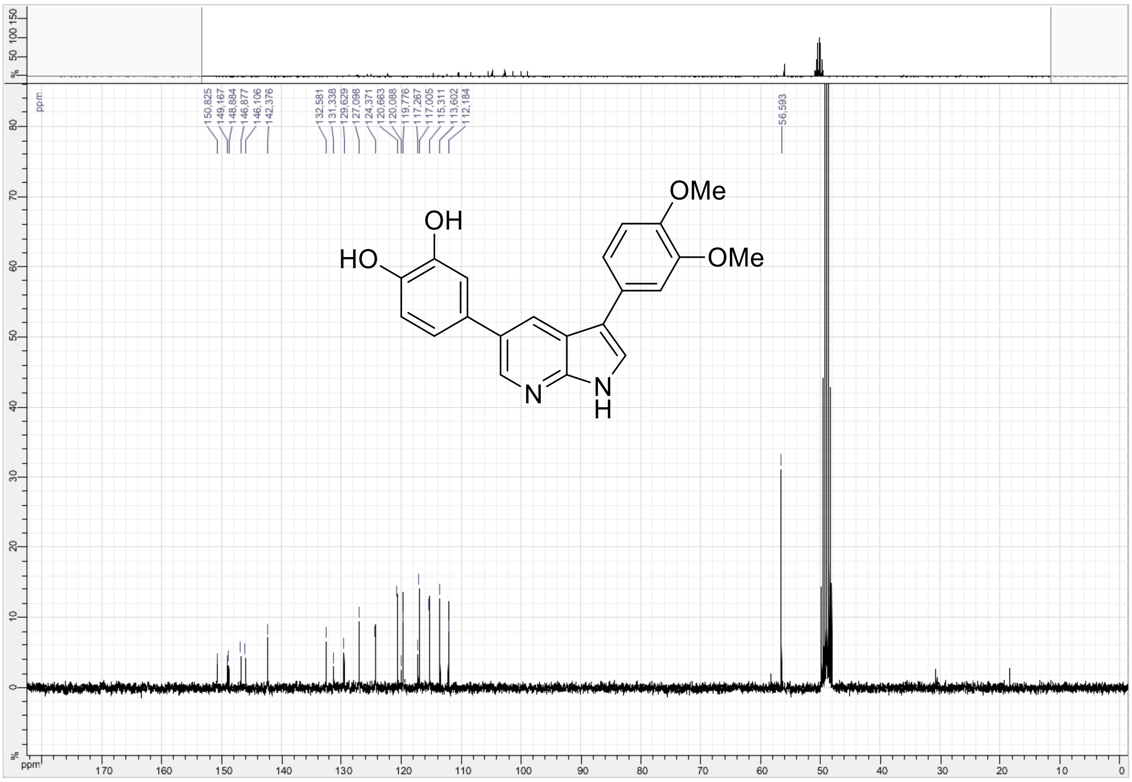

3. Supplementary Figures

Figure SI1: MS/MS spectrum of compound **5a** - parent ion $[M+H]^+$ at *m/z* 321.0 at collision energy 30.

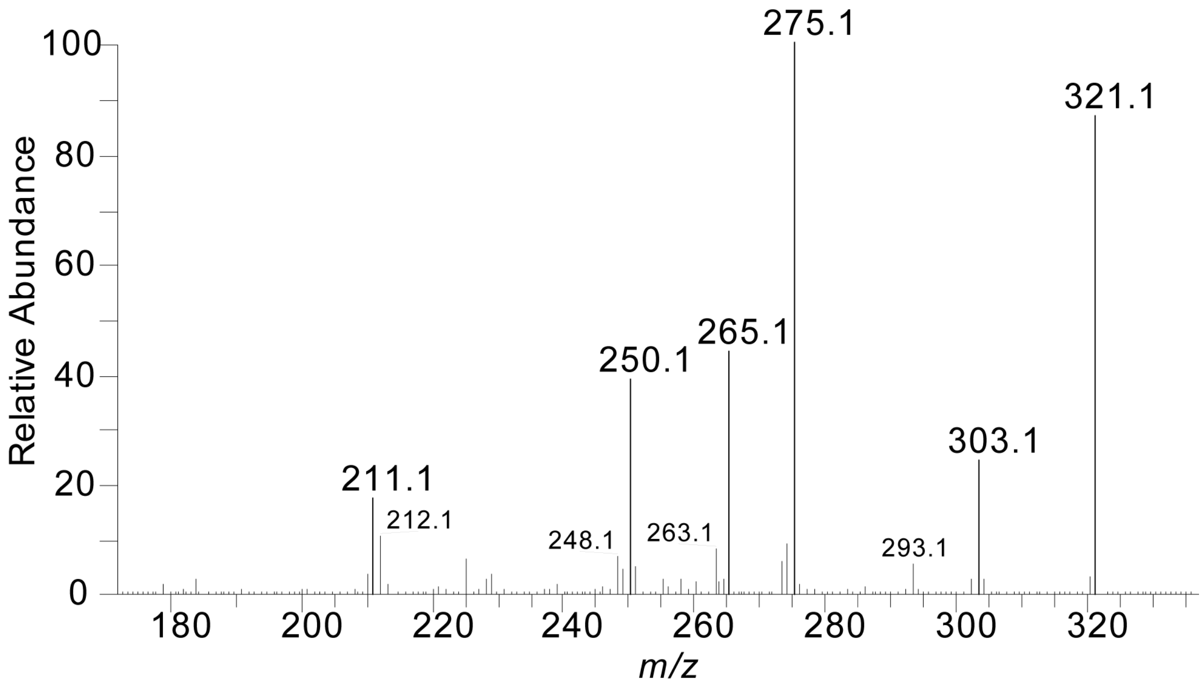

Figure SI2: Extracted Ion Chromatograms for fragment ion at *m/z* 275.0 in plasma (A) and brain (B) samples 1- Ts65Dn treated 2- at the LOQ 3- Ts65Dn vehicle.

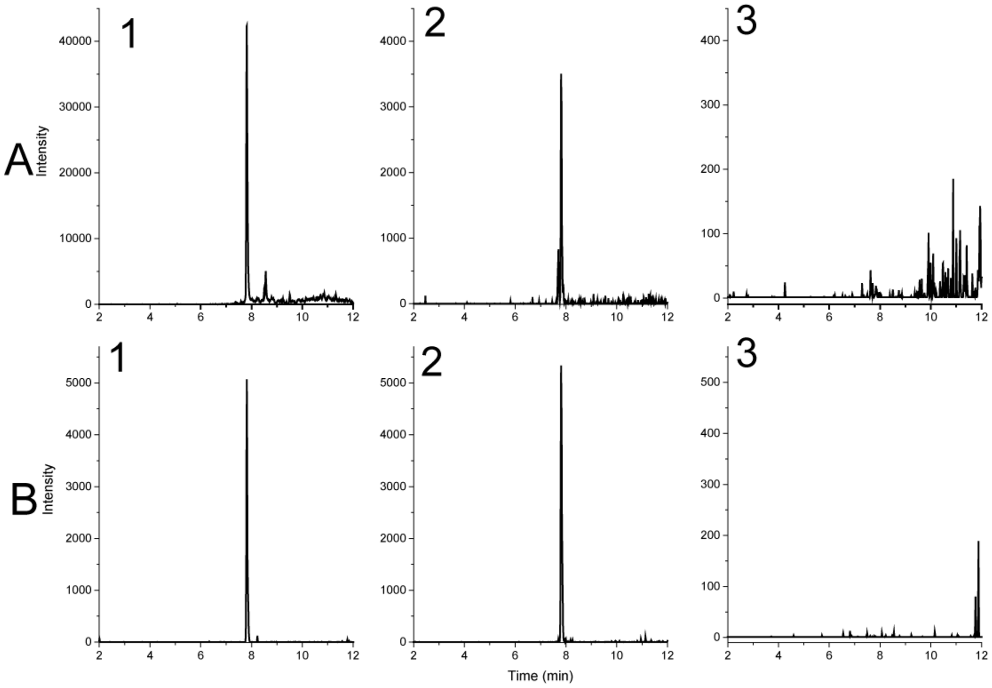

4. Supplementary Table

Table SI1: Optimized SRM transitions for compound **5a** and IS. As acetonitrile adducts were detected, the sixth transition corresponds to this adduct with the following parent ion: **m/z* 344.0 ***m/z* 362.1

Internal standard - Rt = 7.68 min<br>Parent ion *m/z* 303.0		Compound **5a** - Rt = 7.81 min<br>Parent ion *m/z* 321.0		
Fragment ions (*m/z*)	Optimized collision energies (V)	Fragment ions (*m/z*)	Optimized collision energies (V)	Ion Ratio
192.9	33	210.9	34	0.07
231.9	34	250.0	35	0.22
247.1	25	265.0	30	0.17
257.1	28	275.0	31	0.41
285.0	27	303.0	27	0.11
303.0*	15	321.0**	12	0.03
